# Light-driven molecular motors embedded in covalent organic frameworks†

**DOI:** 10.1039/d2sc02282f

**Published:** 2022-06-02

**Authors:** Cosima Stähler, Lars Grunenberg, Maxwell W. Terban, Wesley R. Browne, Daniel Doellerer, Michael Kathan, Martin Etter, Bettina V. Lotsch, Ben L. Feringa, Simon Krause

**Affiliations:** Stratingh Institute for Chemistry, Rijksuniversiteit Groningen Nijenborgh 4 9747 AG Groningen Netherlands; Max Planck Institute for Solid State Research Heisenbergstr. 1 70569 Stuttgart Germany; Department of Chemistry, Ludwig-Maximilians-Universität (LMU) Butenandtstr. 5-13 81377 Munich Germany; Deutsches Elektronen-Synchrotron (DESY) Notkestr. 85 22607 Hamburg Germany; E-conversion Lichtenbergstrasse 4a 85748 Garching Germany

## Abstract

The incorporation of molecular machines into the backbone of porous framework structures will facilitate nano actuation, enhanced molecular transport, and other out-of-equilibrium host–guest phenomena in well-defined 3D solid materials. In this work, we detail the synthesis of a diamine-based light-driven molecular motor and its incorporation into a series of imine-based polymers and covalent organic frameworks (COF). We study structural and dynamic properties of the molecular building blocks and derived self-assembled solids with a series of spectroscopic, diffraction, and theoretical methods. Using an acid-catalyzed synthesis approach, we are able to obtain the first crystalline 2D COF with stacked hexagonal layers that contains 20 mol% molecular motors. The COF features a specific pore volume and surface area of up to 0.45 cm^3^ g^−1^ and 604 m^2^ g^−1^, respectively. Given the molecular structure and bulkiness of the diamine motor, we study the supramolecular assembly of the COF layers and detail stacking disorders between adjacent layers. We finally probe the motor dynamics with *in situ* spectroscopic techniques revealing current limitations in the analysis of these new materials and derive important analysis and design criteria as well as synthetic access to new generations of motorized porous framework materials.

## Abstract 2

The synthesis of a light-driven molecular motor and its incorporation into a series of imine-based polymers and covalent organic frameworks is presented and criteria for the spectroscopic probing of molecular dynamics in porous solids are discussed.

## Graphical abstract

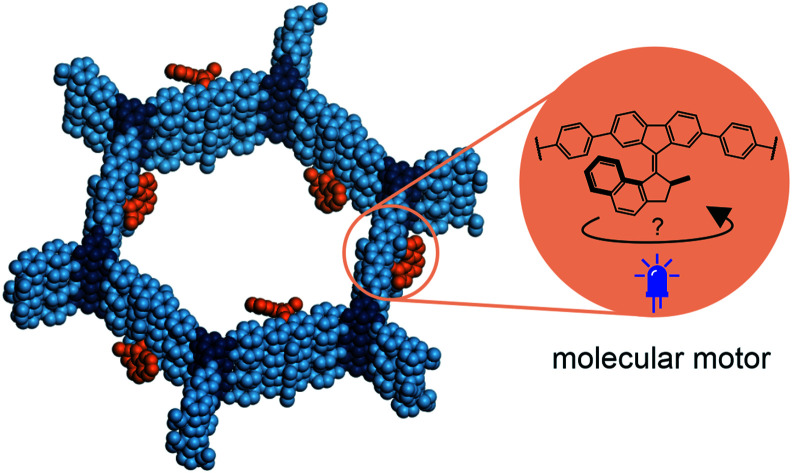

## Introduction

Light-driven molecular motors have emerged as promising platforms to achieve stimuli-responsive motion at a molecular scale.^1–6^ Incorporating these into porous, heterogeneous structures promises new generations of dynamic, light-responsive, smart materials with unique properties that allow control over molecular motion in their surrounding space and pore volume.^7–11^ Maximizing dynamic effects and achieving molecular motion along length scales to create nano- or macroscopic function requires specific designs and diverse conditions, which is a challenging area of contemporary research.^5,12–16^ Besides the control of molecular dynamics, cooperative interactions on a spatiotemporal level and structural aspects of the matrix are also critical design criteria. These include the ratio of dynamically active and passive components in confined space, the three-dimensional structure, adaptivity of the matrix and sufficient void space.

High packing densities of bistable rotaxanes^17^ and catenanes embedded in self-assembled monolayers^18–20^ and polymers^21^ result in a significant decrease in the rate of rotational and translational motion. Similar effects were observed for the intramolecular rotational motion of molecular motors embedded in polymers^7,22^ or grafted onto surfaces.^23,24^ Strategies to avoid these effects include incorporation into low-viscous fluids^25,26^ as well as spatial separation by co-assembly or immobilization of molecular motors into the backbone of porous frameworks^8^ and polymers.^27^ In particular, the latter is a critical innovation towards the transfer of controlled motion of a molecular machine onto a guest species.^28^ The Feringa group recently demonstrated that light driven molecular motors can be embedded in the backbone of metal–organic frameworks by applying a motor-based stator unit.^8^ The rotational frequency of the molecular motors in these nanoporous crystals did not change compared to the molecules' behavior in solution, indicating that the rotor units perform unrestricted motion. In principle, the pore space allows for dynamic confinement effects and unusual transport or adsorption phenomena.^29^ Castiglioni and Feringa *et al.* demonstrated that the porosity of a nanoporous polymer with backbone-embedded, light-driven molecular motors changes reversibly upon irradiation as a result of photoswitch dynamics.^27^ In a computational study, Kolodzeiski *et al.* predicted collective dynamics of framework-embedded molecular motors^30^ and Evans *et al.* were able to show that such properties can lead to activated, directed transport of a confined fluid mimicking a macroscopic pump.^31^ A critical design criterion of such activated directed transport is the presence of pore channels in which the fluid is confined and translational motion is forced in a preferred direction.

A class of materials that feature such intrinsic pore channels and combines it with exceptional structural tunability are 2D Covalent Organic Frameworks (COFs).^32^ The unique properties, such as high porosity, large surface areas, light-absorption, and ordered structuring, are combined within a crystalline polymeric material. COFs have been used for diverse applications, including gas storage and separation, sensing, electrochemical energy storage, and heterogeneous (photo)catalysis.^33–39^

Recently the incorporation of light-responsive moieties in COFs has gained interest for modifying their properties, *e.g.* pore-accessibility, by external stimulation. To this end, various polymers^40^ and COFs comprising azobenzene-^41,42^ or dithienylethene-switches^43^ as building blocks have been synthesized. Apart from these switches, rotaxanes have also recently been introduced into COF-like polymers.^44,45^ A major challenge in these structures is the compromise between structural complexity, functionality and crystallinity of the material,^46^ with a loss of structural order evident in the X-ray diffraction pattern and low surface area with geometrically more complex building blocks.^44,45^ In contrast to MOFs, COFs are connected entirely by covalent bonds, and many COFs exhibit a higher chemical stability than MOFs.^47^ The combination of low density, structural tunability and stability makes COFs more attractive for the integration of molecular machines. As an example, many MOF structures are sensitive to solvent removal, drastically limiting their potential for applications.^48^ COFs furthermore perform better in terms of processability and in principle allow for hierarchical structuring, such as thin-film fabrication, required for advanced (photo-)electrochemical applications.^49,50^ Despite its structural elegance and advantages, molecular motors have not been embedded in COFs to date.

Contrary to amorphous polymeric materials, COFs allow, in principle, for precise arrangement of responsive molecules within their crystalline framework, similar to the arrangement demonstrated in MOFs.^8^ The spatial arrangement of molecular machines is crucial to amplify and coordinate their movement across multiple length and time scales.^12^ Molecular motors arranged in amphiphiles could be used as artificial muscles, able to lift macroscopic objects.^51^ Motors as dopants in liquid crystals, for example, are able to move micrometer sized objects through spatial rearrangement of the liquid crystal matrix.^26^ In these systems only small amounts of molecular motors were embedded, demonstrating that the quantity of machines integrated is less relevant than the quality of their arrangement to obtain responsive function.

Herein we detail design principles and synthetic strategies of COFs featuring backbone-embedded light-driven molecular motors. To the best of our knowledge, this is the first report of a light-driven molecular motor covalently incorporated in 2D COFs, in which both crystallinity and porosity are preserved. We synthesize a new diamine light driven molecular motor and investigate its dynamics with *in situ* spectroscopic methods. By utilizing different reaction conditions, we synthesize amorphous polymers as well as crystalline 2D COFs, constructed from this diamine molecular motor *via* imine condensation. The structural features of the self-assembled frameworks with respect to composition, local and long-range structure, structural disorder, and porosity were investigated. We elucidate the stacking mechanism of adjacent 2D layers in the framework and examine the motor behavior with FT-IR, Raman and UV/vis spectroscopy. From this, we derive design principles and methodologies that in the future will help to establish dynamic COFs to be used as light-responsive smart materials.

## Results

**Fig. 1:**
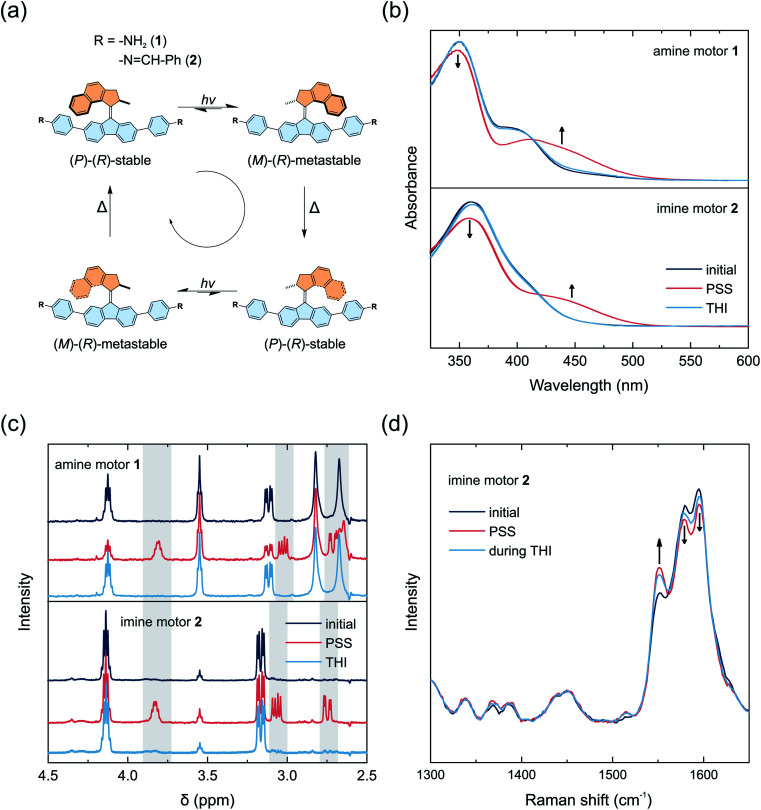
(a) Configurational changes upon photoswitching and thermal helix inversion of motors 1 and 2. (b) UV/vis absorption spectra upon irradiation of motors 1 and 2 in acetonitrile at 5 °C. The initial absorption spectra are shown in dark blue. The red spectra correspond to the photostationary state (PSS) at 395 nm for motor 1 and 365 nm for motor 2. The spectra after thermal helix inversion (THI) are shown in light blue. (c) ^1^H NMR spectra of motors 1 and 2 in the dark (blue) and after irradiation (red) in deuterated benzene at 10 °C. Spectra of the PSS show an additional set of signals for the metastable states (grey). (d) Raman spectra (785 nm) of motor 2 in toluene in the dark (blue), after irradiation with 365 nm (red) and after thermal helix inversion (light blue).

The rotational cycle of overcrowded alkene-based molecular motors consists of four distinct steps.^52,53^ First, the C–C double bond connecting the two halves of the molecule functions as the rotary axle. It undergoes an *E*–*Z* isomerization upon irradiation with light in the UV/vis region. The molecule adopts a metastable state, in which the methyl group of the upper half (Fig. 1a, orange) converts from a pseudo axial to an energetically less favored pseudo equatorial position. Subsequently, the molecule relaxes through a thermal helix inversion, completing a 180° rotation. This step is irreversible, in contrast to the double bond isomerization step in which a photochemical equilibrium is established. The thermal helix inversion therefore ensures the unidirectionality of the motor rotation. The half-life of the metastable state is furthermore the main determining factor for the rotational speed of the motor. Repetition of the photochemical and thermal steps fulfills a full 360° rotation (Fig. 1a).

Depending on the structural features of overcrowded alkene-based molecular motors, their properties like absorption wavelength, rotational speed, or thermal half-life of the metastable state can vary tremendously.^5^ The half-life of the metastable state is primarily determined by the ring size of the two moieties connected by the central C–C double bond. For our studies, we chose a synthetically easily accessible molecular motor structurally related to the MOF-embedded, pyridine-derived motor detailed by Danowski *et al.*^8^ This molecular motor features a half-life in the range of minutes to be able to conveniently study the rotation at room temperature.^54^

**Fig. 2:**
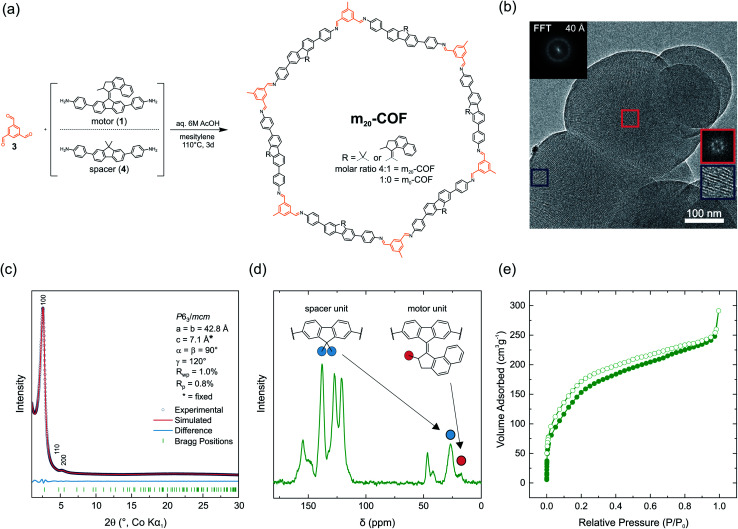
(a) Synthesis of m_20_-COF from mixtures of spacer and motor building blocks with triformylbenzene (3) under solvothermal conditions. (b) Transmission electron microscopy (TEM) image of m_20_-COF with visible pore channels along [00*l*] pointing towards the surface of the spherical particle (blue inset). Fast Fourier transformed (FFT) insets show a hexagonal pattern (red box, viewing direction [00*l*]). (c) XRPD pattern and unit cell parameters of m_20_-COF obtained by Pawley refinement with a simplified unit cell proxy model (see Table S1†) (d) ^13^C-CPMAS ssNMR spectrum of m_20_-COF containing signals of the motor and spacer building blocks. (e) N_2_ adsorption isotherm of m_20_-COF.

Imine based dynamic covalent chemistry^55,56^ has emerged as the most popular handle for making functional COFs,^57^ as boronic acids are prone to hydrolysis.^58^ This approach requires amine or aldehyde-based building blocks and we selected the aniline-based bisfunctional molecular motor 1 (Fig. 1a, R = NH_2_) as a suitable building block for the synthesis of COFs and 1,3,5-triformyl benzene (3) as the aldehyde counterpart (Fig. 2a). Due to steric bulk of the motor unit, we used a larger amine building block, which is extended by aniline moieties, compared to recent examples of fluorene based COFs,^59,60^ creating a larger pore size in the condensed network and void volume in the motor plane. The selected building block combination allows condensation into a 2D layered COF, which is not prone to form interpenetrated structures as reported for many 3D COFs. Although 3D COFs may appear structurally more related to MOF structures, these materials are often obtained with reduced pore volume due to interpenetration, detrimental for the rotation of the motor unit.^61^

In order to study framework formation and properties independent of molecular motors, we chose to establish a structurally similar dimethyl fluorene spacer without motor functionality. This spacer mimics the lower half of the motor (Fig. 1a, blue), also allowing integration of both molecular building blocks within the same framework. Co-condensation in a single framework^62^ enhances the free space in which the motor can rotate. The methyl groups of the spacer further assist in increasing free volume for the motor rotation by favoring an antiparallel arrangement between neighboring fluorene units.^63–65^

### Synthesis and characterization of the building blocks

The amine-functionalized spacer 4 and motor 1 were synthesized following a convergent strategy. A motor precursor was synthesized in a Barton–Kellogg olefination between the respective thioketone and 2,7-dibromo-9-diazafluorene following our reported procedure.^8^ The amine functionality in the motor 1 was subsequently introduced in a Suzuki-cross coupling reaction with 4-aminophenylboronic pinacol ester (for synthetic details, see ESI†). The same procedure was followed to afford the amine-functionalized spacer 4. A model imine compound 2 (Fig. 1a) was prepared and investigated additionally to study the rotary behavior of the motor 1 after imine condensation in solution. This model compound also mimics the chemistry of the motor and local molecular sterics when incorporated into the backbone of the COF. The model compound 2 was prepared by reaction of the amine building block 1 with benzaldehyde in toluene with 3 Å molecular sieves.

The light-induced rotational behavior of molecular motors 1 and 2 was monitored *in situ* using UV/vis absorption and proton nuclear magnetic resonance (^1^H NMR) spectroscopy (Fig. 1b–d). We additionally investigated a solution of imine functionalized motor 2 by Raman spectroscopy to probe the dynamics of molecular motors in solution and in the solid state as previously shown for MOFs.^8,11^

A solution of amine functionalized motor 1 in acetonitrile initially shows a local absorption maximum at 400 nm (Fig. 1b). Upon irradiation at 395 nm at 5 °C, a new band with a bathochromic shift formed at 450 nm, indicating the formation of a metastable state typically observed for second generation light-driven molecular motors.^4^ An isosbestic point at 415 nm indicates that a single step process takes place (Fig. S2†). Relaxation of the sample at 5 °C after irradiation led to the reversible recovery of the stable isomer. Eyring analysis yielded an activation barrier for the thermal helix inversion of 87.9 kJ mol^−1^, which corresponds to a thermal half-life of the metastable state of 8.6 min at 20 °C (Fig. S2†).

A solution of imine functionalized model motor 2 in acetonitrile exhibited an absorption maximum at 360 nm, and irradiation at 365 nm led to the formation of a new band at 440 nm, indicating the formation of the metastable state, while maintaining an isosbestic point at 420 nm (Fig. 1b and S3†). The stable state was fully recovered after relaxation in the dark for approximately 2 h (Fig. S26†). The activation energy of the thermal helix inversion was determined to be 88.3 kJ mol^−1^, which corresponds to a thermal half-life of the metastable state of 10.3 min at 20 °C (Fig. S3†). Based on this analysis we have confirmed both motors 1 and 2 to exhibit light-responsive rotational dynamics that are observable under ambient conditions.

The nature of the isomers formed was further investigated by ^1^H NMR spectroscopy (Fig. 1c). Solutions of both motors 1 and 2 dissolved in deuterated benzene were irradiated *in situ* at 10 °C. Irradiation of motor 1 at 385 nm led to the formation of a new set of signals, indicating the formation of the metastable isomer at a photostationary state with a ratio of 54 : 46 (metastable : stable). Leaving the sample at 10 °C without irradiation led to recovery of the initial spectrum of the stable state (Fig. S23 and S24†). Imine functionalized motor 2 was irradiated at 365 nm and reached a ratio of 22 : 78 (metastable : stable) at the photostationary state. After leaving the sample at 10 °C in the dark, the spectrum of the stable state was fully recovered (Fig. S25 and S26†).

Investigation of motor 2 by Raman spectroscopy in toluene showed the appearance of a band at 1550 cm^−1^ upon irradiation at 365 nm, which is typical for the stretching of the central C–C double bond of the metastable isomer (Fig. 1d).^66^ The band diminishes while leaving the sample without irradiation at room temperature, demonstrating reversible thermal helix inversion. The energy barriers for the thermal helix inversion of metastable motors 1 and 2 are in line with values computed by DFT (Tables S2 and S3†). In both cases, the geometry of the fluorenyl moiety, which is embedded in the framework backbone, undergoes only a minor deformation upon isomerization and should allow rotation while preserving the framework. The imine functionalized motor 2 has a half-life of several minutes at room temperature (Fig. S3 and S26†), which is ideal to follow the motor rotation at room temperature in solution and in the solid framework. Motor 2, however, exhibits a less favorable ratio between the stable and metastable state at PSS, with a preference towards the stable state. DFT studies show that the lower halves of the building blocks are bend by *ca.* 18° (Table S4†) and the motor adds structural complexity and bulkiness, making their condensation into a crystalline COF challenging. Consequently, we followed two different approaches for the condensation of building blocks into COFs.

### Materials synthesis

The amine building blocks were condensed with 1,3,5-triformyl benzene (3) under either of two reaction conditions. A Sc(OTf)_3_ catalyzed reaction^67,68^ of the building blocks 1 and 4 with 1,3,5-triformyl benzene (3) in mesitylene : 1,4-dioxane (1 : 4) at room temperature led to fast precipitation of an amorphous, polymeric material. Three different polymers with varying amount of motor were prepared: a pure motor polymer (m_100_-P), in a 1 : 1 ratio with the spacer (m_50_-P), and a pure spacer polymer (m_0_-P). After the condensation, the materials were washed with ethanol and centrifuged several times to remove residual building blocks and dried with supercritical CO_2_. The formation of the imine backbone of the polymer and the consumption of trisaldehyde 3 and amine building blocks 1 and 4 is confirmed by Fourier transform infrared (FT-IR) spectroscopy, showing the characteristic imine band (*v*_C=N_) at 1623 cm^−1^ (Fig. S4†). All three IR spectra show similar bands in the finger-print region, underlining the structural similarity of the polymers.

Solid-state nuclear magnetic resonance spectroscopy (ssNMR) was performed to confirm the incorporation of the building blocks and preservation of motor integrity within the polymer backbone. The ^13^C ssNMR spectrum of m_0_-P shows three characteristic signals for the imine carbon atom (156.4 ppm), the aliphatic bridging atom (48.3 ppm) and the methyl groups (28.3 ppm) (Fig. S27 and S28†). m_100_-P shows two characteristic peaks in the aliphatic region at 41.6 ppm and 18.2 ppm and a broad signal at 148.5 ppm for the imine carbon (Fig. S29 and S30†). The two sets of signals can be identified in the spectrum of m_50_-P confirming that both building blocks are incorporated within the polymer and the motor molecules are intact (Fig. S31 and S32†). X-ray powder diffraction (XRPD) revealed that all three materials are missing long-range order with only the m_0_-P exhibiting a single broad, poorly defined reflection at 2*θ* = 2–4° (Co-K_α1_), indicative of only weak structural coherence within the material (see Fig. S13†). These findings are consistent with the low Brunauer–Emmett–Teller (BET) surface areas of 25.3 m^2^ g^−1^ (m_100_-P) and 10.2 m^2^ g^−1^ (m_50_-P) for the motor containing polymers, and moderate values of 713 m^2^ g^−1^ for the pure-spacer polymer m_0_-P, probed by N_2_ sorption analysis at 77 K (Fig. S39–S41 and S44–S46†). Furthermore, the total pore volumes for these materials are 0.04 cm^3^ g^−1^, 0.03 cm^3^ g^−1^ and 0.42 cm^3^ g^−1^, respectively, showing porosity only for the non-motor containing polymer m_0_-P. However, given the lack of long-range order, the local molecular environment in these polymers is unknown.

To realize a porous, motor-containing material, we made use of an orthogonal solvothermal synthesis approach with aqueous 6 M acetic acid as a catalyst to enhance crystallinity.^69^ With this approach m_20_-COF was crystallized from motor 1 and spacer 4 amine building blocks (4 : 1 molar ratio) and 1,3,5-triformyl benzene (3) in mesitylene as the solvent at 110 °C for 72 h (Fig. 2a). This ratio of spacer and motor building blocks refers to a statistical loading of 1.2 motors per hexagonal pore in two layers, and thus ensures free void for the motor rotation. XRPD with Co-K_α1_ radiation shows two Bragg-like peaks at 2*θ* = 2.6° and 5.3°, indexed as 100 and 200 reflections using a crystalline eclipsed stacked structure as a proxy model (Fig. 2c and S12†) (space group *P*6_3_/*mcm*). This supports the in-plane order of the layers, in spite of the random motor distribution. A Pawley refinement gives unit cell parameters *a* = *b* = 42.8 Å (Fig. 2c). Notably, due to the absence of any stacking reflections, the cell parameter *c* could not be refined and was fixed to an arbitrary value of *c* = 7.1 Å, representing the calculated value of the lowest energy unit cell model (Table S1†).

The absence of amine (*ν*_N–H_) stretching bands in the FT-IR spectrum and the presence of the imine bands (*ν*_C=N_) at 1623 cm^−1^ indicate condensation of the framework as previously described for the amorphous polymer (Fig. S6†). ^13^C cross-polarization magic angle spinning (CP-MAS) ssNMR spectra support this finding as well, showing a characteristic signal for the imine carbon at *δ* = 154.6 ppm (Fig. 2d and S36†). Additional signals in the aliphatic region were assigned with the aid of ^1^H/^13^C heteronuclear correlation (HETCOR) spectroscopy (Fig. S37†) and correspond to the spacer (*δ* = 46.5, 26.6 ppm) and the motor unit (*δ* = 41.9, 26.6, 17.8 ppm) incorporated into the framework. ^13^C direct-excitation (DE) experiments (Fig. S38†) were carried out to obtain quantitative information about the motor/spacer ratio in the material. The signals at *δ* = 26.6 (spacer), and 17.8 ppm (motor) in the ^13^C-DE spectrum were found to be sufficiently resolved (Fig. 2d) and reveal a motor content of approximately 20% relative to spacer units (1.2 motors per pore in two layers). This value matches well with the theoretical content of 20%, indicating quantitative incorporation of the motor building block into the COF (relative to the starting ratio). Scanning electron microscopy (SEM) images show spherical, intergrown particles sized between 150 and 300 nm in diameter (Fig. S51†) with uniform crystallinity and visible pore channels along [00*l*] as evident from transmission electron microscopy (TEM) analysis (Fig. S52†). The hexagonal pattern of the fast Fourier transformed TEM image (Fig. 2b) further corroborates the symmetry and unit cell parameters obtained from Pawley refinement of the XRPD data. We performed N_2_ adsorption experiments to gain insights into the porosity of m_20_-COF. From the adsorption isotherms a BET surface area of *S*_BET_ = 604 m^2^ g^−1^ (0.45 cm^3^ g^−1^ total pore volume) and an average pore size centered at 2.7 nm was observed, derived by the QSDFT model for cylindrical pores (Fig. S42, S47 and S49†). Interestingly, the value found for the average pore size is rather small and would be expected to be at least 3 nm for an idealized eclipsed stacking, compared to COFs with similar topology and lattice parameters.^70^ Recent reports showed weak interlayer interactions to cause a decrease in apparent pore diameter in similar COFs,^71,72^ due to a poor registry of the layers in the stacking direction.^73^ As this property also depends on the orientation of the building blocks in neighboring layers, we modeled different orientations of the amine building blocks in the framework and additionally assessed the local structure with the help of pair distribution function (PDF) analysis.^74^

**Fig. 3:**
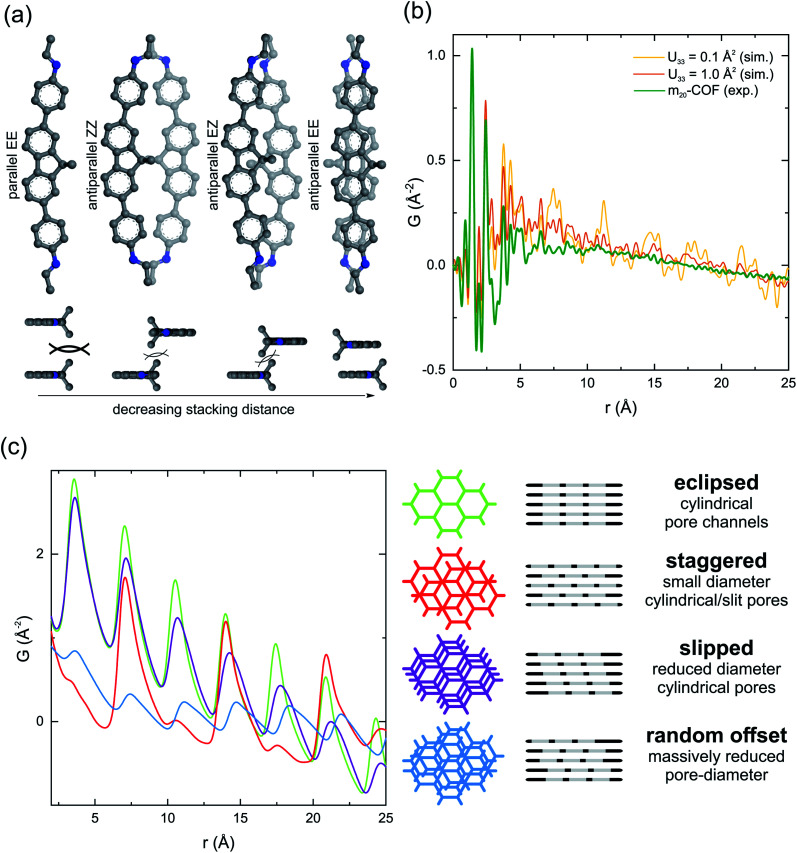
(a) Schematic illustration of molecular models probing the relative orientation and interlayer distance of the spacer moiety with respect to the imine bond configuration in a condensed framework. The antiparallel EE model combines the least steric repulsion of the methyl groups with best π overlap. (b) Experimental pair distribution function (PDF) for m_20_-COF and simulated PDFs with different values of the displacement parameter *U*_33_, simulating the effects of stacking and conformational disorder, are overlaid. (c) Interlayer density distributions simulated for different layer offset scenarios in 2D honeycomb layered COFs are shown to demonstrate the specific impact of different relative stacking offsets on the PDF profile (*U*_11_ = *U*_22_ = 1.0 Å^2^; *U*_33_ = 0.05 Å^2^).

Since the large majority of the m_20_-COF backbone consists of the spacer building block, we chose to simplify structure modeling by omitting the motor moiety in the model (Fig. 3). A closer look at the molecular structure of the spacer building block (Fig. 2a) reveals two characteristics of the amine building-blocks that can influence the layer stacking: the curvature of the linker and two benzylic methyl groups of the dimethylfluorene core, oriented perpendicularly to the aromatic system. These factors have a profound effect on the relative orientation of the building blocks in a condensed three-dimensional structure (Fig. S10†). The steric demand of the methyl groups (and in the case of motor 1 the rotor parts alike), pointing into the interlayer space, can complicate the structure's ability to lower its energy based on the optimization between π–π interactions and dispersion forces.^75,76^ This is particularly the case in structures with a parallel spacer orientation (Fig. 3a), rendering parallel models energetically unfavorable.^77^ With an antiparallel orientation of the unit, a regular conformation of the imine linkages is equally needed to construct evenly shaped pore channels, but due to the curvature of the fluorene unit, the imine conformation also determines the possibility for sufficient stacking interactions across the layers.^78–81^ This constraint led to the development of three possible unit cell structure models, which differ mainly in their imine configuration (Fig. S11†). A comparison of the total energies, obtained from Forcite geometry- and cell optimization in *Material Studio*, revealed the most favorable model with antiparallel stacking of the fluorene core and synchronized imine bonds pointing away from the dimethyl group (AA-1_EE, Table S1†) combining the least interlayer steric repulsion with best stacking interactions.

We employed PDF analysis on X-ray total scattering synchrotron data (Fig. 3b and S17†) to gain further insights into the local and intermediate length scale structure of the framework. The experimental PDF profile shows characteristic sharp peak profiles for atom-pair distances below *r* = 5 Å, provided by intralayer molecular connectivity, and a sloping baseline (>5 Å) indicative of intralayer pore–pore correlations. Unlike other 2D COFs, interlayer correlations are not observed, indicating the relationship between the stacked, and even neighboring layers is not coherent, due to a broadened distribution of interatomic distances between neighboring layers.^73,82^ Hence, a good qualitative agreement to the simulated pair distribution functions (AA-1_EE model) is obtained by implementing large atomic displacement parameters in the [001] direction (*U*_33_), simulating different levels of reduction in interlayer coherence (Fig. 3b). This agreement is not noticeably impacted if the motor unit is considered in the structural model, indicating that the motor units cannot be distinctly resolved without long-range ordering, since the bond distances in the motor units are too similar to the backbone of the material (Fig. S16†).

Simple models have been developed to demonstrate how the interlayer correlations are modified to understand the absence of any stacking reflection (00*l*) and associated correlations of interlayer density.^83^ These are illustrated for the associated layered honeycomb structures in Fig. 3c. In these models, large atomic displacement parameters (*U*_11_, *U*_22_) have been applied to wash out specific atom-pair correlations, resulting in effectively continuous density honeycombs. A shift from eclipsed to slipped stacking, for example, results in a broadening and effective shift of the interlayer density distribution (the distance of closest approach between neighboring branches does not change, but the maximum distance increases), while a staggered relationship decreases the amount of nearest neighbor correlations but maintains the second neighbor correlations still sitting in an eclipsed position. With completely randomized layer offsets, the intensity of the interlayer peaks for the model are substantially diminished, but still present due to the remaining equidistant spacing and thus cannot explain the absence of interlayer correlations as experimentally observed for m_20_-COF. However, in the presented material conformational distortions are expected to be coupled with offsets, thus allowing the atoms in the layers to also move off their ideal positions in the stacking direction, resulting in a distribution of different interlayer distances. The combination of both offsets and conformational flexibility can lead to the extinction of the interlayer coherence, and no modulation in stacking density remains. This is further supported by simulated diffraction patterns for m_20_-COF as a function of randomized layer offset magnitude (lateral) and interlayer displacement parameter using DIFFaX^84^ (Fig. S53†). This is in contrast to continuous layered materials like graphite, because interlayer offsets lead to a wider distribution of possible interlayer interaction environments due to the intrinsic porosity in the layers. In certain cases, with drastic offsets and additional factors, *e.g.* flexibility, causing out-of-plane distortions of the layers, this effect can lead to a practically X-ray amorphous, non-porous structure.^82^ However, in the case of m_20_-COF, interlayer offsets and conformational distortions only decrease the apparent pore-diameter, while retaining an overall porous framework, as probed by N_2_ sorption experiments.

Additional materials with 0%, 5%, and 10% motor-content (m_0/5/10_-COF) were prepared under identical synthetic conditions as spectroscopic reference materials for COFs without motor-units (m_0_-COF) and with reduced pore-occupancy. Notably, these materials showed essentially identical XRPD patterns and FT-IR spectra to m_20_-COF (Fig. S7 and S14†). Due to the absence of motor units, m_0_-COF exhibited a slightly increased BET surface area and pore volume of 938 m^2^ g^−1^ and 0.63 cm^3^ g^−1^, respectively, and pore diameters of 2.9 nm *vs.* 2.7 nm in m_20_-COF (Fig. S47–50†).

This comprehensive analysis suggests that additional functional groups (methyl or motor) drastically impact the formation of ordered layered COF materials. Nevertheless, the residual porosity and pore channels may in principle allow for movement of the rotor units and potential intra-framework dynamic processes.

### Irradiation of solid-state materials

*In situ* Raman spectroscopy of the dried powdered materials under irradiation was carried out to investigate light-driven dynamics of the molecular motors in the polymers and COFs. In previous reports on metal–organic frameworks, this technique enabled the detection of light-stimulated formation of the metastable isomer as well as the thermal relaxation of the metastable state of the motor after photoexcitation.^8,11^ With a half-life of 10.3 min for the imine functionalized motor 2, the isomerization process is expected to be observable at ambient temperature as confirmed by the switching of the motor 2 in toluene (Fig. 1d).

**Fig. 4:**
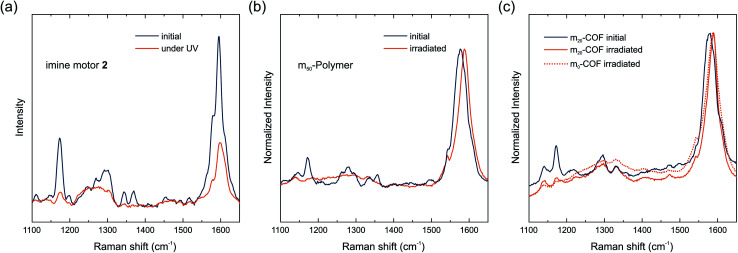
Raman spectra of the imine motor 2 as a thin-film (a), m_50_-P (b), and m_20_-COF. The thin-film of the molecular imine motor 2 was recorded at 785 nm and irradiated at 365 nm with a UV-LED. Raman spectra of m_50_-P (b), m_20_-COF and m_0_-COF (c) were recorded with a Raman laser at 355 nm for simultaneous excitation of the motor.

To identify motor-related signals in the Raman spectra, polymers were initially measured at 785 nm (Fig. S8†). Primary signals at 1590, 1305, 1153 and 1178 cm^−1^ for all polymers were identified. The motor containing polymers m_50_-P and m_100_-P additionally show weak bands at 1517, 1370, 1268 and 1115 cm^−1^, attributed to the molecular motor.^8^ Due to the absorption of the motors, Raman measurements at 355 nm allowed for the simultaneous Raman scattering and excitation of the motor within the materials with a more intense light source (Fig. 4b and c). Under these conditions, however, an irreversible decrease in signal strength and signal broadening occurred, identical to the behavior of a thin-film of the molecular imine functionalized motor 2 irradiated with a 365 nm LED (Fig. 4a). This irreversible change of the spectrum caused a total loss of signals assigned to the motor moiety and thus prevents a detailed study of the rotary behavior of the motor in the material.

**Fig. 5:**
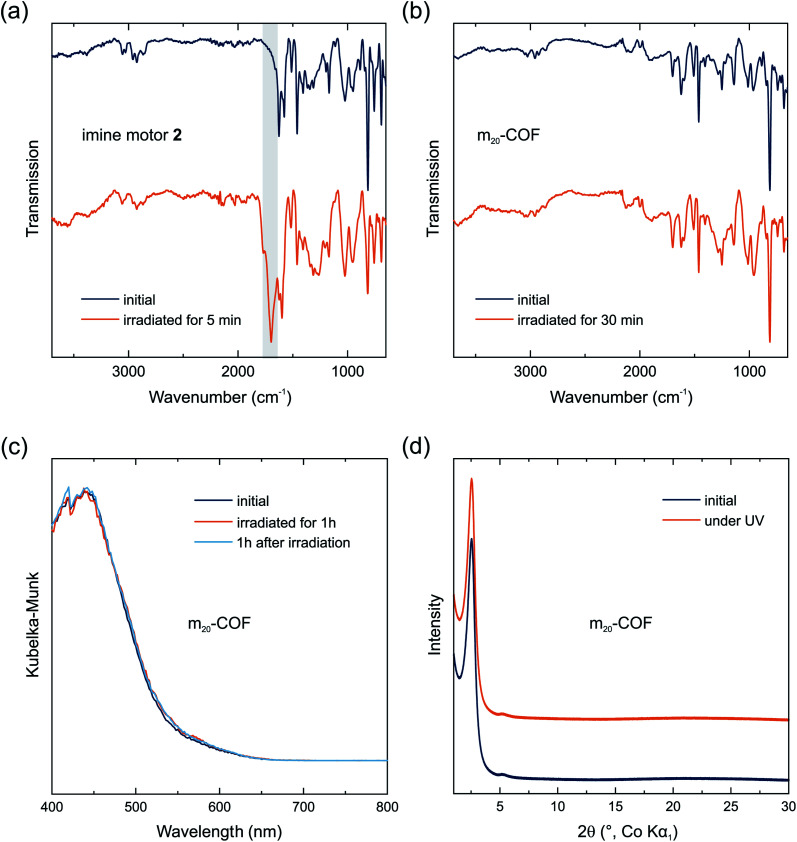
FT-IR spectra of a thin film of the imine motor 2 (a) and m_20_-COF (b) under prolonged irradiation at 365 nm (UV-LED). Grey area highlights appearing C=O vibrations during irradiation of imine motor 2. Direct reflectance UV/vis spectra (c) and XRPD patterns (d) of m_20_-COF before and during irradiation (>1.5 h) at 365 nm are shown. The patterns are stacked with an arbitrary offset to aid visibility.

Raman spectra at 785 nm of the COF samples show similar signals as the polymers (Fig. S9†). The spectra of all COF samples are almost identical irrespective of the amount of motor integrated in the material. Signals referring to the motors cannot be identified likely because the motor concentration in the materials is below the limit of detection. Nonetheless, a spectrum of m_20_-COF was recorded at 355 nm and again a loss in signal intensity as well as broadening was observed during prolonged irradiation (Fig. 4c). A comparison of the Raman spectra of m_0_-COF and m_20_-COF under irradiation shows that this observation is independent of the motor content, and occurs similarly with the amorphous polymers. To understand the unexpected intensity losses during the Raman measurements, we conducted additional experiments to probe changes in the chemical and long-range order. FT-IR spectra of the imine motor 2 thin film and m_20_-COF (Fig. 5a and b) show drastic changes for the molecular thin film 2 already after 5 min of irradiation with a UV-LED at 365 nm, whereas the spectrum for m_20_-COF remained essentially unchanged even for prolonged irradiation (Fig. 5b). The appearance of an intense, irreversible signal centered at 1697 cm^−1^, attributed to an aldehyde C=O vibration, shows that the molecular imine motor 2 readily hydrolyzed under irradiation of a desolvated thin film. On the other hand, this is not observed for the porous framework, highlighting an increased hydrolytic stability of the imine if incorporated in the condensed COF-network.

Similar to the FT-IR spectra, UV/vis absorption spectra and XRPD patterns of m_20_-COF (Fig. 5c and d) do not indicate any long-range structural changes or decomposition of the material even under irradiation at 365 nm with an LED for several hours. We thus attribute the observed intensity loss during Raman measurements to local heating effects under high intensity, focused light irradiation of the Raman laser. However, changes in the local structure, such as interlayer slipping/layer bending, or even local decomposition, perhaps driven or enhanced by local heating effects, may also occur and remain invisible to the available analytical techniques. Whereas these data clearly demonstrate the increased stability of the molecular building blocks in the condensed COF materials under irradiation, they also highlight that the applied spectroscopic and diffraction techniques are not sensitive to monitor motor excitation in the solid state at low motor loadings and consequently low fraction of the metastable state, emphasizing the need for local structural probes, such as PDF, to pinpoint the effects of interlayer shifting or strain formation on rotor dynamics.

## Conclusion

Herein we present the first example of a molecular motor integrated within the backbone of an imine-based polymer and mesoporous COF using a novel diamine light-driven molecular motor. We detail synthesis strategies to enhance crystallinity by using an acid catalyst under solvothermal conditions and demonstrate that the motors are preserved under such harsh reaction conditions. Using a wide range of methods such as FT-IR, NMR, SEM, TEM, XRPD, PDF, DFT and nitrogen adsorption, we detail the composition, porosity, molecular, and framework structure of a series of motorized polymers and COFs. By this, we are able to identify the long-range and local structure of the motor-containing COF, building the basis for future investigations of such machine containing solids. As such, the materials described in this work can act as model compounds from which we identified two major challenges with respect to integration, operation and observation of light-driven molecular motors and photo-responsive dynamics in 2D layered covalent organic frameworks: (i) sensitivity of analytical techniques towards low concentration of light-responsive functionality, and (ii) local and long-range structural disorder induced by the interaction of adjacent layers.

In a 2D COF, curved building blocks such as motor 1 and spacer 4 affect the self-assembled structure drastically. The curvature results in different conformational combinations, which reduce the average periodicity of the stacked layers. The conformation of the imine linkage as well as the steric demand of perpendicularly oriented methyl groups or rotor parts in spacer 4 and motor 1, respectively affect the stacking additionally. This has detrimental influence on interlayer stacking correlations and the available pore volume as well as the local environment of the motor. Yet, the accessible pore size is still found to exceed the radius of motor rotation. However, intermolecular interactions from adjacent layers may hamper or restrict motor movement even if commonly applied benchmarks for accessible void indicate otherwise. We thus conclude that local structural characteristics, such as interlayer interactions and stacking offset, which are invisible to most employed analytical methods, need to be considered for the development of analytical tools and the material's design to establish and investigate unrestricted motor dynamics in porous layered solids. Integrating molecular motors into three-dimensional COFs could circumvent this challenge. These materials are however challenging to synthesize and are prone to form interpenetrated structures, which can again constrain the available free volume for motor rotation.^85^

Beyond these structural influences and design criteria, the commonly available and applied analytical techniques including solid state NMR, FT-IR, and Raman spectroscopy did not allow us to draw a definite conclusion on the rotation ability of the motor in the solid polymer and COF materials. In the presented COF materials the motor signals could not be detected due to the low concentration in the solid. An increased motor content, however, concomitantly reduces the available pore void and thus interferes with the free rotation of the motor units, as demonstrated with the motor-containing polymer (m_100_-P). Our study thus highlights that new analytical techniques with high local sensitivity and *in situ* operation have to be developed to study the behavior of motor dynamics in this class of porous materials further. Advanced *in situ* ssNMR techniques under irradiation with abundant heteroatoms like fluorine and also ^13^C enriched motor units may enable to observe the rotation of embedded motors in these materials – if considered with structural characteristics, *e.g.* interlayer interactions and stacking offset, arising from the stacked layers. We envision that such analytical tools will also allow studies of interlayer dynamics and dynamic host–guest properties caused by the light-responsive dynamics of framework-embedded molecular motors. The synthetic procedures and in-depth structural characterization demonstrated herein provide access to these materials as model compounds and structural model systems. Furthermore, these can be used for in depth molecular dynamics simulations to probe design criteria for the operation of light-driven molecular motors in porous solids.

## Data availability

Further data are stored on the DaRUS data repository (https://darus.uni-stuttgart.de/) and are accessible upon request.

## Author contributions

C. S. and L. G. contributed equally to this work. C. S. synthesized and analyzed the light-responsive molecular motors, imines, amines and derived polymers. L. G. synthesized and characterized the COF materials and established their structural models. M. W. T. and M. E. performed PDF analysis. W. R. B. performed Raman analysis. D. D. performed DFT analysis of the molecular motors. M. K. supported synthesis and spectroscopic analysis of the molecular motors. B. V. L., B. L. F. and S. K. supervised the project and supported writing of the manuscript. All Authors read and commented on the manuscript.

## Conflicts of interest

The authors declare no competing financial interests.

## Supplementary Material

SC-013-D2SC02282F-s001

## References

[cit1] Browne W. R., Feringa B. L. (2006). Making molecular machines work. Nat. Nanotechnol..

[cit2] Feringa B. L. (2017). The Art of Building Small: From Molecular Switches to Motors (Nobel Lecture). Angew. Chem., Int. Ed..

[cit3] Kassem S. (2017). *et al.* Artificial molecular motors. Chem. Soc. Rev..

[cit4] Iino R., Kinbara K., Bryant Z. (2020). Introduction: Molecular Motors. Chem. Rev..

[cit5] Pooler D. R. S., Lubbe A. S., Crespi S., Feringa B. L. (2021). Designing light-driven rotary molecular motors. Chem. Sci..

[cit6] Kathan M. (2022). *et al.* A light-fuelled nanoratchet shifts a coupled chemical equilibrium. Nat. Nanotechnol..

[cit7] Li Q. (2015). *et al.* Macroscopic contraction of a gel induced by the integrated motion of light-driven molecular motors. Nat. Nanotechnol..

[cit8] Danowski W. (2019). *et al.* Unidirectional rotary motion in a metal-organic framework. Nat. Nanotechnol..

[cit9] Moulin E., Faour L., Carmona-Vargas C. C., Giuseppone N. (2020). From Molecular Machines to Stimuli-Responsive Materials. Adv. Mater..

[cit10] Corra S. (2020). *et al.* Photoactivated Artificial Molecular Machines that Can Perform Tasks. Adv. Mater..

[cit11] Danowski W. (2020). *et al.* Visible-Light-Driven Rotation of Molecular Motors in a Dual-Function Metal-Organic Framework Enabled by Energy Transfer. J. Am. Chem. Soc..

[cit12] Coskun A. (2012). *et al.* Great expectations: can artificial molecular machines deliver on their promise?. Chem. Soc. Rev..

[cit13] Baroncini M. (2018). *et al.* Making and Operating Molecular Machines: A Multidisciplinary Challenge. ChemistryOpen.

[cit14] Dattler D. (2020). *et al.* Design of Collective Motions from Synthetic Molecular Switches, Rotors, and Motors. Chem. Rev..

[cit15] Krause S., Feringa B. L. (2020). Towards artificial molecular factories from framework-embedded molecular machines. Nat. Rev. Chem..

[cit16] Grommet A. B., Lee L. M., Klajn R. (2020). Molecular Photoswitching in Confined Spaces. Acc. Chem. Res..

[cit17] Feng L. (2021). *et al.* Active mechanisorption driven by pumping cassettes. Science.

[cit18] Payer D. (2007). *et al.* Toward mechanical switching of surface-adsorbed [2]catenane by *in situ* copper complexation. J. Am. Chem. Soc..

[cit19] Lu T., Zhang L., Gokel G. W., Kaifer A. E. (2002). The first surface-attached catenane: self-assembly of a two-component monolayer. J. Am. Chem. Soc..

[cit20] Norgaard K. (2005). *et al.* Structural evidence of mechanical shuttling in condensed monolayers of bistable rotaxane molecules. Angew. Chem., Int. Ed..

[cit21] Flood A. H. (2004). *et al.* The role of physical environment on molecular electromechanical switching. Chem.–Eur. J..

[cit22] Foy J. T. (2017). *et al.* Dual-light control of nanomachines that integrate motor and modulator subunits. Nat. Nanotechnol..

[cit23] van Delden R. A. (2005). *et al.* Unidirectional molecular motor on a gold surface. Nature.

[cit24] Pollard M. M. (2008). *et al.* Light-driven rotary molecular motors on gold nanoparticles. Chem.–Eur. J..

[cit25] Eelkema R. (2006). *et al.* Rotational reorganization of doped cholesteric liquid crystalline films. J. Am. Chem. Soc..

[cit26] Eelkema R. (2006). *et al.* Molecular machines: nanomotor rotates microscale objects. Nature.

[cit27] Castiglioni F. (2020). *et al.* Modulation of porosity in a solid material enabled by bulk photoisomerization of an overcrowded alkene. Nat. Chem..

[cit28] Martinez-Bulit P., Stirk A. J., Rotors L. S. J. (2019). Motors, and Machines Inside Metal–Organic Frameworks. Trends Chem..

[cit29] Krause S., Hosono N., Kitagawa S. (2020). Chemistry of Soft Porous Crystals: Structural Dynamics and Gas Adsorption Properties. Angew. Chem., Int. Ed..

[cit30] Kolodzeiski E., Amirjalayer S. (2021). Collective structural properties of embedded molecular motors in functionalized metal-organic frameworks. Phys. Chem. Chem. Phys..

[cit31] Evans J. D., Krause S., Feringa B. L. (2021). Cooperative and synchronized rotation in motorized porous frameworks: impact on local and global transport properties of confined fluids. Faraday Discuss..

[cit32] Cote A. P. (2005). *et al.* Porous, crystalline, covalent organic frameworks. Science.

[cit33] Stegbauer L., Schwinghammer K., Lotsch B. V. (2014). A hydrazone-based covalent organic framework for photocatalytic hydrogen production. Chem. Sci..

[cit34] Wei P. F. (2018). *et al.* Benzoxazole-Linked Ultrastable Covalent Organic Frameworks for Photocatalysis. J. Am. Chem. Soc..

[cit35] Liu X. (2019). *et al.* Recent advances in covalent organic frameworks (COFs) as a smart sensing material. Chem. Soc. Rev..

[cit36] Nguyen H. L. (2020). *et al.* A Porous Covalent Organic Framework with Voided Square Grid Topology for Atmospheric Water Harvesting. J. Am. Chem. Soc..

[cit37] Zhao X., Pachfule P., Thomas A. (2021). Covalent organic frameworks (COFs) for electrochemical applications. Chem. Soc. Rev..

[cit38] Keller N., Bein T. (2021). Optoelectronic processes in covalent organic frameworks. Chem. Soc. Rev..

[cit39] Liu R. (2021). *et al.* Covalent organic frameworks: an ideal platform for designing ordered materials and advanced applications. Chem. Soc. Rev..

[cit40] Zhu Y., Zhang W. (2014). Reversible tuning of pore size and CO2 adsorption in azobenzene functionalized porous organic polymers. Chem. Sci..

[cit41] Zhang J. (2014). *et al.* A novel azobenzene covalent organic framework. CrystEngComm.

[cit42] Das G. (2019). *et al.* Azobenzene-Equipped Covalent Organic Framework: Light-Operated Reservoir. J. Am. Chem. Soc..

[cit43] Sun N. (2022). *et al.* Photoresponsive Covalent Organic Frameworks with Diarylethene Switch for Tunable Singlet Oxygen Generation. Chem. Mater..

[cit44] Das G. (2019). *et al.* A polyrotaxanated covalent organic network based on viologen and cucurbit[7]uril. Commun. Chem..

[cit45] Ruan X. (2021). *et al.* Mechanical Bond Approach to Introducing Self-Adaptive Active Sites in Covalent Organic Frameworks for Zinc-Catalyzed Organophosphorus Degradation. ACS Cent. Sci..

[cit46] Haase F., Lotsch B. V. (2020). Solving the COF trilemma: towards crystalline, stable and functional covalent organic frameworks. Chem. Soc. Rev..

[cit47] Feng L. (2020). *et al.* Destruction of Metal-Organic Frameworks: Positive and Negative Aspects of Stability and Lability. Chem. Rev..

[cit48] Rauche M. (2019). *et al.* New insights into solvent-induced structural changes of (13)C labelled metal-organic frameworks by solid state NMR. Chem. Commun..

[cit49] Bessinger D. (2021). *et al.* Fast-Switching Vis-IR Electrochromic Covalent Organic Frameworks. J. Am. Chem. Soc..

[cit50] Dey K. (2021). *et al.* Self-Assembly-Driven Nanomechanics in Porous Covalent Organic Framework Thin Films. J. Am. Chem. Soc..

[cit51] Chen J. (2018). *et al.* Artificial muscle-like function from hierarchical supramolecular assembly of photoresponsive molecular motors. Nat. Chem..

[cit52] Koumura N. (1999). *et al.* Light-driven monodirectional molecular rotor. Nature.

[cit53] Koumura N. (2002). *et al.* Second generation light-driven molecular motors. Unidirectional rotation controlled by a single stereogenic center with near-perfect photoequilibria and acceleration of the speed of rotation by structural modification. J. Am. Chem. Soc..

[cit54] Vicario J., Meetsma A., Feringa B. L. (2005). Controlling the speed of rotation in molecular motors. Dramatic acceleration of the rotary motion by structural modification. Chem. Commun..

[cit55] Lehn J. M. (2007). From supramolecular chemistry towards constitutional dynamic chemistry and adaptive chemistry. Chem. Soc. Rev..

[cit56] Belowich M. E., Stoddart J. F. (2012). Dynamic imine chemistry. Chem. Soc. Rev..

[cit57] Li Y. (2020). *et al.* New synthetic strategies toward covalent organic frameworks. Chem. Soc. Rev..

[cit58] Lohse M. S., Bein T. (2018). Covalent Organic Frameworks: Structures, Synthesis, and Applications. Adv. Funct. Mater..

[cit59] Mokhtari N., Afshari M., Dinari M. (2020). Synthesis and characterization of a novel fluorene-based covalent triazine framework as a chemical adsorbent for highly efficient dye removal. Polymer.

[cit60] Wang L. (2017). *et al.* Fluorene-Based Two-Dimensional Covalent Organic Framework with Thermoelectric Properties through Doping. ACS Appl. Mater. Interfaces.

[cit61] Ma T. (2018). *et al.* Observation of Interpenetration Isomerism in Covalent Organic Frameworks. J. Am. Chem. Soc..

[cit62] Li R. L. (2021). *et al.* Two-Dimensional Covalent Organic Framework Solid Solutions. J. Am. Chem. Soc..

[cit63] Xue Y.-J. (2020). *et al.* Isomeric effect of fluorene-based fused-ring electron acceptors to achieve high-efficiency organic solar cells. J. Mater. Chem. A.

[cit64] Chen X., Fu X., Qiu Y., Yuan J. (2010). 2,7-Dibromo-9,9-dimethyl-9H-fluorene. Acta Crystallogr..

[cit65] Durka K., Kazimierczuk K., Luliński S. (2022). Dipole-dipole interactions of sulfone groups as a tool for self-assembly of a 2D Covalent Organic Framework derived from a non-linear diboronic acid. Microporous Mesoporous Mater..

[cit66] Conyard J. (2012). *et al.* Ultrafast dynamics in the power stroke of a molecular rotary motor. Nat. Chem..

[cit67] Giuseppone N., Schmitt J. L., Schwartz E., Lehn J. M. (2005). Scandium(III) catalysis of transimination reactions. Independent and constitutionally coupled reversible processes. J. Am. Chem. Soc..

[cit68] Matsumoto M. (2017). *et al.* Rapid, Low Temperature Formation of Imine-Linked Covalent Organic Frameworks Catalyzed by Metal Triflates. J. Am. Chem. Soc..

[cit69] Emmerling S. T. (2021). *et al. In situ* monitoring of mechanochemical covalent organic framework formation reveals templating effect of liquid additive. Chem.

[cit70] Evans A. M. (2021). *et al.* Trends in the thermal stability of two-dimensional covalent organic frameworks. Faraday Discuss..

[cit71] Emmerling S. T. (2021). *et al.* Interlayer Interactions as Design Tool for Large-Pore COFs. J. Am. Chem. Soc..

[cit72] Xu H., Gao J., Jiang D. (2015). Stable, crystalline, porous, covalent organic frameworks as a platform for chiral organocatalysts. Nat. Chem..

[cit73] Pütz A. M. (2020). *et al.* Total scattering reveals the hidden stacking disorder in a 2D covalent organic framework. Chem. Sci..

[cit74] Terban M. W., Billinge S. J. L. (2022). Structural Analysis of Molecular Materials Using the Pair Distribution Function. Chem. Rev..

[cit75] Koo B. T., Dichtel W. R., Clancy P. (2012). A classification scheme for the stacking of two-dimensional boronate ester-linked covalent organic frameworks. J. Mater. Chem..

[cit76] Fan Y. (2017). *et al.* A Case Study on the Influence of Substitutes on Interlayer Stacking of 2D Covalent Organic Frameworks. Chemistry.

[cit77] Martinez-Abadia M., Mateo-Alonso A. (2020). Structural Approaches to Control Interlayer Interactions in 2D Covalent Organic Frameworks. Adv. Mater..

[cit78] Albacete P. (2018). *et al.* Layer-Stacking-Driven Fluorescence in a Two-Dimensional Imine-Linked Covalent Organic Framework. J. Am. Chem. Soc..

[cit79] Yin H. Q., Yin F., Yin X. B. (2019). Strong dual emission in covalent organic frameworks induced by ESIPT. Chem. Sci..

[cit80] Haase F. (2017). *et al.* Tuning the stacking behaviour of a 2D covalent organic framework through non-covalent interactions. Mater. Chem. Front..

[cit81] Xun S., Li H., Sini G., Bredas J. L. (2021). Impact of Imine Bond Orientations on the Geometric and Electronic Structures of Imine-based Covalent Organic Frameworks. Chem.–Asian J..

[cit82] Grunenberg L. (2021). *et al.* Amine-Linked Covalent Organic Frameworks as a Platform for Postsynthetic Structure Interconversion and Pore-Wall Modification. J. Am. Chem. Soc..

[cit83] Warren B. E. (1941). X-Ray Diffraction in Random Layer Lattices. Phys. Rev..

[cit84] Treacy M. M. J., Newsam J. M., Deem M. W. (1997). A general recursion method for calculating diffracted intensities from crystals containing planar faults. Proc. R. Soc. London, Ser. A.

[cit85] Gao C. (2020). *et al.* Redox-triggered switching in three-dimensional covalent organic frameworks. Nat. Commun..

