# Supplementary material for: Light-driven molecular motors embedded in covalent organic frameworks
Source: Chem Sci. 2022 Jun 2;13(28):8253–64. doi: 10.1039/d2sc02282f (PMC9297439; doi:10.1039/d2sc02282f)
Supplement: SC-013-D2SC02282F-s001 [file SC-013-D2SC02282F-s001.pdf]

Electronic Supplementary Material (ESI) for Chemical Science.
This journal is © The Royal Society of Chemistry 2022

Supporting Information:

Light-Driven Molecular Motors Embedded in Covalent Organic Frameworks

Cosima Stähler,[†,a] Lars Grunenberg,[†,b,c] Maxwell W. Terban,[b] Wesley R. Browne,[a] Daniel Doellerer,[a] Michael Kathan,[a] Martin Etter,[d] Bettina V. Lotsch,[b,c,e,*] Ben L. Feringa,[a,*] Simon Krause[b,*]

[†] The authors contributed equally.

[a] Stratingh Institute for Chemistry, Rijksuniversiteit Groningen, Nijenborgh 4, 9747 AG Groningen, Netherlands

[b] Max Planck Institute for Solid State Research, Heisenbergstr. 1, 70569 Stuttgart, Germany

[c] Department of Chemistry, Ludwig-Maximilians-Universität (LMU), Butenandtstr. 5-13, 81377 Munich, Germany

[d] Deutsches Elektronen-Synchrotron (DESY), Notkestr. 85, 22607 Hamburg, Germany

[e] E-conversion, Lichtenbergstrasse 4a, 85748 Garching, Germany

[*] Email: b.lotsch@fkf.mpg.de, b.l.feringa@rug.nl, s.krause@fkf.mpg.de

Table of Contents

Methods and Equipment 3
Synthetic Procedures 6
Building Block Synthesis 6
Polymer Synthesis 9
COF Synthesis 10
Analytical Data 11
UV-Vis spectra 11
FT-IR Spectra 12
Raman Spectra 15
PXRD Data and Structure Modeling 16
Pair Distribution Function (PDF) Analysis 20
NMR Spectra 22
ssNMR Spectra 28
N_2 Sorption Analysis 34
SEM/TEM Analysis 41
DFT Calculations 42
DIFFaX Simulations 52
References 53

Methods and Equipment

General methods: All reactions, unless otherwise noted, were performed with magnetic stirring under inert gas (N_2 or Ar) atmosphere using standard Schlenk techniques. Reaction temperatures were electronically monitored as external heating block temperatures. Reagents were purchased from different commercial sources and used without further purification.

NMR spectroscopy: NMR spectra were obtained using a Varian Mercury Plus (^{1}H: 400 MHz, ^{13}C: 100 MHz) or a Bruker Avance NEO (^{1}H: 600 MHz, ^{13}C: 150 MHz). Chemical shifts are reported in δ units (ppm) relative to the residual solvent signal of $CDCl_3$ (^{1}H NMR, δ 7.26 ppm; ^{13}C NMR, δ 77.0 ppm) C_6D_6 (^{1}H NMR, 7.16 ppm; ^{13}C NMR 128.1 ppm) or DMSO-d_6 (^{1}H NMR, δ 2.50 ppm; ^{13}C NMR, δ 39.5 ppm). The splitting pattern of peaks is designated as follows: s (singlet), d (doublet), t (triplet), m (multiplet), br (broad), p (quintet) or dd (doublet of doublets).

NMR irradiation experiments were performed on a Varian Innova (^{1}H: 500 MHz) instrument at 10 °C with a Thorlab model M395F1 and M365F1 LED coupled to a 0.6 mm optical fiber, which sends the light into the NMR tube inside the spectrometer.

Solid state NMR spectra (ssNMR) were recorded at room temperature in 3.2 mm ZrO_2 rotors on a Bruker Neo 600 MHz spectrometer using Bruker BL3.2 triple resonance MAS probes. Standard instrument library pulse sequences were used. Chemical shifts were referenced relative to TMS (^{1}H and ^{13}C, 0.0 ppm) and CH_3NO_2 (^{15}N, 0.0 ppm).

High resolution mass spectrometry (HRMS): HRMS were recorded on an LTQ Orbitrap XL.

Infrared spectroscopy: IR spectra were recorded on a Perkin Elmer UATR Two FT-IR spectrometer equipped with an attenuated total reflection (ATR) measuring unit. IR data are reported in wavenumbers (cm^{-1}) of normalized absorption. The IR bands are characterized as w (weak), m (medium), s (strong), or br (broad). Irradiated IR samples: A small amount of dried COF powder was deposited on the sample holder. Irradiation was performed with a M365F1 LED by Thorlabs from a distance of a few centimeters for an optimal focus of the light on the sample. The IR spectra were measured in situ.

UV-Vis absorption spectroscopy: UV-Vis absorption spectra of solutions were recorded in a cm quartz cuvette with a Agilent Technologies Cary 8454 UV-Vis spectrometer, equipped with a Quantum Northwest temperature controller.

DR-UV/Vis: A small amount of dried COF powder was carefully mixed with $Ba(SO_4)_2$ and deposited in a sample holder. Irradiation was performed with a Throlabs M365F1 LED inside the spectrometer, without

moving the sample holder. Spectra were taken before and after irradiation and after letting the sample stand in the dark for 1 h.

Raman spectroscopy: Raman spectra were recorded either using a Raman microscope with optical head from Perkin Elmer with excitation at 785 nm (Ondax, 5-50 mW at sample) typically with a 50x long working distance objective or a Inphotonics low-cost Raman probe with Raman scattering feed to a Shamrock163i spectrograph with a 600 l/mm 750 nm blazed grating and a SMA fiber coupler and correction lens with spectra collected using an Andor Technology iVac-316-LDC-DD or iDus-420-OE CCD, respectively. Spectra were calibrated with polystyrene or cyclohexane (ASTM E 1840). Spectra were typically 0.5 to 1 s acquisitions and 60 accumulations. Raman spectra at 355 nm (10 mW, Cobolot Lasers Zouk) were recorded in 180° backscattering mode with 7.5 mm focal length collection lens at sample and 15 cm focal length lens feeding light into the Shamrock500i (ANDOR technology) spectrograph equipped with a iDUS-420-BU2 CCD detector and 1800 l/mm grating blazed at 300 nm. A Semrock long pass filter was used to reject Rayleigh scattering. Spectra were obtained with ANDOR Solis, processed in Spectragryph 1.2.14 (F. Menges).

Gas sorption measurements: Sorption measurements for COFs were performed on a Quantachrome Instruments Autosorb iQ MP with Nitrogen at 77 K. The samples were degassed for 12 h at 120 °C under vacuum prior to the gas adsorption studies. Pore size distribution was determined from Nitrogen adsorption isotherms using the QSDFT cylindrical pores in carbon model for nitrogen at 77 K. For multipoint BET surface area calculations, pressure ranges were chosen with the help of the BET assistant in the ASiQwin software, which chooses BET tags in accordance with the ISO recommendations equal or below the maximum in grams per square meter.

Nitrogen physisorption at 77 K of the polymers was performed on a Micromeritics® ASAP 2420 instruments using the instruments default settings. Previous to the physisorption experiments samples were degassed at 80 °C for 6 h.

X-ray powder diffraction (XRPD): X-ray powder diffraction experiments were performed on a Stoe Stadi P diffractometer (Cu-Kα_1 or Co- Kα_1, Ge(111)) in Debye-Scherrer geometry. The samples were measured in sealed glass capillaries (OD = 0.7 mm) and spun for improved particle statistics. A Thorlabs M365LP1-C4 LED light source, equipped with a Thorlabs COP4-A Zeiss lens, was used for irradiated XRPD experiments.

Pawley refinements: Pawley refinements were performed using TOPAS v6. The background was corrected with Chebychev polynomials (Order 10). Simple axial and zero-error corrections were used together with additional corrections for Lorentzian crystallite size broadening.

Pair distribution function (PDF) analysis: Total scattering measurements were carried out using the high energy Powder Diffraction and Total Scattering Beamline P02.1 of PETRA III at the Deutsches Elektronen-Synchrotron (DESY). X-ray total scattering data were collected in rapid acquisition mode (RAPDF).[1] A large-area 2D Varex XRD 4343CT detector (2880×2880 pixels, 150×150 μm^2 each) was used at a sample-to-detector distance of approximately 500 mm. Samples were loaded into 1.0 mm ID/1.1 mm OD polyimide capillaries (Cole-Parmer) and measured at room temperature. The incident energy of the X-rays was 59.792 keV (λ = 0.20736 Å). A measurement of LaB_6 was collected at room temperature as a standard for calibration of the setup. Calibration was performed, and the raw 2D intensity was corrected for polarization and azimuthally integrated and converted to 1D intensity versus Q ($Q = 4\pi \sin \theta/\lambda$ is the magnitude of the scattering momentum transfer, with 2θ scattering angle) using pyFAI[2] with the xpdtools package[3].

Further correction and normalization of the integrated 1D diffraction intensities were carried out to obtain the total scattering structure function, F(Q), which was Fourier transformed to obtain the pair distribution function (PDF), G(r) using PDFgetX3[4] within xPDFsuite[5]. The maximum value used in the Fourier transform of the total scattering data (Qmax) was 15.0 $Å^{-1}$. Simulated PDFs were performed using PDFgui[5,6].

Supercritical CO_2 activation: Activation of the methanol-soaked COF samples with supercritical CO_2 was performed on a Leica EM CPD300 critical point dryer.

Solvent removal from the polymers was performed *via* supercritical CO_2 extraction using a Tousimis Autosamdri-815 Series B Critical Point Dryer. The sample were previously washed and suspended in ethanol.

Scanning electron microscopy (SEM): SEM SE (secondary electron) detector images were obtained on a Zeiss Merlin SEM.

Transmission electron microscopy (TEM): TEM analysis was performed with a Philips CM30 ST (300kV, LaB6 cathode). The samples were prepared dry onto a copper lacey carbon grid (Plano).

Synthetic Procedures

Building Block Synthesis

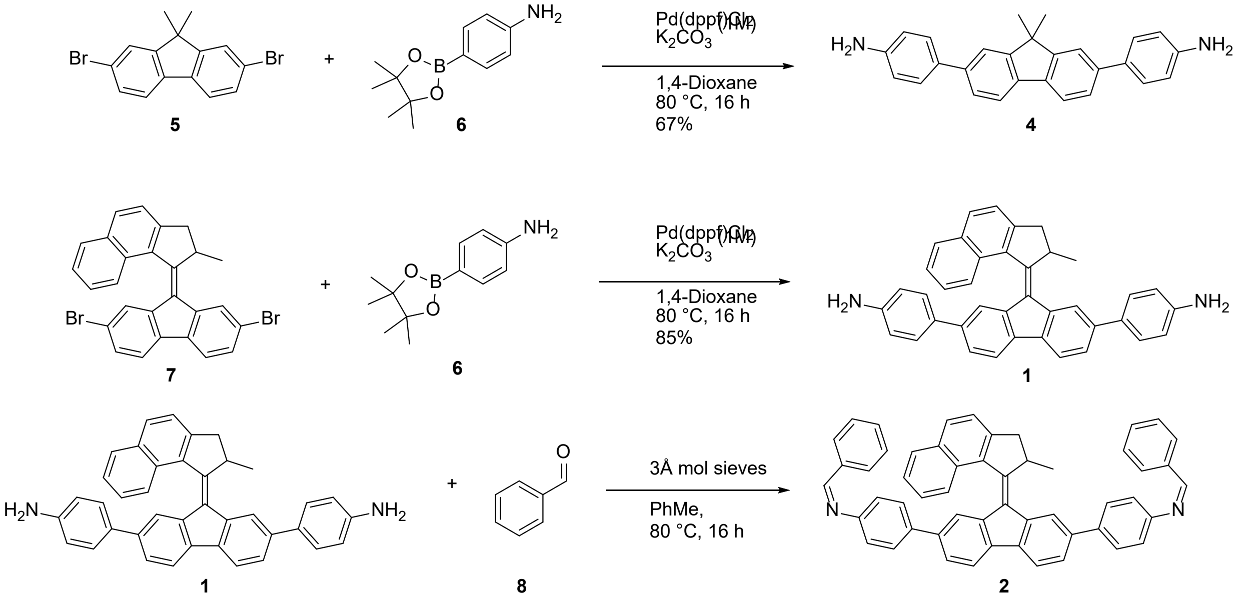

4,4'-(9-(2-Methyl-2,3-dihydro-1*H*-cyclopenta[*a*]naphthalen-1-ylidene)-9*H*-fluorene-2,7-diyl)dianiline (1)

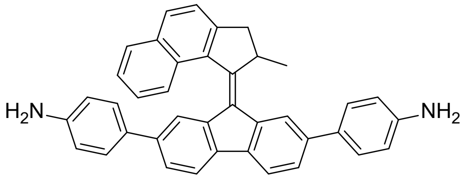

Motor **7** (300 mg, 597 µmol, 1.0 equiv.),[7] 4-aminophenylboronic pinacol ester (**6**, 393 mg, 1.79 mmol, 3.0 equiv.) and Pd(dppf)Cl_2 (44.0 mg, 60.0 µmol, 0.1 equiv.) were mixed with 10 mL 1,4-dioxane and 1M aqueous potassium carbonate solution (2.4 mL, 2.4 mmol, 4.0 equiv.). The mixture was degassed by applying vacuum until the solvent started bubbling and was saturated with N_2. This process was repeated three times. Subsequently the mixture was stirred at 80 °C for 17 h under N_2 atmosphere. The reaction mixture was cooled down to room temperature, ethyl acetate and water were added, the phases were separated and the aqueous phase was extracted with ethyl acetate (3 x 50 mL). The combined organic phase was washed with water (3 x 50 mL) and with brine (50 mL). Finally it was dried over magnesium sulfate and the solvent was removed under reduced pressure. The crude product was purified by flash

column chromatography (pentane: EtOAc 10-100%). The product was collected as a brown-orange solid (268 mg, 509 µmol, 85%).

^{1}H NMR (600 MHz, C_6D_6) δ = 8.34 (d, *J* = 1.5 Hz, 1H), 8.15 (d, *J* = 8.4 Hz, 1H), 7.82 (dd, *J* = 8.0, 1.5 Hz, 2H), 7.73 (d, *J* = 7.8 Hz, 1H), 7.67 (dd, *J* = 7.8, 1.5 Hz, 1H), 7.65 – 7.61 (m, 3H), 7.53 (dd, *J* = 7.8, 1.6 Hz, 1H), 7.38 (d, *J* = 1.6 Hz, 1H), 7.29 (ddd, *J* = 8.2, 6.7, 1.2 Hz, 1H), 7.26 (d, *J* = 8.1 Hz, 1H), 7.10 (ddd, *J* = 8.3, 6.7, 1.3 Hz, 1H), 6.82 – 6.76 (m, 2H), 6.54 – 6.49 (m, 2H), 6.26 – 6.20 (m, 2H), 4.18 (p, *J* = 6.6 Hz, 1H), 3.18 (dd, *J* = 15.0, 5.6 Hz, 1H), 2.88 (s, 2H), 2.75 (s, 2H), 2.32 (d, *J* = 15.0 Hz, 1H), 1.20 (d, *J* = 6.8 Hz, 3H) ppm.

^{13}C NMR (151 MHz, C_6D_6) δ = 150.8, 147.7, 146.6, 146.1, 141.5, 140.8, 139.4, 139.2, 138.6, 138.5, 137.0, 133.2, 132.8, 132.0, 131.8, 131.1, 130.6, 129.0, 128.4, 127.5, 126.2, 125.8, 125.4, 124.7, 124.2, 123.3, 120.4, 119.7, 115.6, 115.2, 45.6, 42.1, 19.1 ppm.

^{1}H NMR (600 MHz, $CDCl_3$) δ = 8.17 (d, *J* = 1.5 Hz, 1H), 8.02 (dd, *J* = 8.3, 1.2 Hz, 1H), 7.93 (t, *J* = 8.9 Hz, 2H), 7.84 (d, *J* = 7.9 Hz, 1H), 7.75 (d, *J* = 7.8 Hz, 1H), 7.60 – 7.51 (m, 5H), 7.46 – 7.39 (m, 2H), 6.93 (d, *J* = 1.5 Hz, 1H), 6.87 – 6.80 (m, 2H), 6.60 – 6.54 (m, 2H), 6.49 – 6.44 (m, 2H), 4.44 (p, *J* = 6.6 Hz, 1H), 3.73 (s, 4H), 3.58 (dd, *J* = 15.1, 5.7 Hz, 1H), 2.78 (d, *J* = 15.1 Hz, 1H), 1.47 (d, *J* = 6.8 Hz, 3H) ppm.

^{13}C NMR (151 MHz, $CDCl_3$) δ = 151.0, 147.5, 145.9, 145.4, 140.8, 140.0, 138.6, 138.5, 138.0, 137.8, 136.6, 132.9, 132.6, 131.9, 131.1, 130.9, 130.2, 128.9, 128.2, 127.9, 127.7, 127.1, 125.7, 125.2, 125.2, 124.2, 124.2, 124.2, 122.5, 119.9, 119.2, 115.7, 115.2, 45.4, 42.1, 19.5 ppm.

IR (ATR): $\tilde{\nu}$ = 3456 (w), 3367 (w), 3209 (w), 3024 (w), 2963 (w), 2923 (w) 1616 (m), 1519 (m), 1461 (m), 1410 (w), 1273 (w), 1184 (w), 1057 (w), 811 (vs), 757 (w), 657 (w), 565 (w), 536 (m), 476 (w) cm^{-1}.

HRMS (ESI pos): calculated for $C_{39}H_{31}N_2^+$ ($[M+H]^+$): 527.2482 found: 527.2487.

***N,N'*-((9-(2-Methyl-2,3-dihydro-1*H*-cyclopenta[*a*]naphthalen-1-ylidene)-9*H*-fluorene-2,7-diyl)bis(4,1-phenylene))bis(1-phenylmethanimine) (2)**

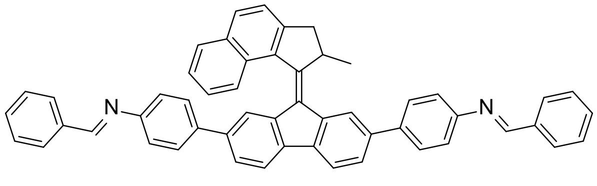

Motor **1** (2.5 mg, 4.7 µmol, 1.0 equiv.) was dissolved in 0.3 mL toluene and 1 µL benzaldehyde (**8**, 9.5 µmol, 2.0 equiv.) was added together with 3Å molecular sieves. The mixture was heated to 80 °C for 16 h. The solvent was removed under reduced pressure and the product was obtained as a yellow solid (3.3 mg, 4.7 µmol, 99%).

^{1}H NMR (400 MHz, C_6D_6): δ = 8.39 (s, 1H), 8.33 (s, 1H), 8.14 (d, J = 9.5 Hz, 2H), 7.94 – 7.76 (m, 9H), 7.68 (dd, J = 11.2, 8.3 Hz, 2H), 7.56 (d, J = 8.0 Hz, 1H), 7.45 (s, 1H), 7.39 (d, J = 8.0 Hz, 2H), 7.29 (dd, J = 11.1, 7.9 Hz, 2H), 7.14 – 7.10 (m, 9H), 6.97 (d, J = 8.1 Hz, 2H), 4.20 (t, J = 6.5 Hz, 1H), 3.22 (dd, J = 15.2, 5.5 Hz, 1H), 2.37 (d, J = 15.2 Hz, 1H), 1.22 (d, J = 6.7 Hz, 3H) ppm.

^{13}C NMR (101 MHz, C_6D_6): δ = 159.9, 151.9, 151.7, 151.4, 148.0, 141.6, 140.5, 140.3, 139.6, 139.1, 138.9, 138.7, 137.1, 133.3, 131.5, 131.2, 129.3, 129.3, 129.2, 128.9, 128.9, 128.6, 128.4, 128.2, 128.1, 127.9, 126.7, 126.3, 125.6, 125.3, 124.3, 123.7, 122.2, 121.7, 120.7, 120.0, 45.6, 42.1, 19.1 ppm.

HRMS (ESI pos): calculated for $C_{53}H_{39}N_2^+$ ([M+H]$^+$): 703.3108 found: 703.3115.

4,4'-(9,9-Dimethyl-9*H*-fluorene-2,7-diyl)dianiline (4)

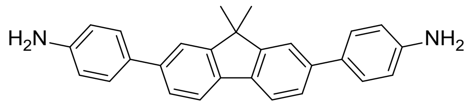

Spacer **5** (2.10 g, 5.97 mmol, 1.0 equiv.),[8] 4-aminophenylboronic pinacol ester (**6**, 2.88 g, 13.1 mmol, 2.2 equiv.) and Pd(dppf)Cl_2 (218 mg, 298 µmol, 5 mol%) were mixed with 10 mL 1,4-dioxane and 1M aqueous potassium carbonate (24 mL, 24 mmol, 4.0 equiv.). The mixture was degassed by applying vacuum until the solvent started bubbling and was saturated with N_2. This process was repeated three times. Subsequently the mixture was stirred at 80 °C for 16 h under N_2 atmosphere. The reaction mixture was cooled down to room temperature, ethyl acetate and water were added, the phases were separated and the aqueous phase was extracted with ethyl acetate (3 x 100 mL). The combined organic phase was washed with half sat. brine (3 x 100 mL). Finally, it was dried over magnesium sulfate and the solvent was removed under reduced pressure. The crude product was purified by flash column chromatography (pentane:EtOAc 10-100%). The product was collected as a brown-orange solid (1.50 g, 3.98 mmol, 67%).

^{1}H NMR (400 MHz, $CDCl_3$): δ =7.74 (d, J = 7.9 Hz, 2H), 7.59 (d, J = 1.6 Hz, 2H), 7.60 – 7.44 (m, 8H), 6.83 – 6.74 (m, 4H), 3.74 (s, 2H), 1.56 (s, 6H) ppm.

^{13}C NMR (101 MHz, $CDCl_3$): δ = 154.5, 145.9, 140.3, 137.5, 132.2, 128.2, 125.6, 120.7, 120.2, 115.6, 77.5, 77.2, 76.8, 47.1, 27.5 ppm.

IR (ATR): $\tilde{\nu}$ = 3206 (w), 3442 (w), 3351 (w), 3021 (w), 2956 (w), 2922 (w), 2863 (w), 1606 (w), 1519 (m), 1463 (m), 1409 (w), 1269 (w), 1183 (w), 1127 (w), 1081 (w), 815 (vs), 745 (w), 633 (w), 560 (w), 529 (m), 470 (m) cm^{-1}.

HRMS (ESI pos) calculated for $C_{27}H_{25}N_2$ ([M+H]$^+$): 377.2012, found 377.2020.

Polymer Synthesis

Motor:Spacer 1:1 Polymer (m$_{50}$-P)

Motor amine **1** (48.7 mg, 92.5 µmol, 1.5 equiv.) and spacer amine **4** (34.8 mg, 92.5 µmol, 1.5 equiv.) were dissolved in a degassed (freeze-pump-thaw) solvent mixture of dioxane and mesitylene (4:1, 2 mL). Triformylbenzene (20.0 mg, 123 µmol, 2.0 equiv.) and $Sc(OTf)_3$ (3.00 mg, 6.2 µmol) were dissolved separately in the same solvent mixture (2 mL). The first solution was added to the latter in a sealed vial, stirred briefly and it was kept for three days without light exposure.

^{1}H ssNMR (MAS, 600 MHz): δ = 6.6, 2.5, 0.9 ppm.

^{13}C ssNMR (CP-MAS, 151 MHz): δ = 154.1, 149.4, 137.8, 126.8, 120.2, 46.8, 42.6, 26.5, 17.9 ppm.

IR (ATR): $\tilde{\nu}$ = 1700 (w), 1622 (w), 1593 (w), 1507 (w), 1463 (m), 1250 (w), 1137 (w), 1011 (w), 970 (w), 811 (vs), 743 (w), 683 (w), 655 (w), 538 (w) cm^{-1}.

Motor Polymer (m$_{100}$-P)

The motor polymer was synthesized similar to the above-mentioned procedure, but without the addition of the spacer amine and an increased amount of motor amine **1** (97.4 mg, 185 µmol, 3.0 equiv.).

^{1}H ssNMR (MAS, 600 MHz): δ = 6.5, 2.4, 0.8 ppm.

^{13}C ssNMR (CP-MAS, 151 MHz): δ = 148.5, 137.7, 126.6, 41.6, 18.2 ppm.

IR (ATR): $\tilde{\nu}$ = 1702 (w), 1619 (w), 1589 (w), 1508 (w), 1460 (m), 1406 (w), 1249 (w), 1139 (w), 1012 (w), 969 (w), 810 (vs), 756 (w), 683 (w), 656 (w), 538 (w) cm^{-1}.

Spacer Polymer (m$_0$-P)

The spacer polymer was synthesized similar to the above-mentioned procedure, but without the addition of the motor amine **1** and an increased amount of spacer amine **4** (69.6 mg, 185 µmol, 3.0 equiv.).

^{1}H ssNMR (MAS, 600 MHz): δ = 6.7, 0.9 ppm.

^{13}C ssNMR (CP-MAS, 151 MHz): δ = 156.4, 139.9, 128.8, 122.2, 48.3, 28.3 ppm.

IR (ATR): $\tilde{\nu}$ = 1700 (w), 1623 (w), 1507 (w), 1464 (s), 1250 (w), 1137 (w), 1011 (w), 971 (w), 812 (vs), 743 (w), 683 (w), 654 (w), 566 (w), 538 (w), 467 (w) cm^{-1}.

COF Synthesis

m_{20}-COF

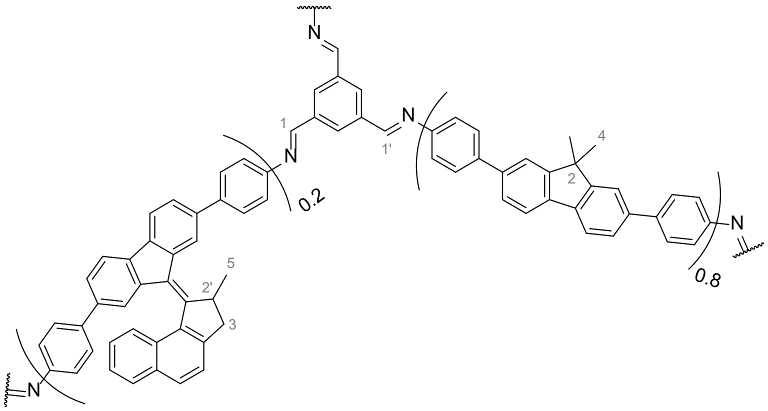

A mixture of 1,3,5-triformyl benzene (6.5 mg, 40 µmol, 2.0 equiv.), spacer **4** (18.1 mg, 48 µmol, 2.4 equiv.) and motor **1** (6.3 mg, 12 mmol, 0.6 equiv.) in mesitylene (2 mL) and aqueous 6 M AcOH (0.2 mL) was heated at 110 °C for 72 h. The precipitate was collected via suction filtration, washed with DMF (10 mL), THF (10 mL) and MeOH (10 mL) and extracted with MeOH in a Soxhlet extractor for 24 h. Supercritical point drying with CO_2 afforded **m_{20}-COF** (27.1 mg, 90%) as an orange-brown solid.

^{1}H ssNMR (MAS, 600 MHz): δ = 6.9 (H-1, H-1', H-Ar), 1.1 (H-3, H-4, H-4', H-5) ppm.

^{13}C ssNMR (CP-MAS, 151 MHz): δ = 154.6 (C-1, C-1'), 148.9, 137.7, 127.2, 121.2, 46.5 (C-2, C-2'), 41.9 (C-3), 26.6 (C-4), 17.8 (C-5) ppm.

IR (ATR): $\tilde{\nu}$ = 3031 (w), 2956 (w), 2863 (w), 1703 (w), 1623 (w), 1592 (w), 1507 (w), 1463 (s), 1250 (w), 1138 (w), 1011 (w), 970 (w), 811 (vs), 743 (w), 683 (w), 655 (w), 565 (w), 539 (w) cm^{-1}.

m_{10} and **m_5-COF** were synthesized under identical conditions to **m_{20}-COF** with adjusted relative amounts of the motor **1** and spacer **4** building blocks (5% motor **1** for m_5 and 10% motor **1** for **m_{10}-COF**).

m_0-COF (pure spacer COF)

The spacer COF was synthesized similar to the above-mentioned procedure, but without the addition of the motor **1** and an increased amount of spacer **4** (22.6 mg, 60 µmol, 3.0 equiv.).

^{1}H ssNMR (MAS, 600 MHz): δ = 6.8, 0.8 ppm.

^{13}C ssNMR (CP-MAS, 151 MHz): δ = 154.2, 150.3, 137.8, 126.8, 120.4, 46.1, 26.1 ppm.

IR (ATR): $\tilde{\nu}$ = 2981 (w), 1701 (w), 1625 (w), 1596 (w), 1508 (w), 1464 (s), 1250 (w), 1139 (w), 1011 (w), 971 (w), 812 (vs), 743 (w), 715 (w), 684 (w), 655 (w), 565 (w), 538 (w), 468 (w) cm^{-1}.

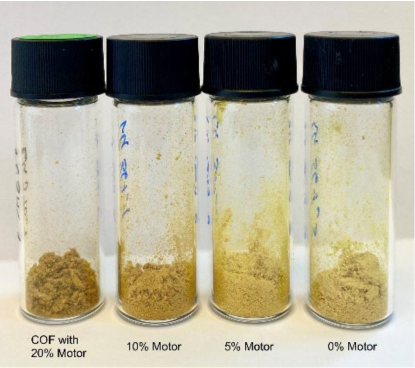

Figure S 1: Photograph of the synthesized COF samples with different fractions of motor building block. Increased motor content causes a red shift in absorption of the resulting COF.

Analytical Data

UV-Vis spectra

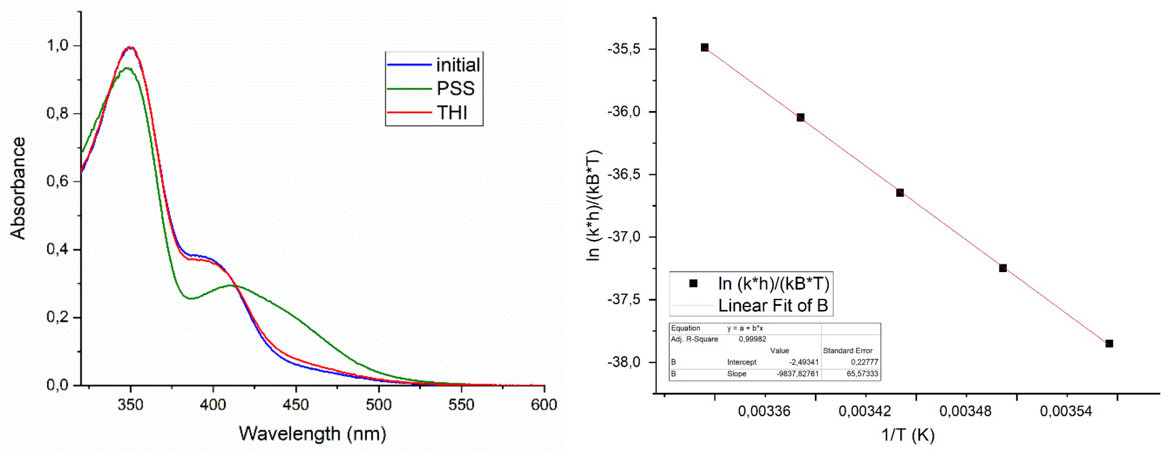

*Figure S 2: **Left:** UV-Vis absorption spectrum of amine motor **1** in acetonitrile (1x10^{-5} M) at 5 °C, initally (blue), after irradiation with 365 nm to the photostationary state (PSS, green) and after thermal relaxation of the metastable state (THI, red). **Right:** Eyring plot analysis of the thermal isomerisation of amine motor **1** from metastable isomer to stable isomer in acetonitrile.*

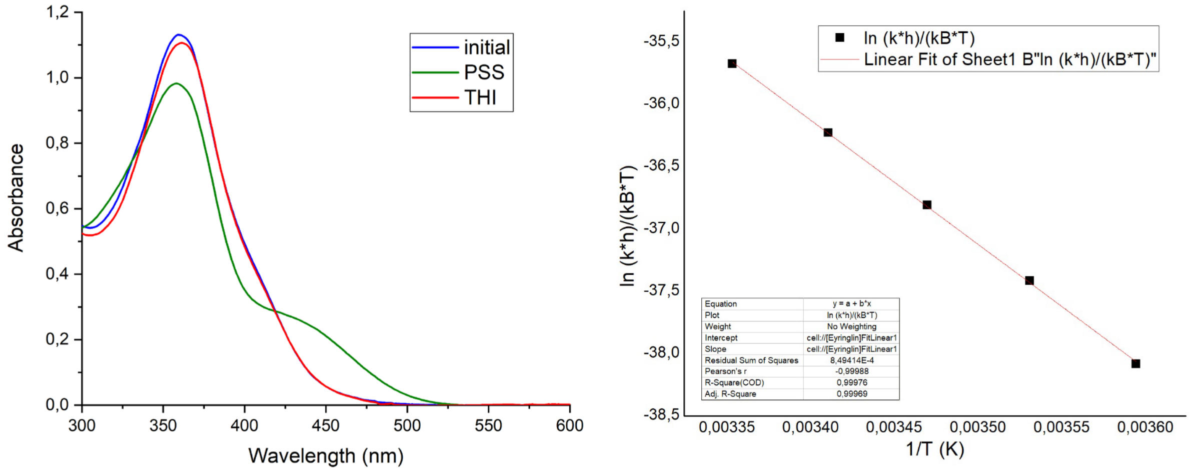

*Figure S 3: **Left:** UV-Vis absorption spectrum of imine motor **2** in acetonitrile (0.7 mM) at 5 °C, initally, after irradiation with 365 nm to the photostationary state and after thermal relaxation of the metastable state. **Right:** Eyring plot analysis of the thermal isomerisation of imine motor **2** from metastable isomer to stable isomer in acetonitrile.*

FT-IR Spectra

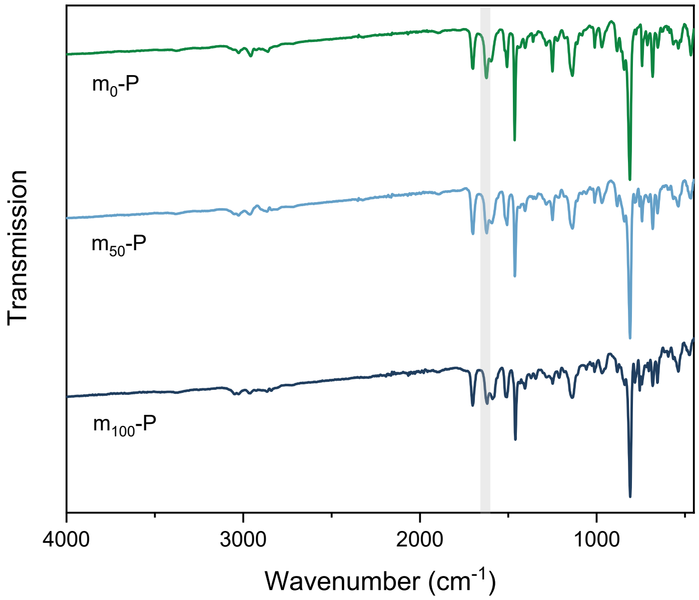

*Figure S 4: FT-IR spectra of **m_0-polymer** (green), **m_{50}- polymer** (light blue) and **m_{100}-polymer** (dark blue). Grey area highlights imine C=N vibration bands.*

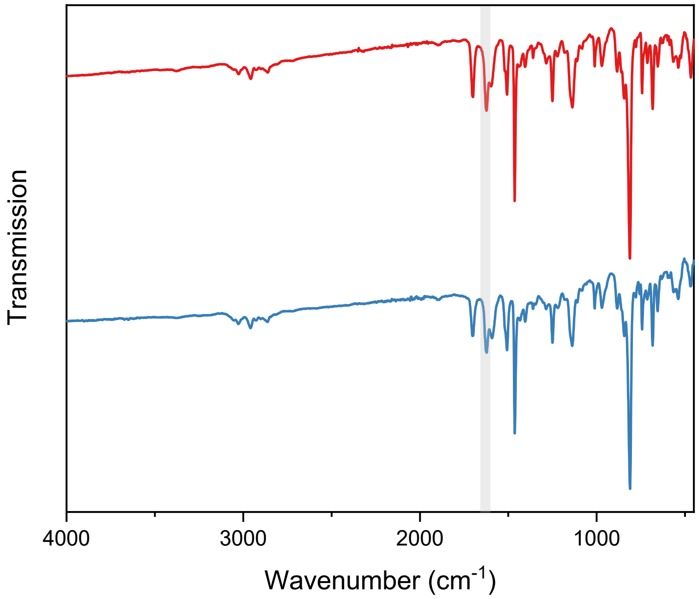

*Figure S 5: FT-IR spectra of **m_0-Polymer** (red) and **m_{20}-COF** (blue). The spectra do not show significant differences, underlining their compositional relation. Grey area highlights imine C=N vibration bands.*

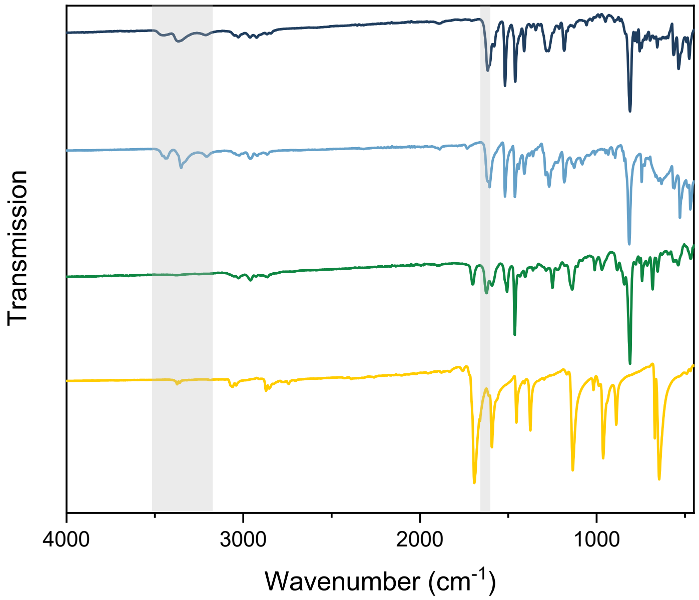

*Figure S 6: FT-IR spectra of motor building block (dark blue), spacer building block (light blue), **m_{20}-COF** (green) and aldehyde building block (yellow). The absence of amine (N-H) and simultaneous appearance of imine (C=N) vibration bands in the COF underlines the successful condensation of the starting materials. Grey areas highlight aldehyde C-H, amine N-H and imine C=N vibration bands.*

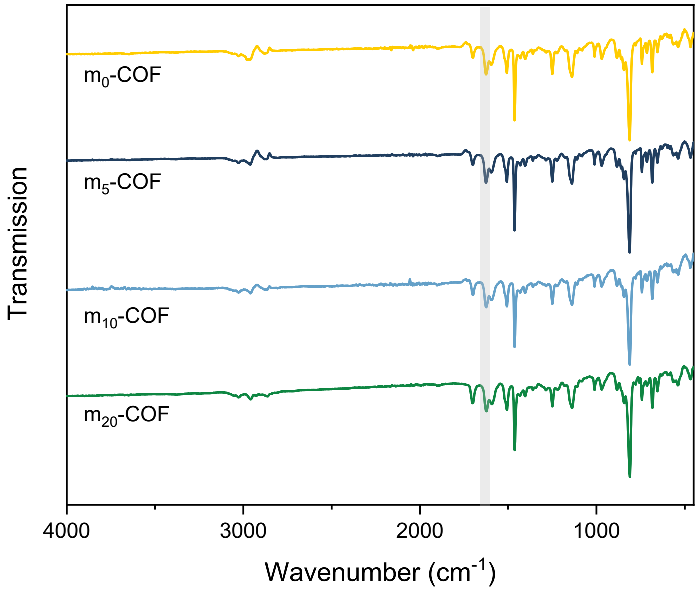

Figure S 7: FT-IR spectra of COFs with various motor content. Grey area highlights imine C=N vibration bands.

Raman Spectra

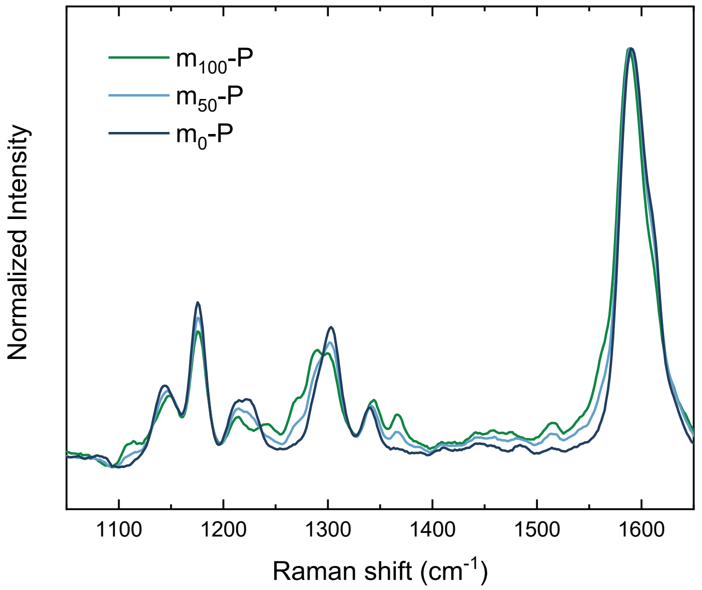

Figure S 8: Raman spectra (785 nm) of various motor containing polymers.

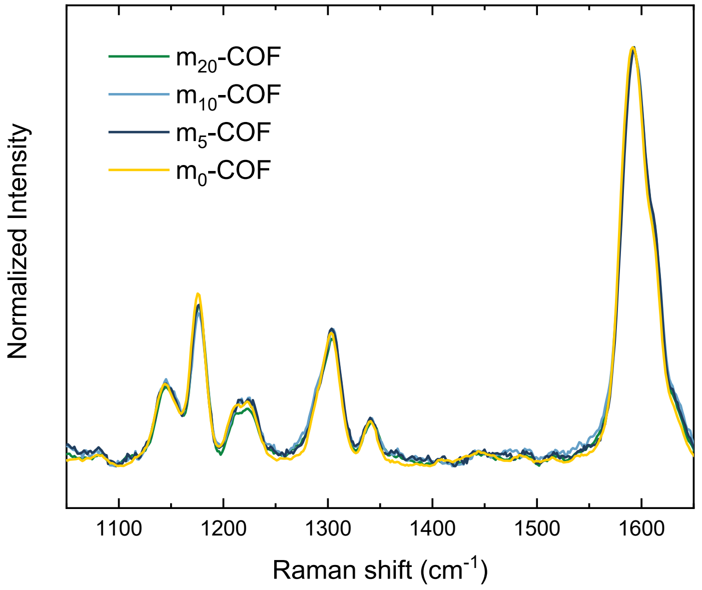

Figure S 9: Raman spectra (785 nm) of various motor containing COFs. Additional bands could not be attributed to the motor unit, due to the low motor content in the materials.

PXRD Data and Structure Modeling

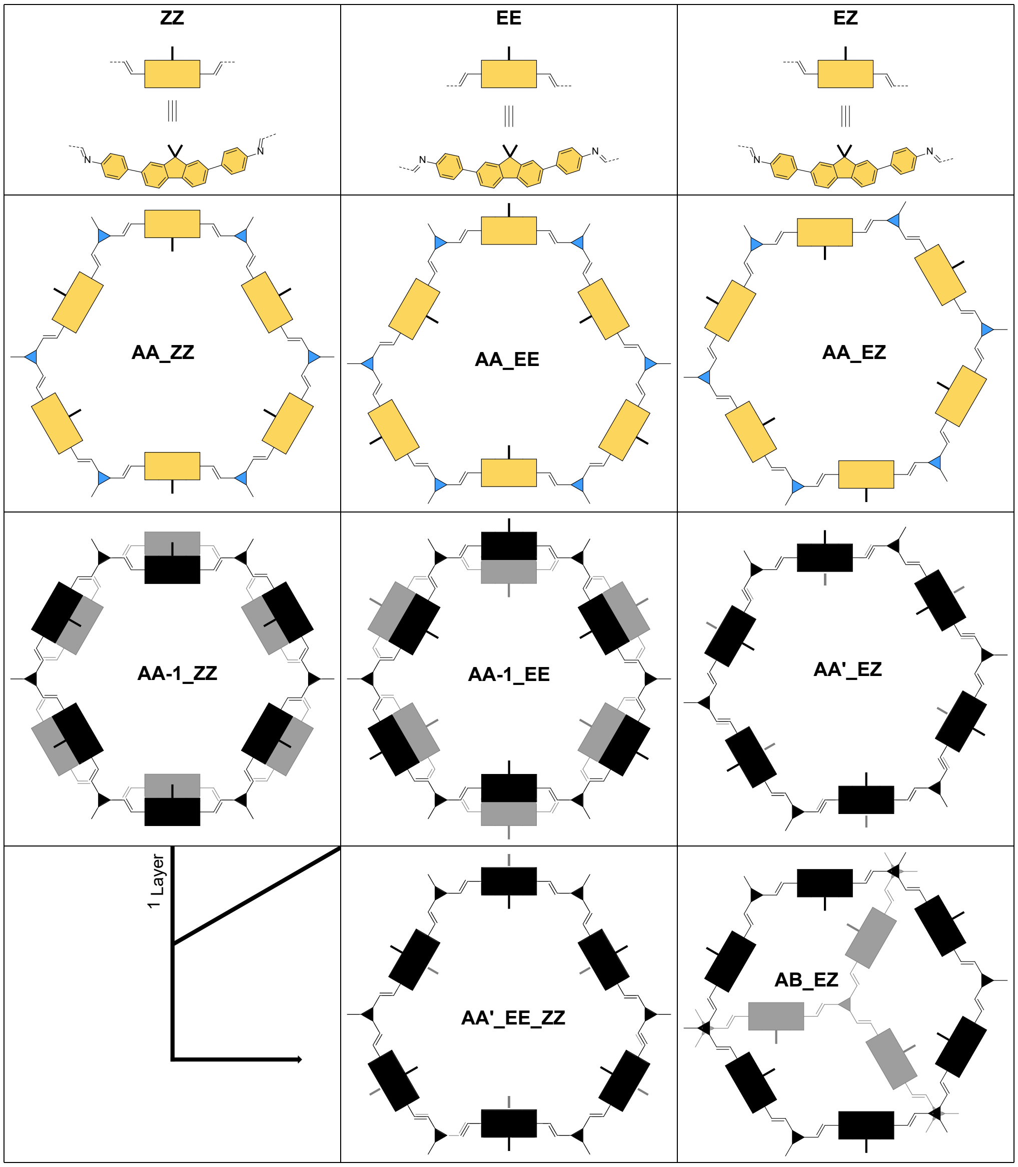

Figure S 10: A collection of possible stacking modes with a simplified pure-spacer COF pore are shown. The structures differ in imine-bond conformation (EE/ZZ/EZ/EE+ZZ) and eclipsed (AA/AA'/AA-1) or staggered (AB) stacking of the layers. To visualize the effect of imine-conformation on the position of the methyl groups, first (black) and second layer (grey) are colored. Depending on the conformation, sterically demanding methyl groups of the building block can either point in the same direction (AA structures) or in opposite directions, impacting interlayer π-π interactions which are mostly inhibited in AA structures, AA-1_ZZ and weak for AB_EZ. AA-1_EE as well as both AA' structures benefit from an alternating configuration of methyl groups pointing towards the pore channel, which enables interaction of the aromatic systems across the layers. A comparison of these structures also highlights that the apparent pore-diameter is not only defined by the building blocks but also based on imine-bond orientation and stacking of the layers. In AA and AA' the imine bonds between layers are fully eclipsed (parallel), whereas in AA-1 structures these bonds point in opposite directions.

Table S 1: Comparison of selected stacking models for a simplified pure-spacer COF. The cell parameters, pore-diameters (smallest carbon-carbon distance across the pore) and total energies, as obtained from Forcite geometry and cell optimization in Material Studio are shown. The AA-1_EE was selected as the model for the structure refinement.

Model	Cell parameter a/b [Å]	Cell parameter c [Å]	Space group	Pore diameter [Å]	Total energy [kcal/mol]
AA-1_EE	44.35	7.09	$P6_3mcm$	38.63	366.96
AA'_EE_ZZ	44.27	7.17	$P\bar{6}2m$	38.74	405.46
AA'_EZ	44.36	7.16	$P6_3m$	38.80	404.65

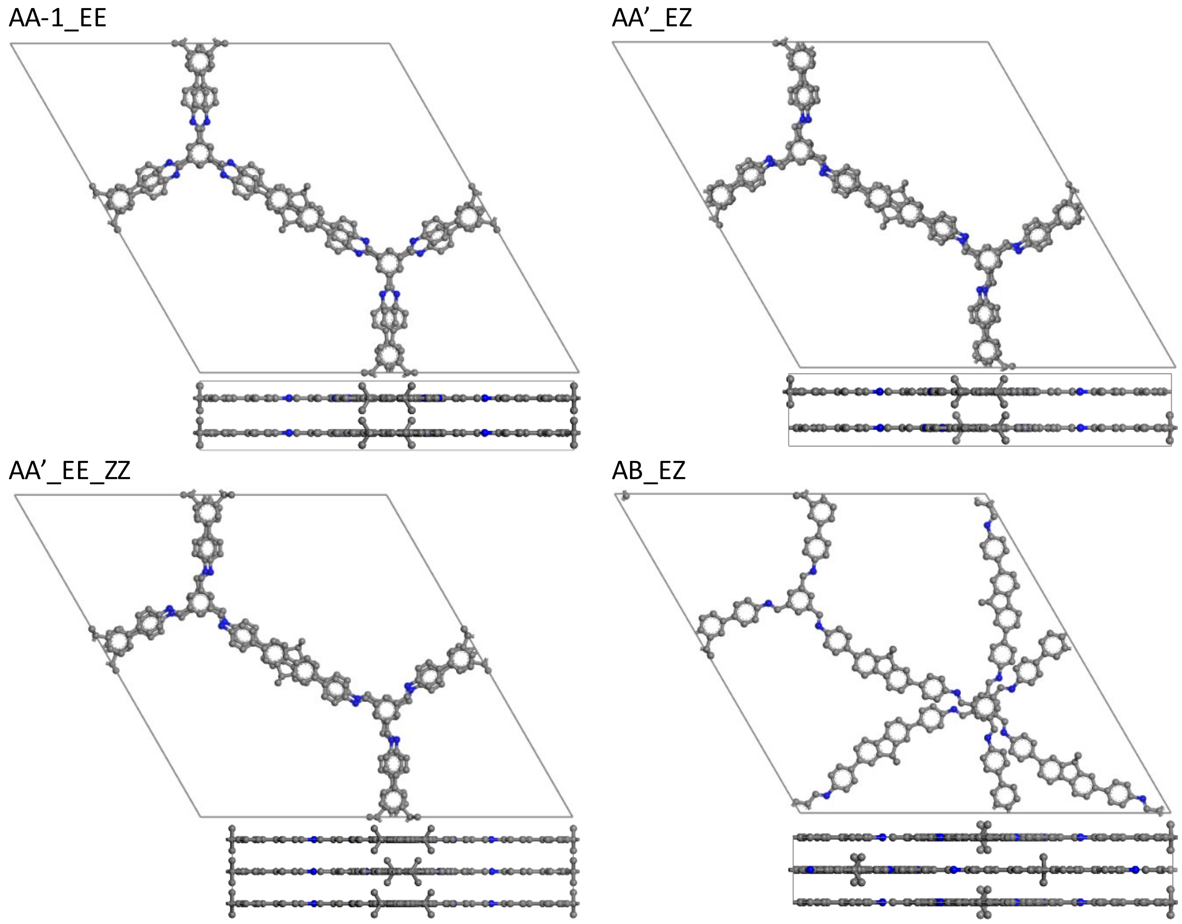

Figure S 11: Comparison of selected stacking models (unit cells, hydrogen omitted for clarity) for a simplified pure-spacer COF. In all cases except AB_EZ, the synchronization of imine-bonds across the layers allows for acceptable stacking interaction despite the curvature of the substituted fluorene building block. The trade-off between sterically demanding methyl groups and stacking interactions is best met in the AA-1_EE model with lowest total energy. In AA and AA' the imine bonds between layers are fully eclipsed (parallel), whereas in AA-1 structures these bonds point in opposite directions. The curvature of the fluorene fragment together with the steric demand of the methyl groups create different stacking environments and thus imine configurations in the material energetically less favorable (see Table S1). In contrast to straight aldehydes in other materials adopting an EZ imne bond configuration, the AA-1_EE configuration is energetically favored for our materials and allows optimal interlayer interactions.

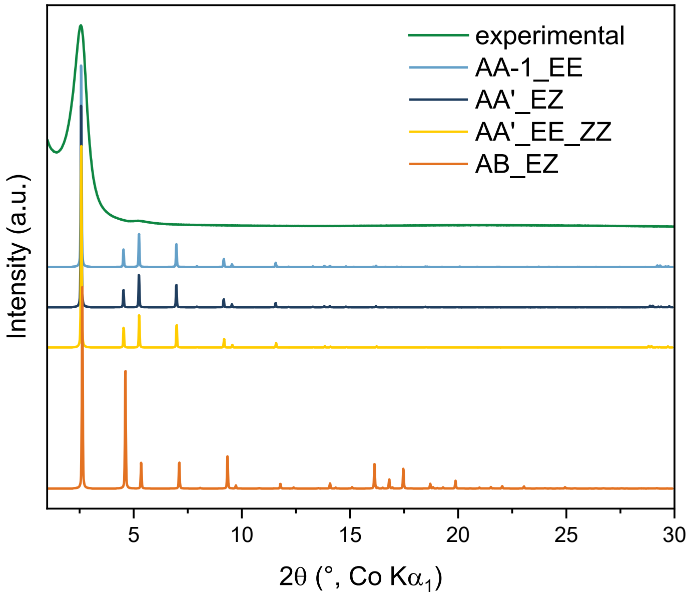

*Figure S 12: Comparison of calculated XRPD patterns of selected stacking models with an experimental pattern of **m20-COF**. Intensities in the AB_EZ model show drastic deviations from the experimental pattern. The patterns were simulated for a simplified pure-spacer COF as shown in Figure S 11.*

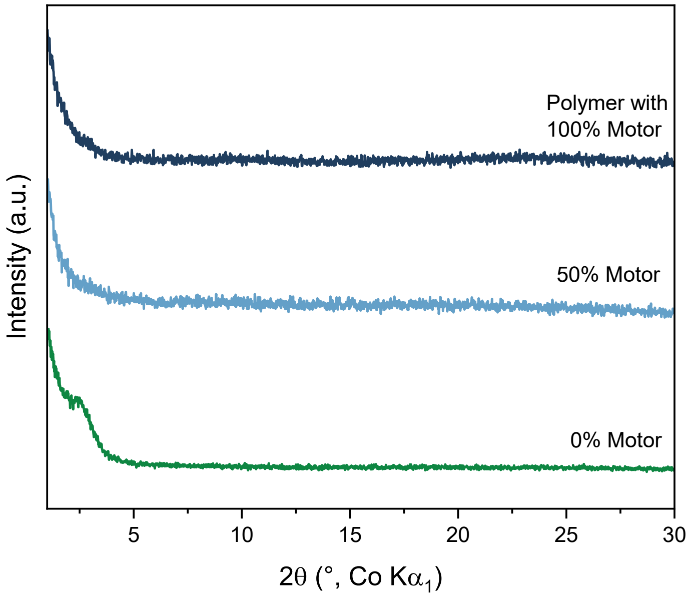

Figure S 13: XRPD patterns of polymers with various motor content.

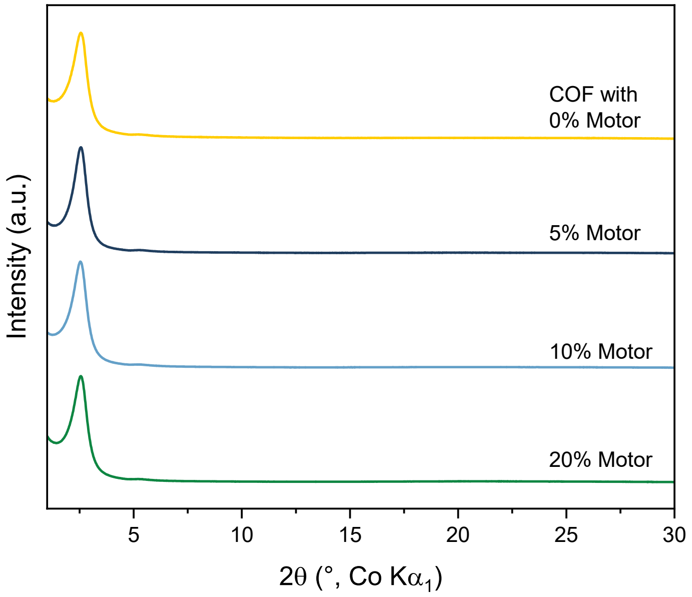

Figure S 14: XRPD patterns of COFs with various motor content.

Pair Distribution Function (PDF) Analysis

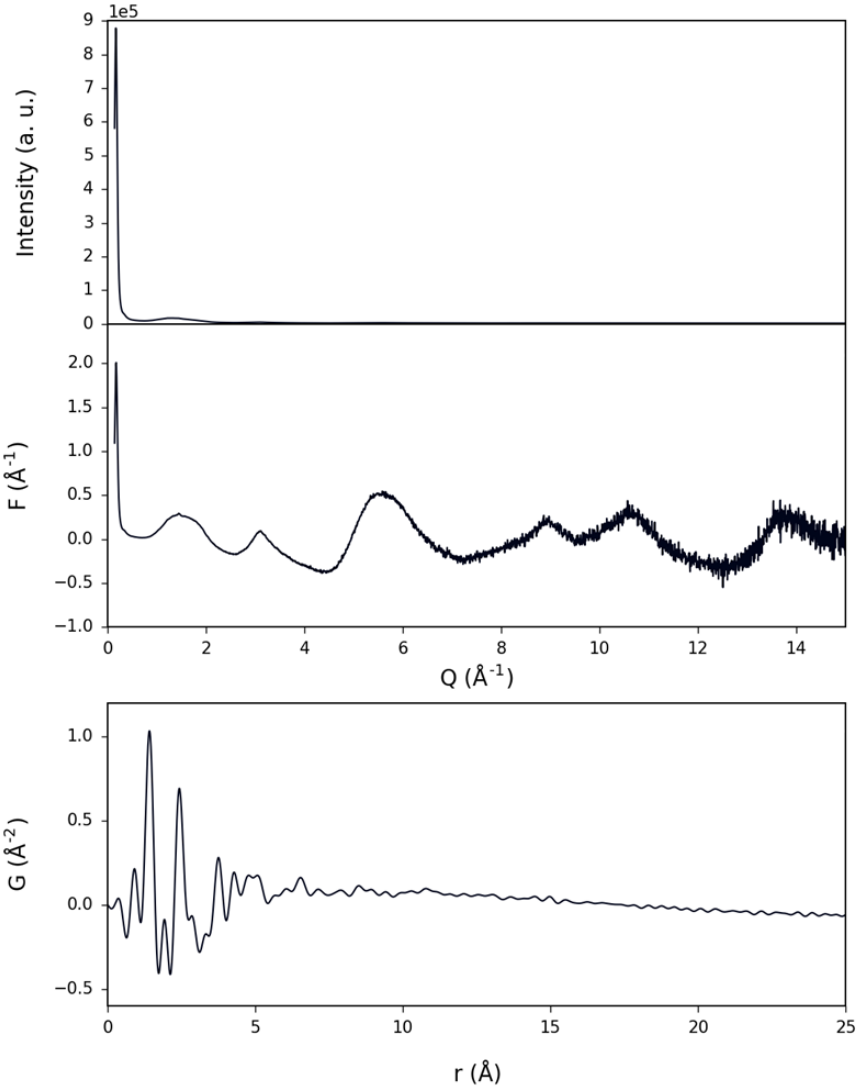

Figure S 15: Visualization of total scattering intensities (top), reduced structure function (middle), and the resulting pair distribution function (bottom). The 100 Bragg peak indicates in-plane crystallinity of the 2D layers. The resulting PDF shows the characteristic intralayer local structure, and a sloping baseline indicating the presence of some pore structure. Unlike other 2D COFs, we do not observe any interlayer correlations, indicating that there is no coherent relationship between different layers.

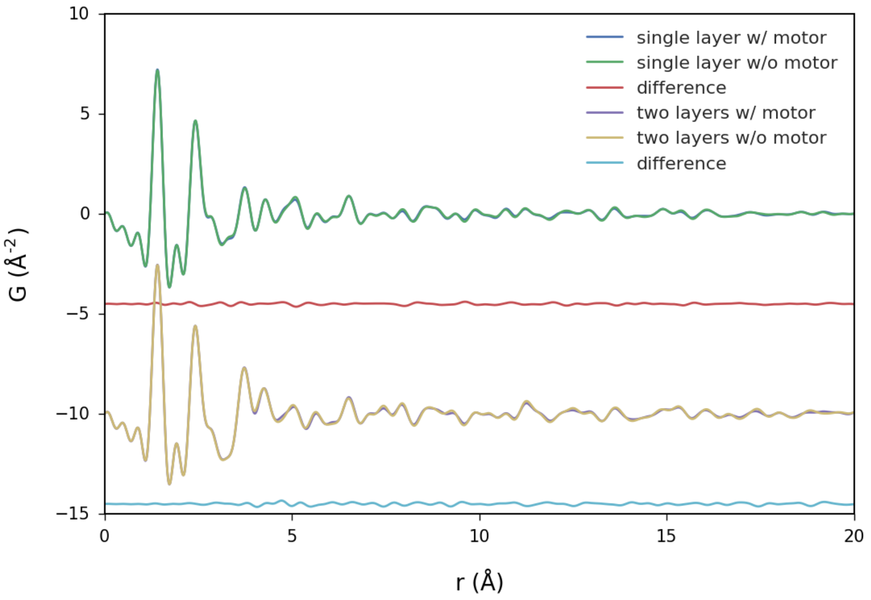

Figure S 16: Simulations of individual layers (AA-1_EE model) with and without a single motor-unit are compared. There is almost no difference, indicating that the motor units cannot be distinguished without some long range ordering, because the intramolecular bond distances are too similar to the backbone of the structure.

NMR Spectra

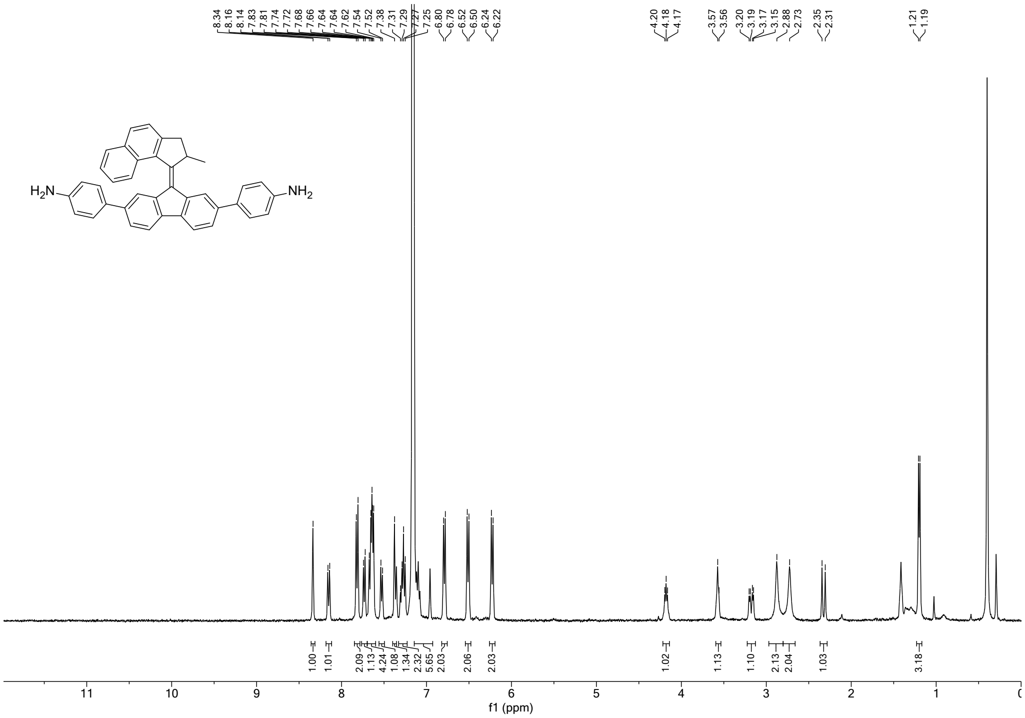

*Figure S 17: ^{1}H NMR of motor **1** in C_6D_6.*

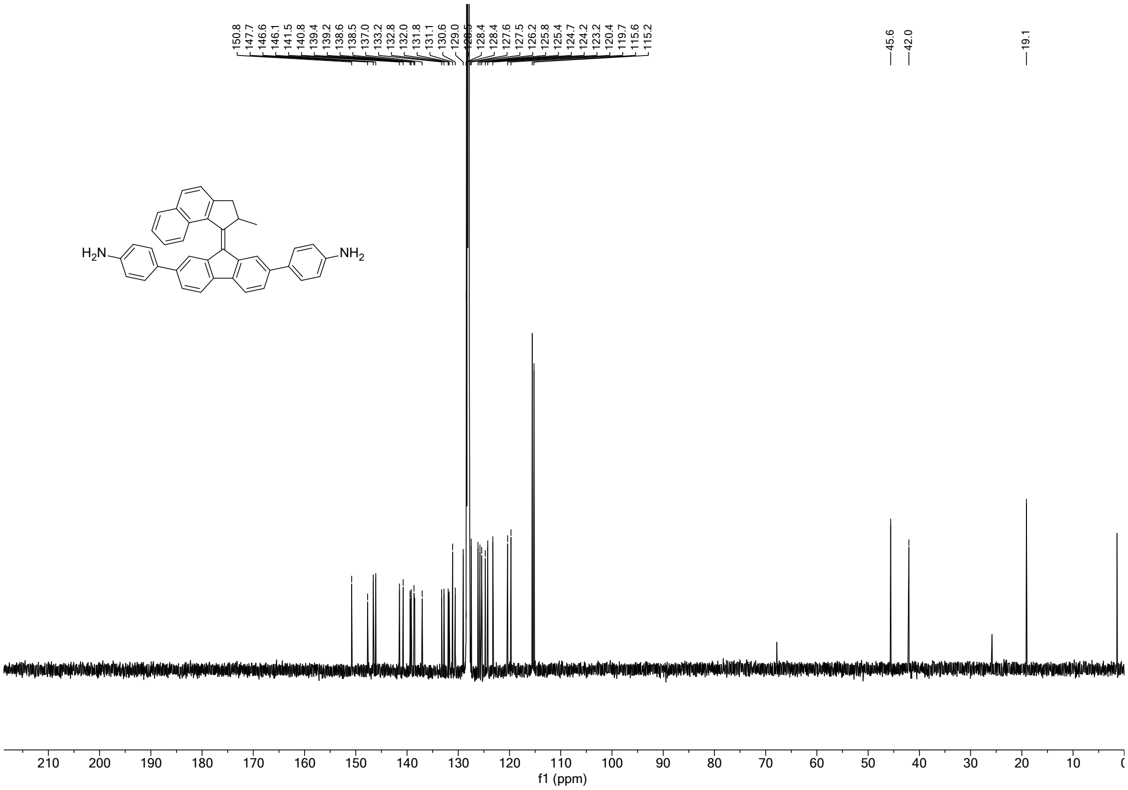

*Figure S 18: ^{13}C NMR of motor **1** in C_6D_6.*

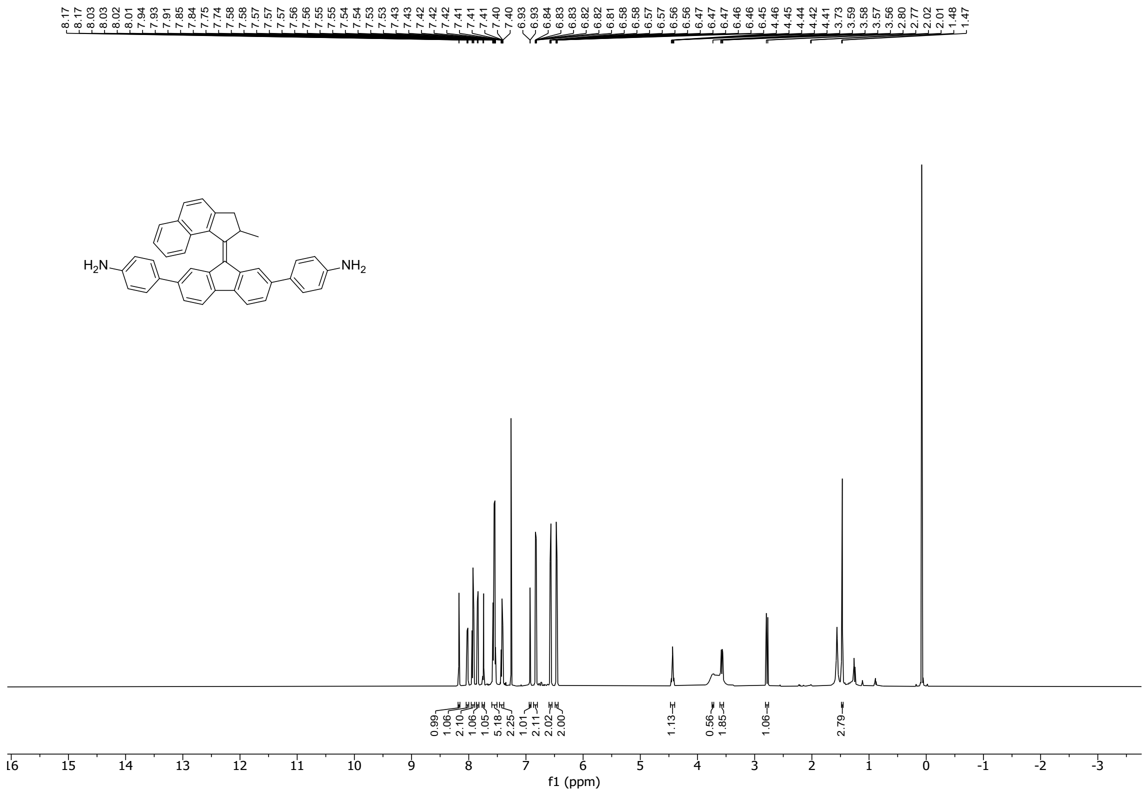

*Figure S 21: ^{1}H NMR of motor **1** in $CDCl_3$.*

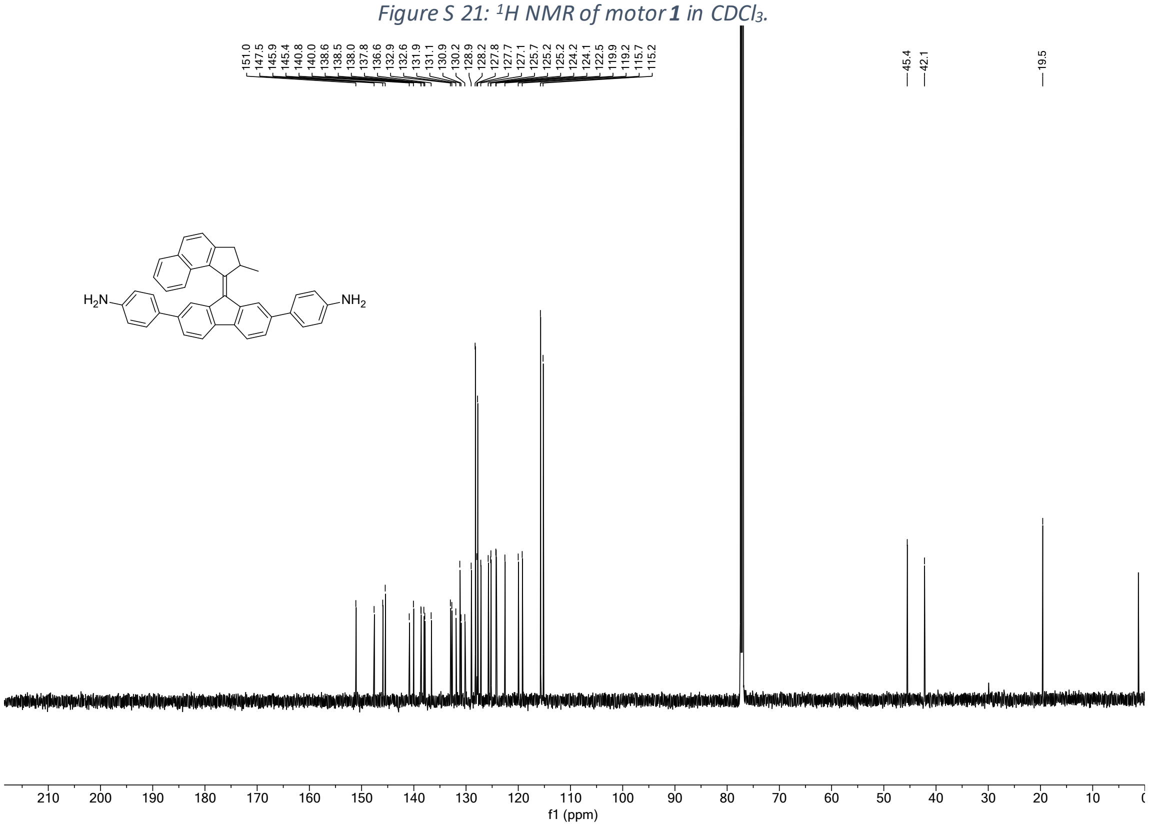

*Figure S 22: ^{13}C NMR of motor **1** in $CDCl_3$.*

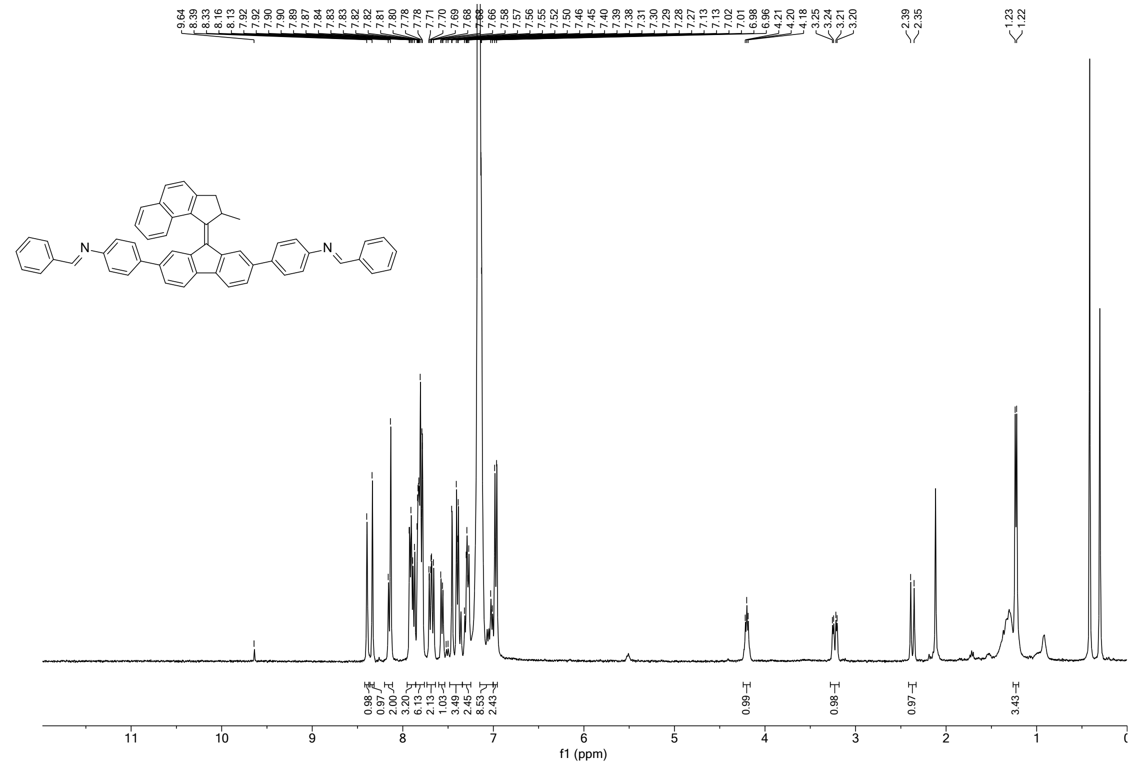

*Figure S 19: ^1H NMR of imine motor **2** in C_6D_6.*

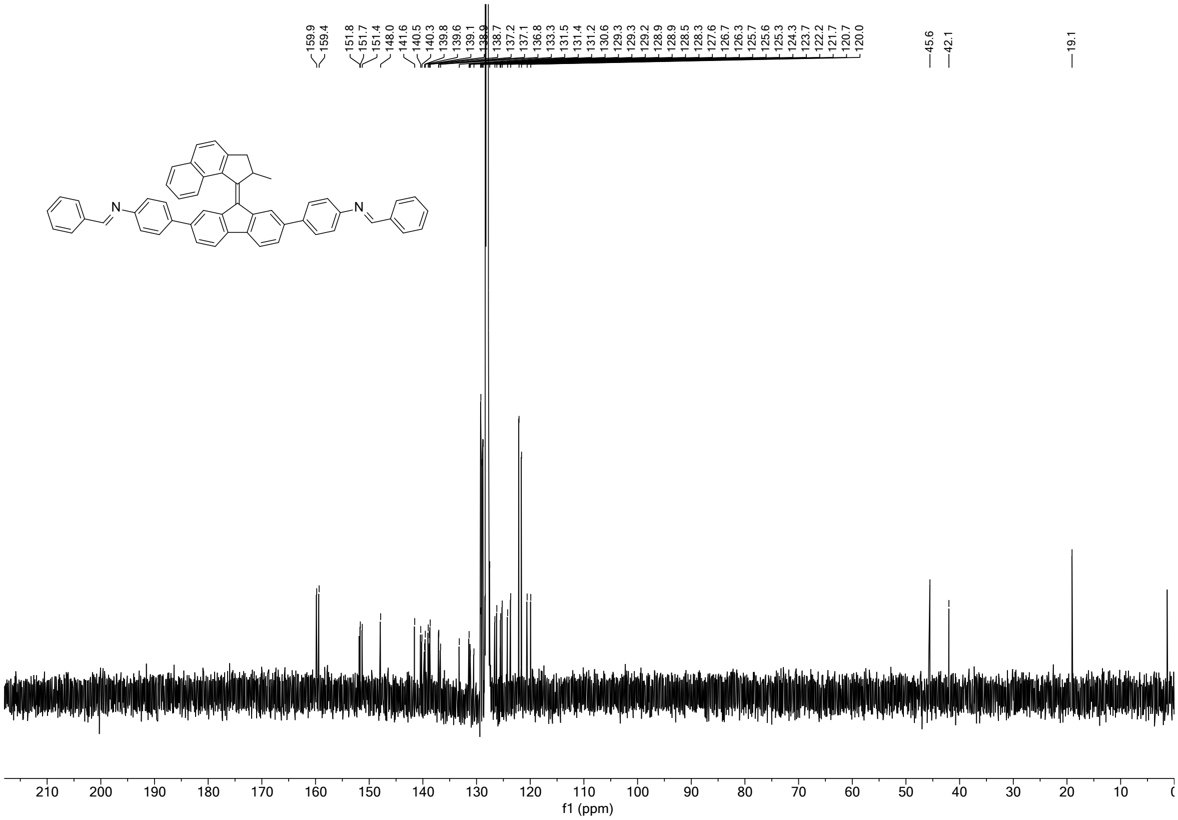

*Figure S 20: ^{13}C NMR of imine motor **2** in C_6D_6.*

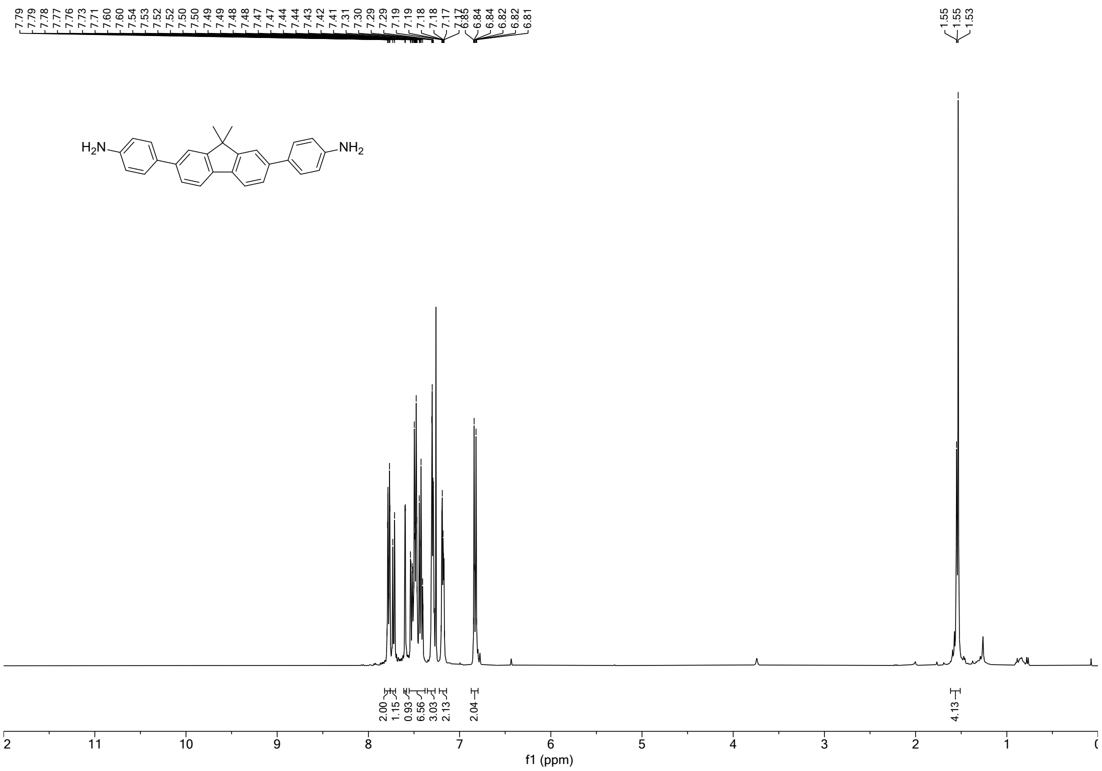

Figure S 21: ^{1}H NMR of spacer 4 in $CDCl_3$.

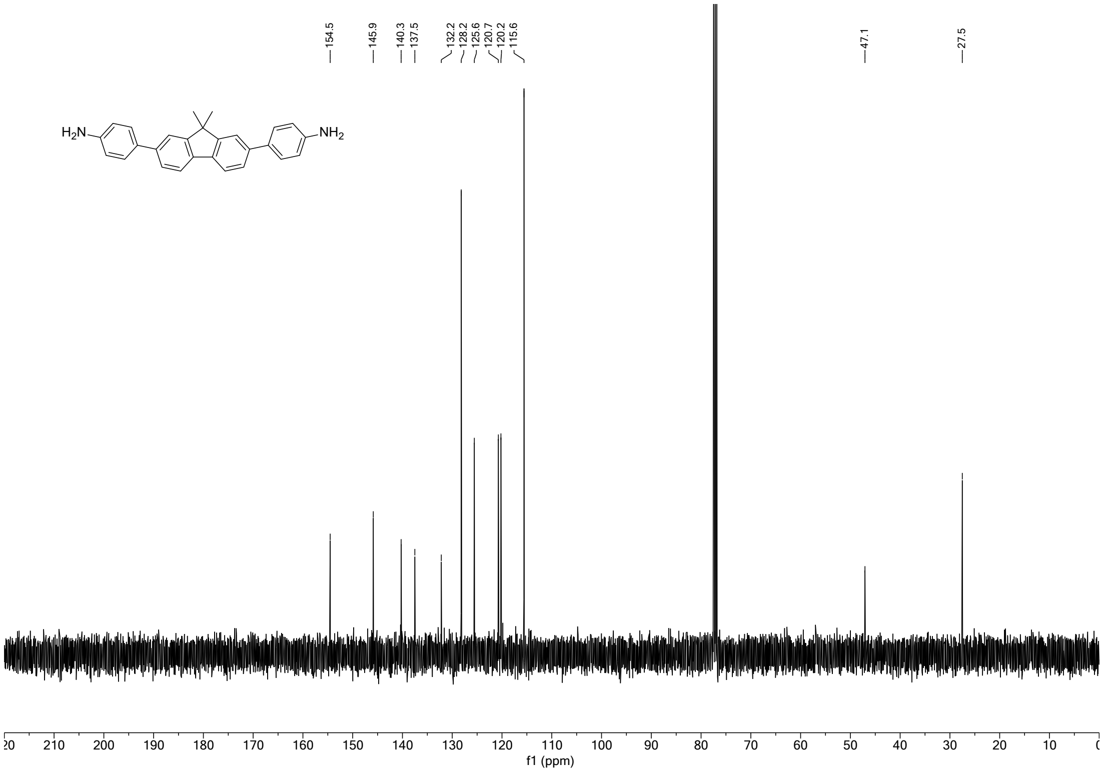

*Figure S 22: ^{13}C NMR of spacer **4** in $CDCl_3$.*

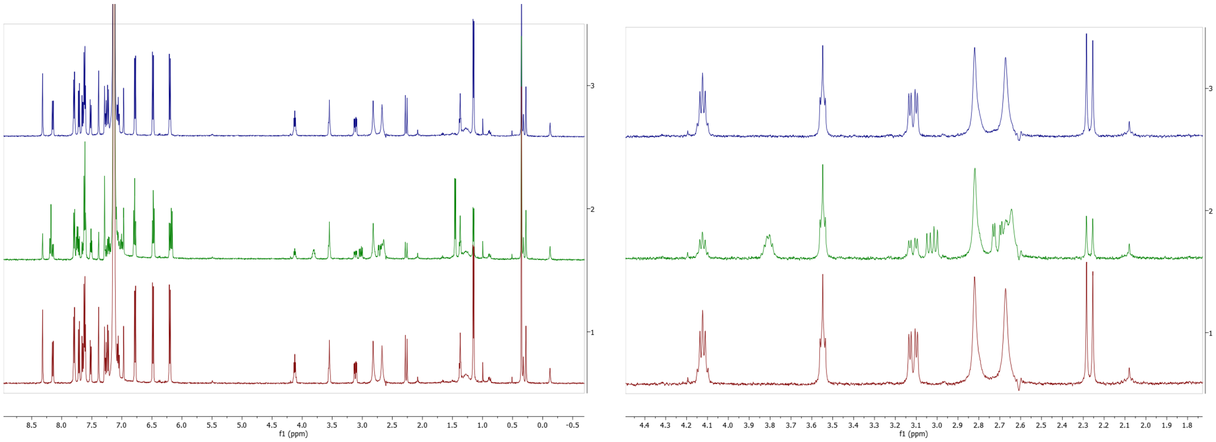

*Figure S 23: ^{1}H NMR spectra of amine motor **1** in C_6D_6 (4.75 mm) at 10 °C initially (top), after irradiation with 385 nm to the photostationary state of 54:46 (metastable:stable) (middle) and after full thermal relaxation at 10 °C (full spectrum and selected region).*

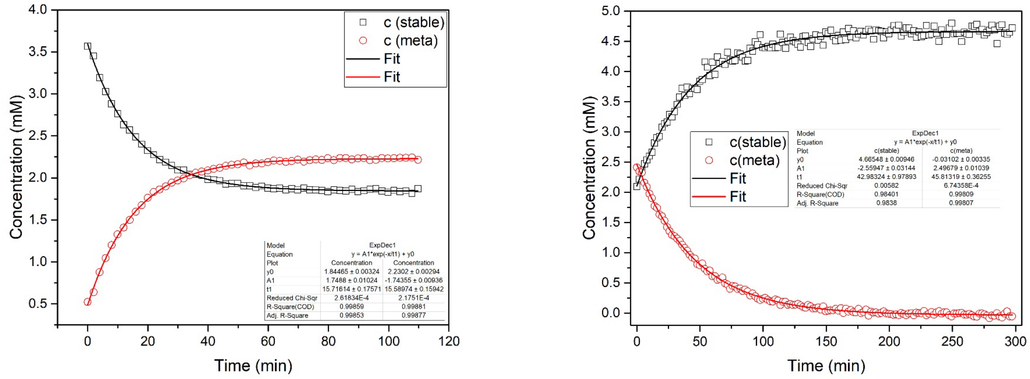

*Figure S 24: Change of concentration of the stable amine motor **1** (black squares) and metastable **1** (red circles) during irradiation with 365 nm at 10 °C (left) and during thermal relaxation at 10 °C (right) in ^{1}H-NMR (C_6D_6, 4.75 mm).*

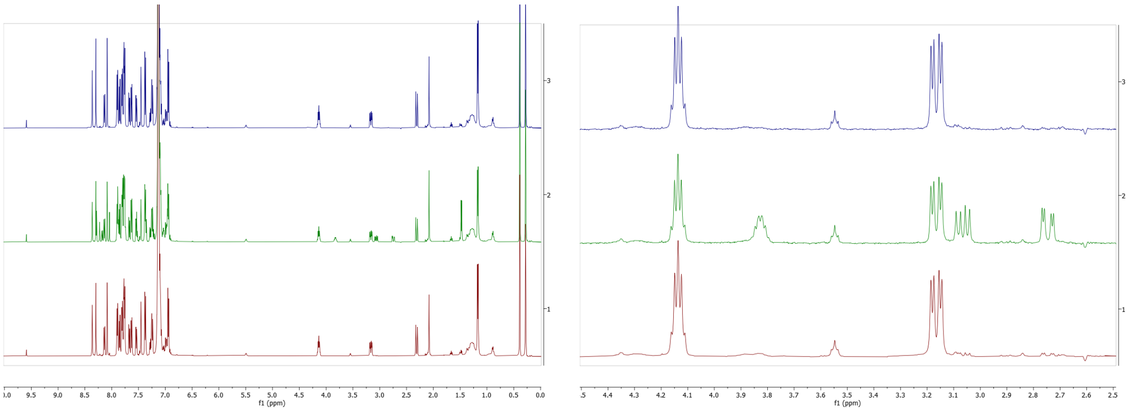

*Figure S 25: ^{1}H NMR spectra of imine motor **2** in C_6D_6 (9.40 mm) at 10 °C initially (top), after irradiation with 385 nm to the photostationary state of 22:78 (metastable:stable) (middle) and after full thermal relaxation at 10 °C (full spectrum and selected region).*

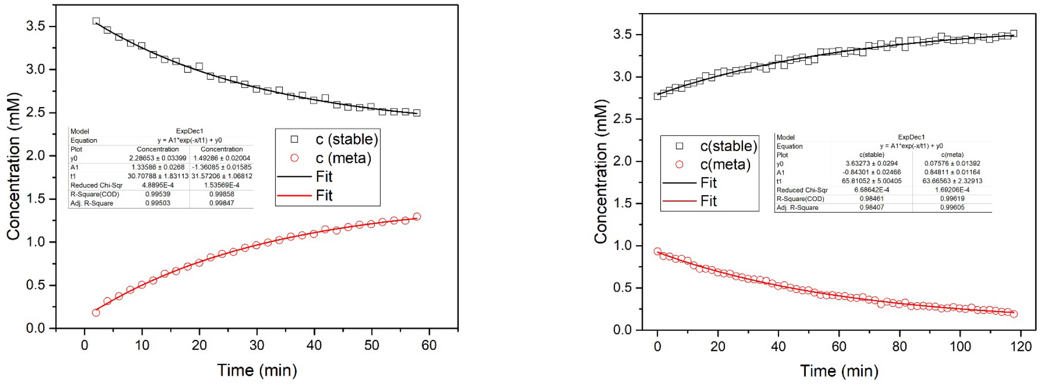

*Figure S 26: Change of concentration of the stable imine motor **2** (black squares) and metastable **2** (red circles) during irradiation with 385 nm at 10 °C (left) and during thermal relaxation at 10 °C (right) in ^1H NMR (C_6D_6, 4.75 mm).*

ssNMR Spectra

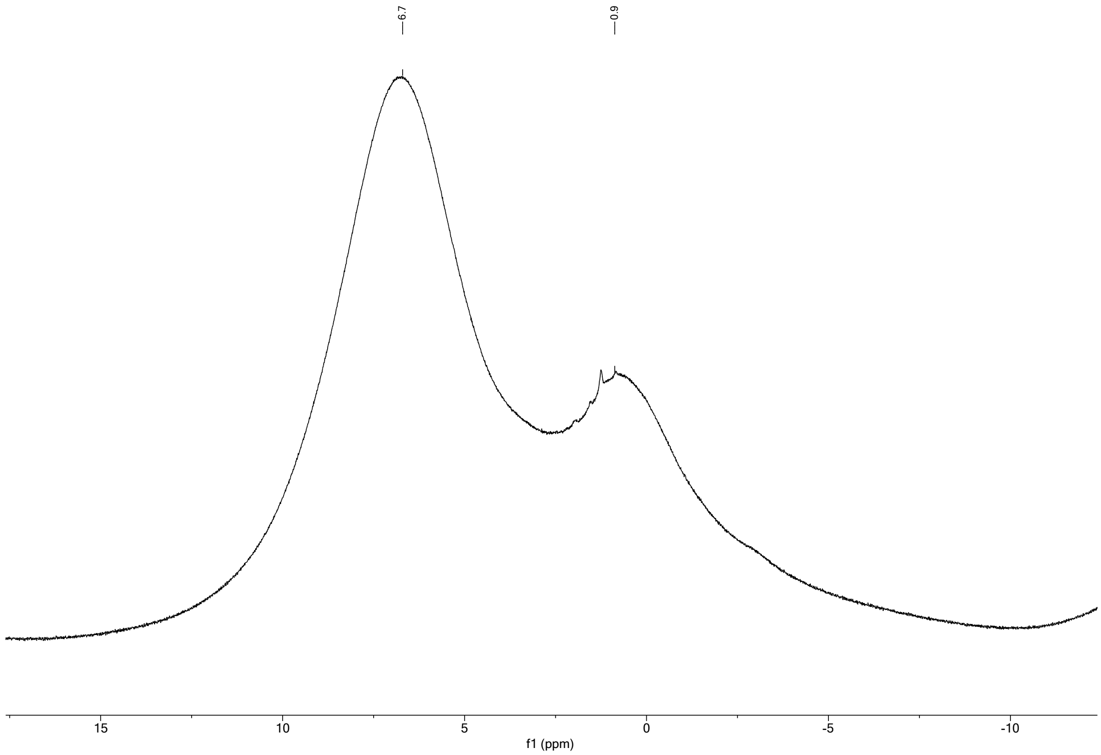

*Figure S 27: ${}^{1}H$ ssNMR MAS spectrum of **m₀-polymer**.*

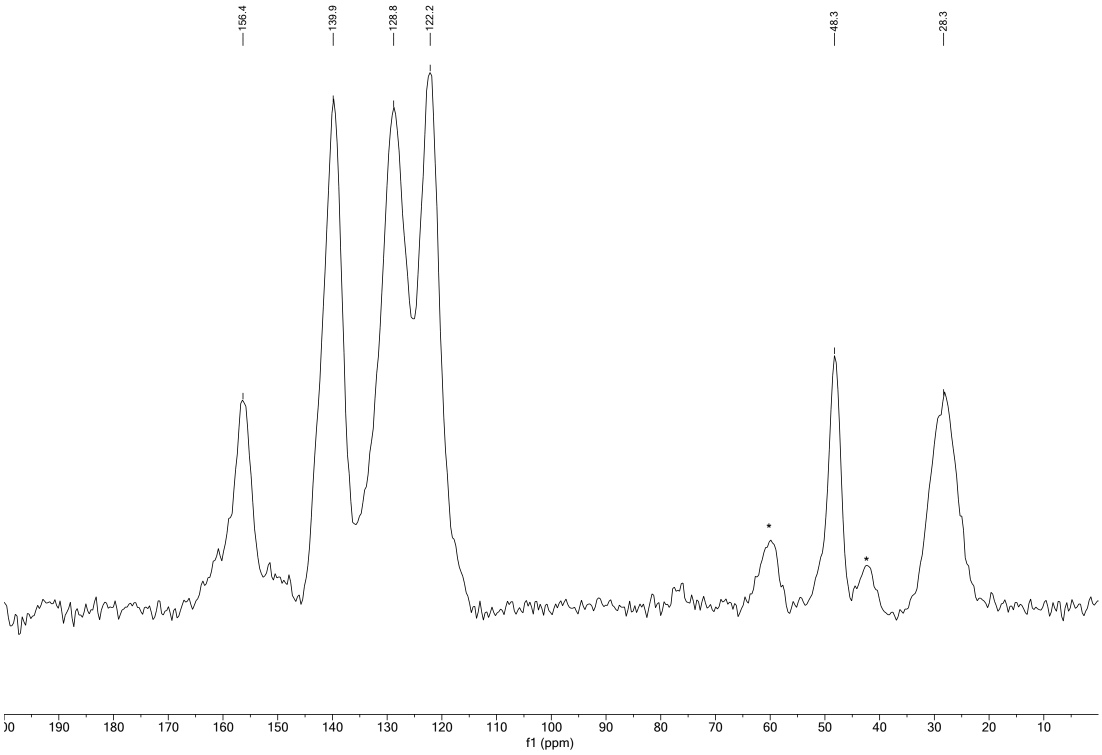

*Figure S 28: ${}^{13}C$ ssNMR CP-MAS spectrum of **m₀-polymer**. Note that the signals at 60 and 42 ppm are spinning sidebands.*

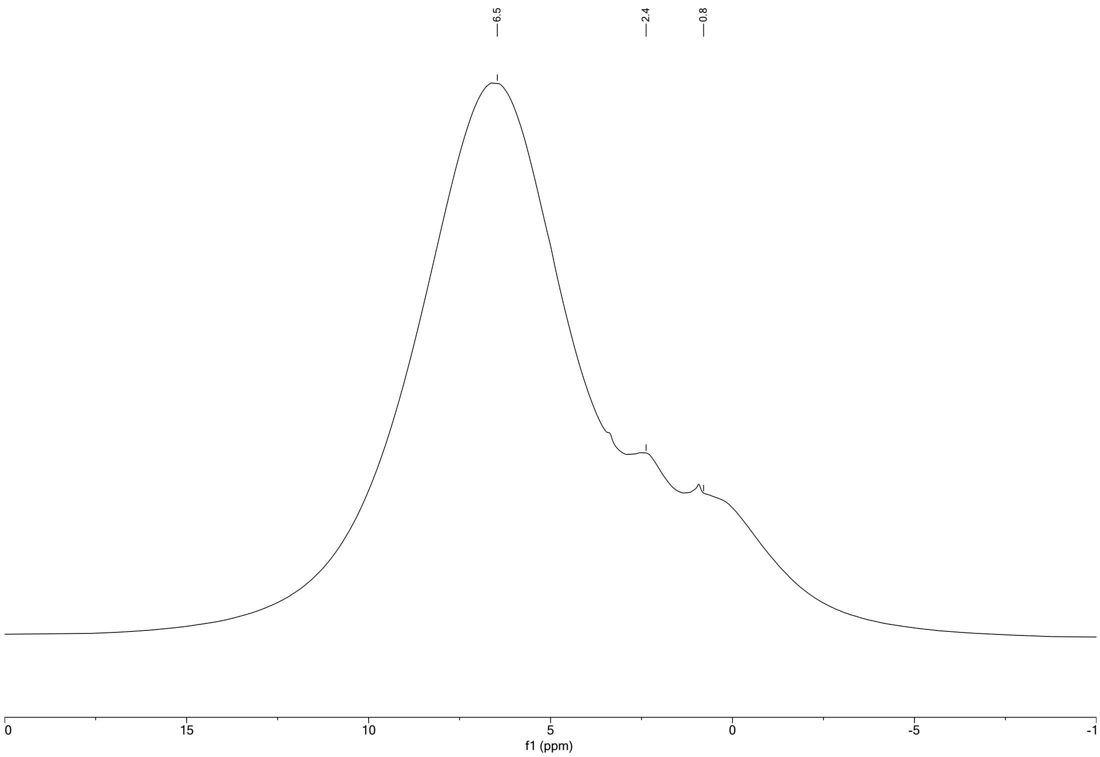

*Figure S 29: ^{1}H ssNMR MAS spectrum of **m_{100}-polymer**.*

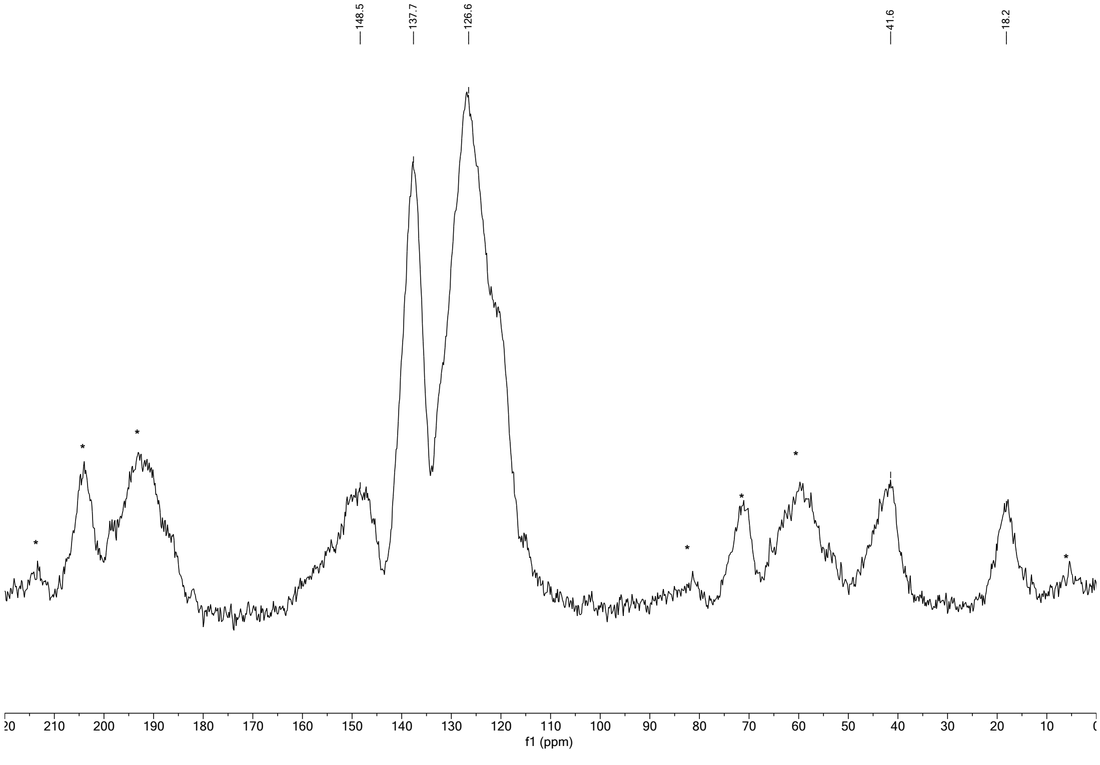

*Figure S 30: ^{13}C ssNMR CP-MAS spectrum of **m_{100}-polymer**. Asterisks indicate spinning site bands.*

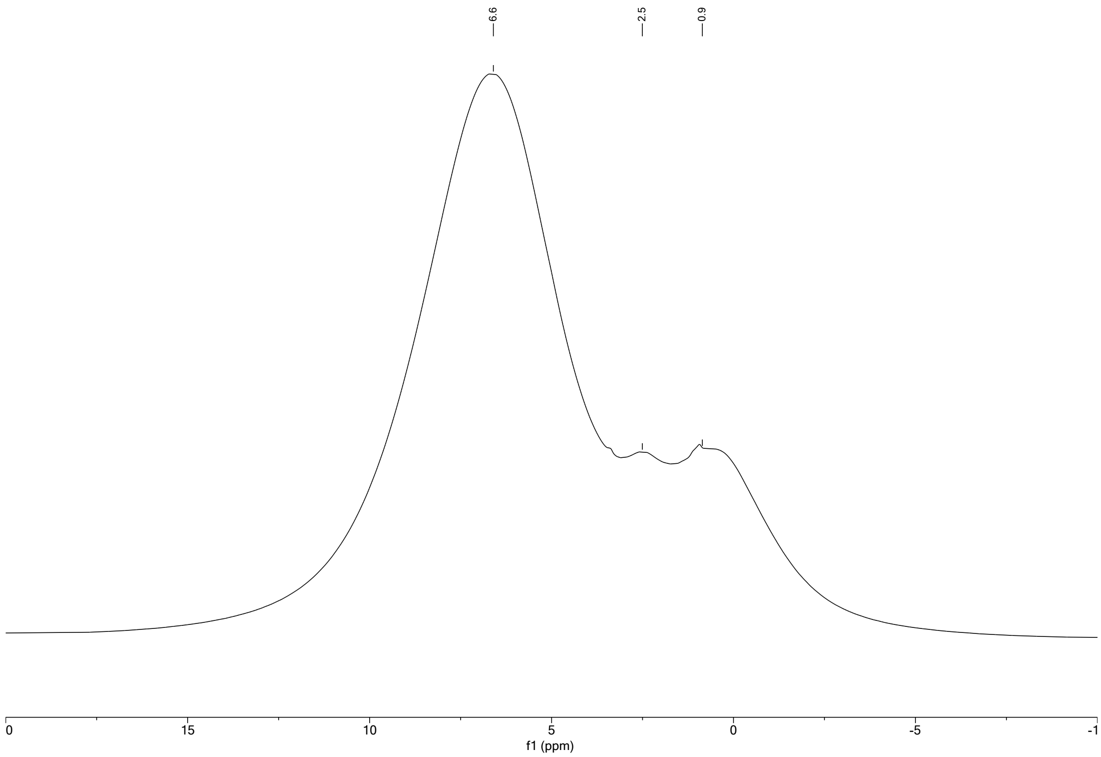

*Figure S 31: ¹H ssNMR MAS spectrum of **m₅₀-polymer**.*

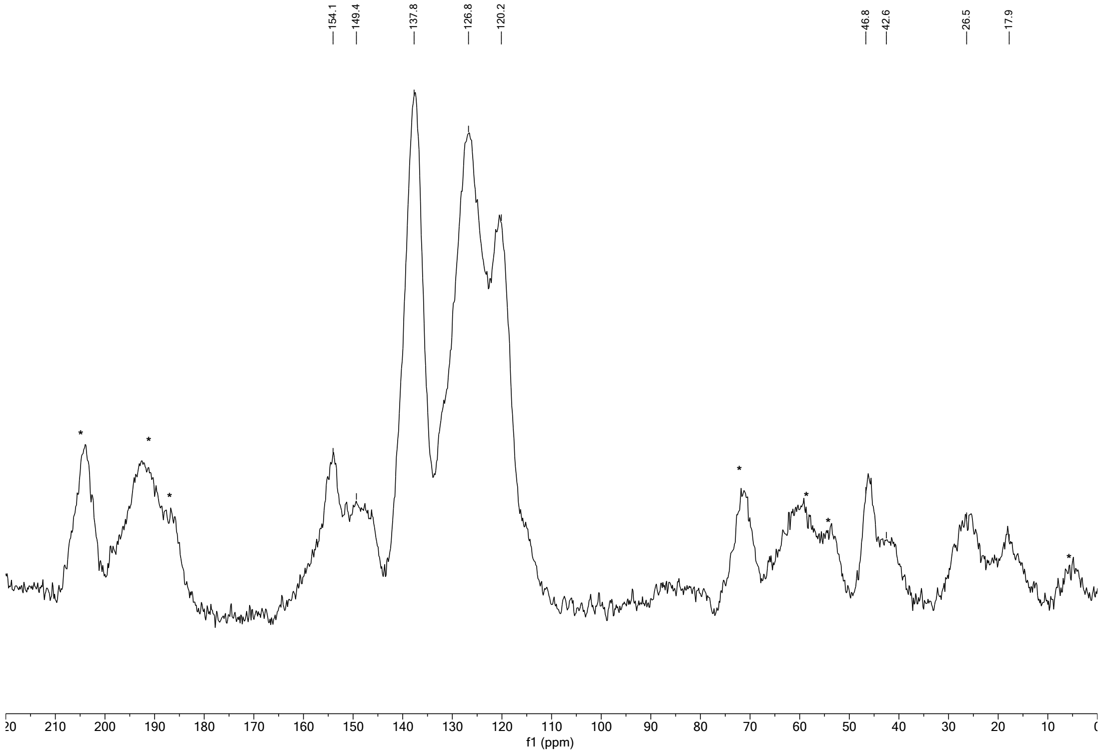

*Figure S 32: ¹³C ssNMR CP-MAS spectrum of **m₅₀-polymer**. Asterisks indicate spinning site bands.*

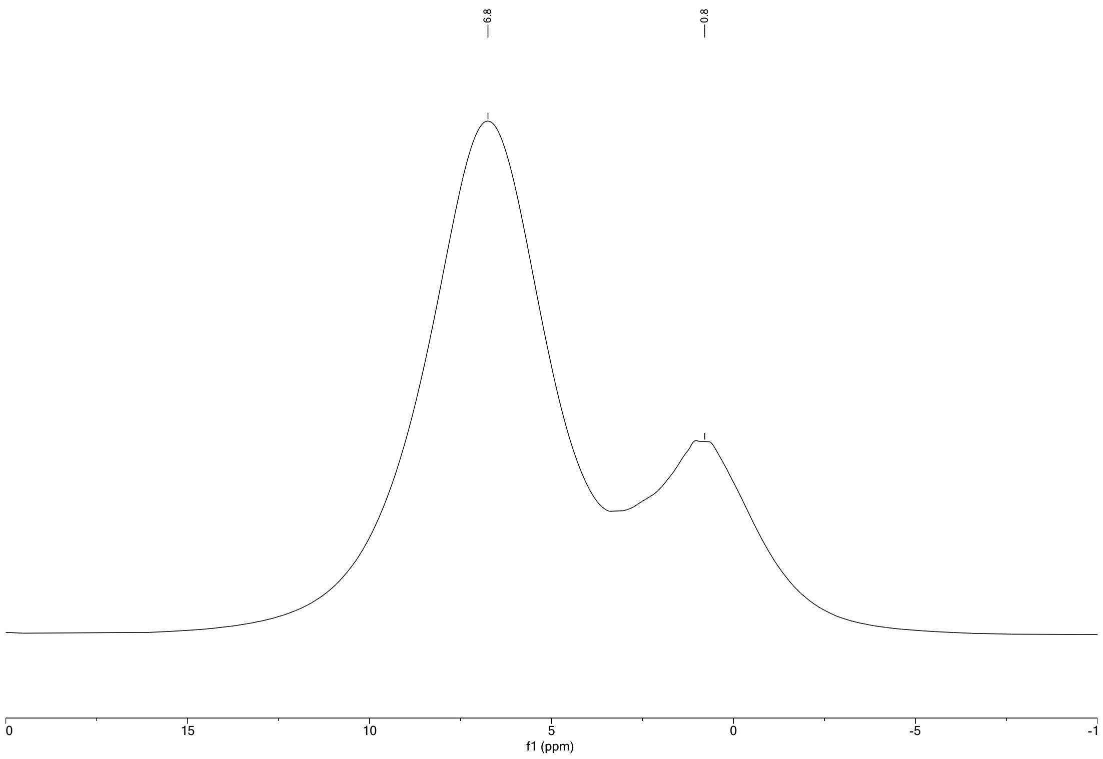

*Figure S 33: ¹H ssNMR MAS spectrum of **m₀-COF** (with 0% amine-motor as building block).*

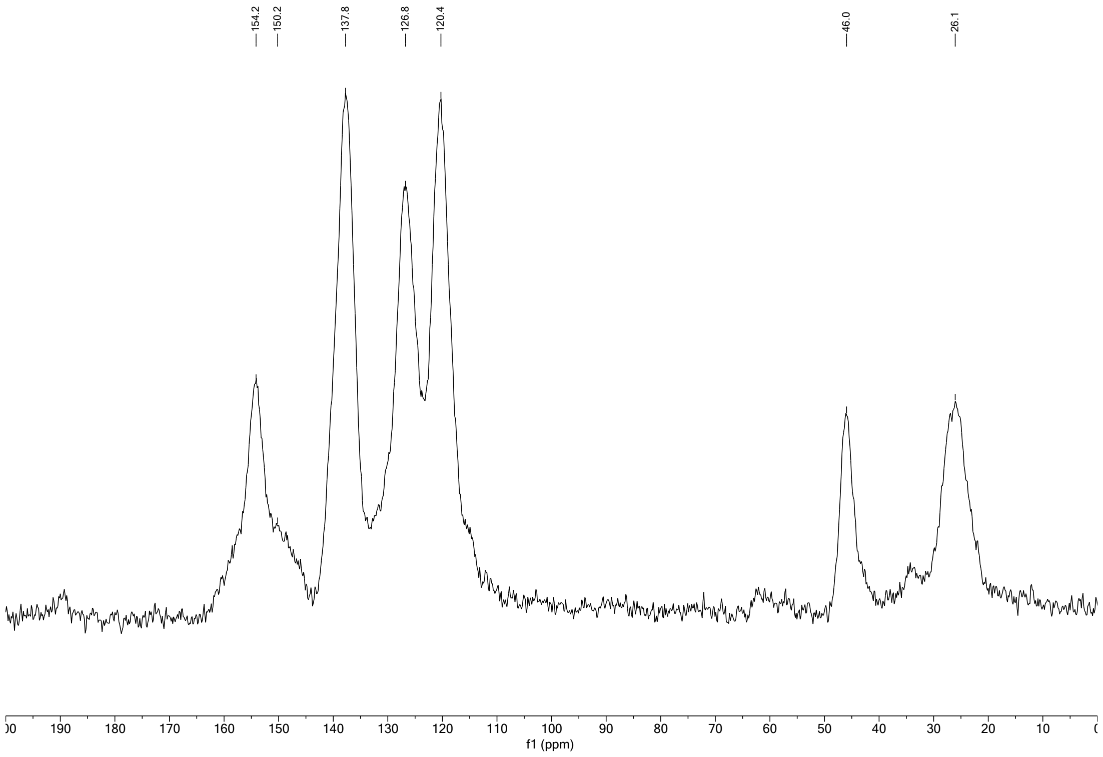

*Figure S 34: ¹³C ssNMR CP-MAS spectrum of **m₀-COF**.*

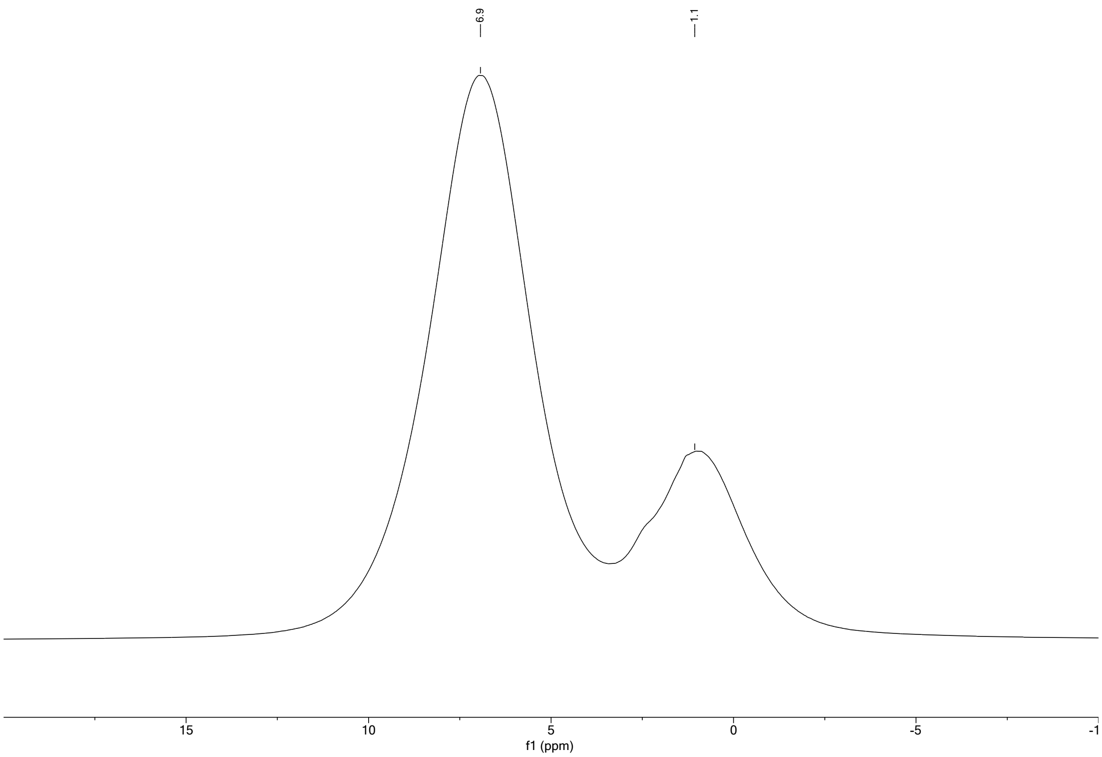

*Figure S 35: ¹H ssNMR MAS spectrum of **m₂₀-COF**.*

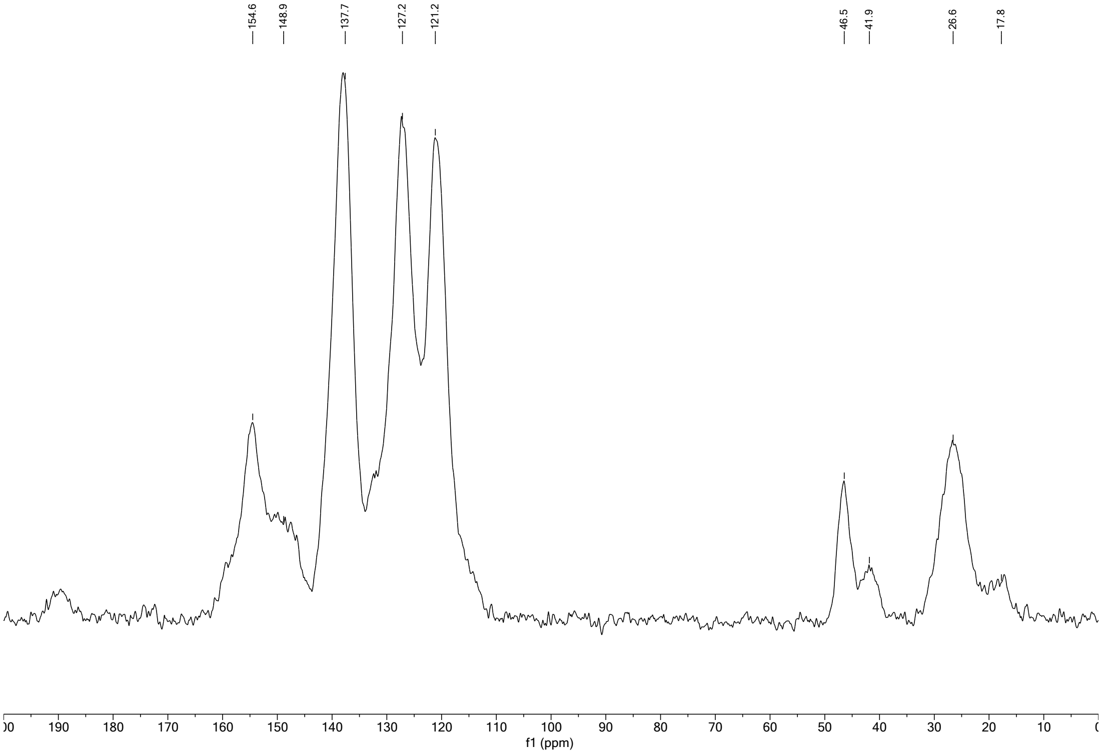

*Figure S 36: ¹³C ssNMR CP-MAS spectrum of **m₂₀-COF**.*

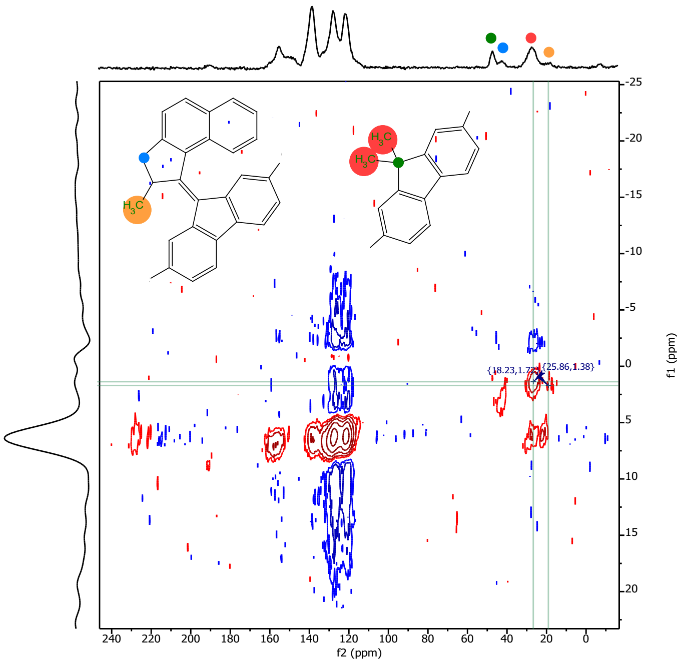

*Figure S 37: $^1H/^{13}C$ HETCOR spectrum of **m_{20}-COF** with distinct signals for spacer (green) and motor (blue) moieties. The correlations highlight that the signal at 47 ppm contains intensity for the quaternary carbon of the spacer (green) as well as the allylic carbon of the motor unit. Thus, these peaks cannot be used for the quantitative integration from the direct excitation experiment. Instead, only the methyl groups (orange, red) were used for this analysis.*

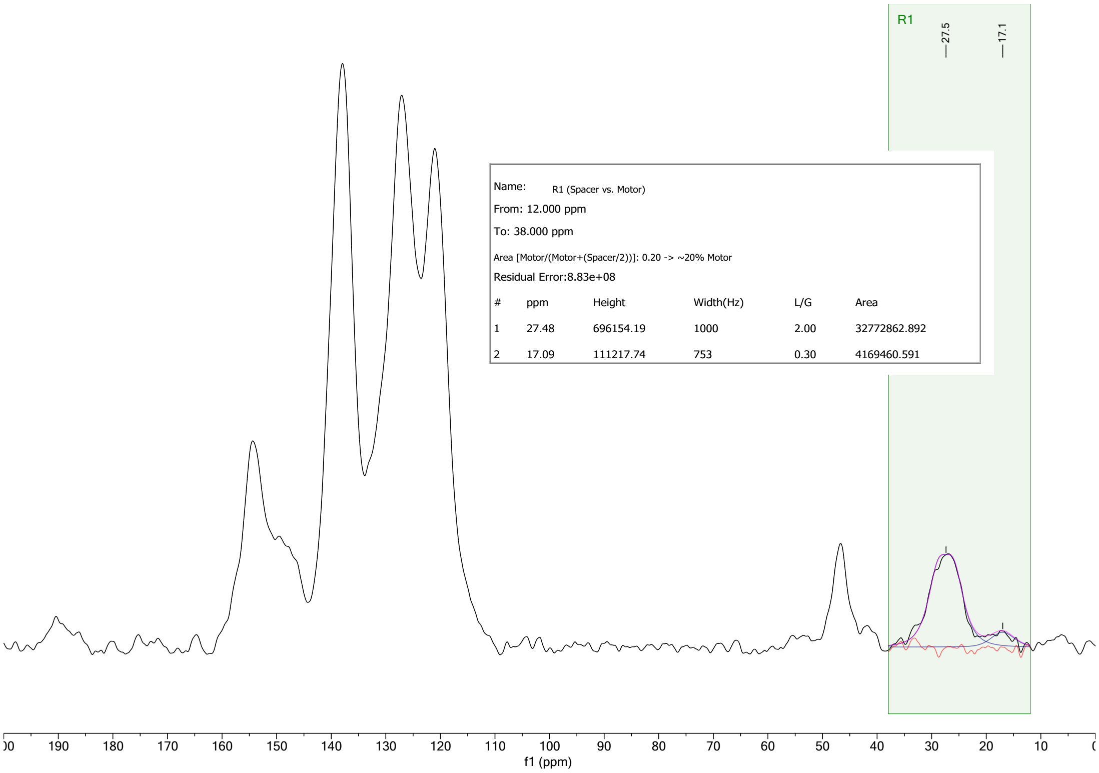

*Figure S 38: ^{13}C ssNMR direct excitation spectrum of **m₂₀-COF**. The signal at 27.5 ppm refers to the two methyl groups of the spacer moiety and the signal at 17.1 ppm to the methyl group of the motor (see Figure S 37), respectively. The relative area of both signals gives a quantitative indication for the motor content in the material of ~20%.*

N_2 Sorption Analysis

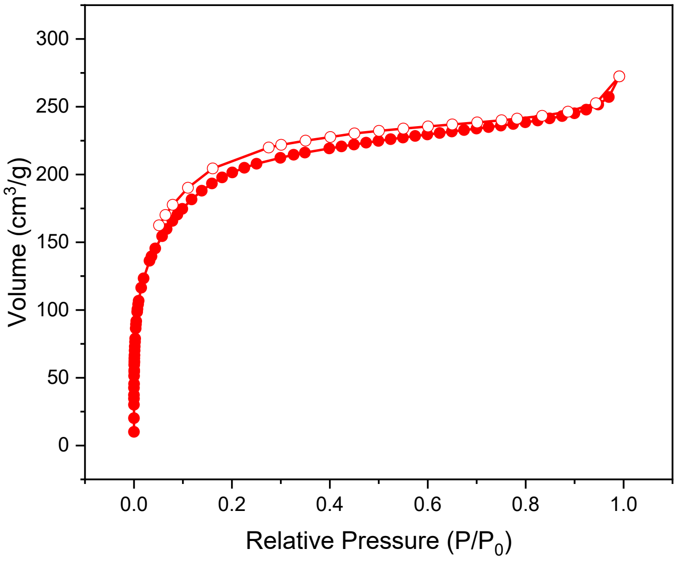

*Figure S 39: N_2 sorption isotherm of **m_0-Polymer**. Filled dots represent data points of the adsorption branch, hollow dots those of the desorption branch, respectively.*

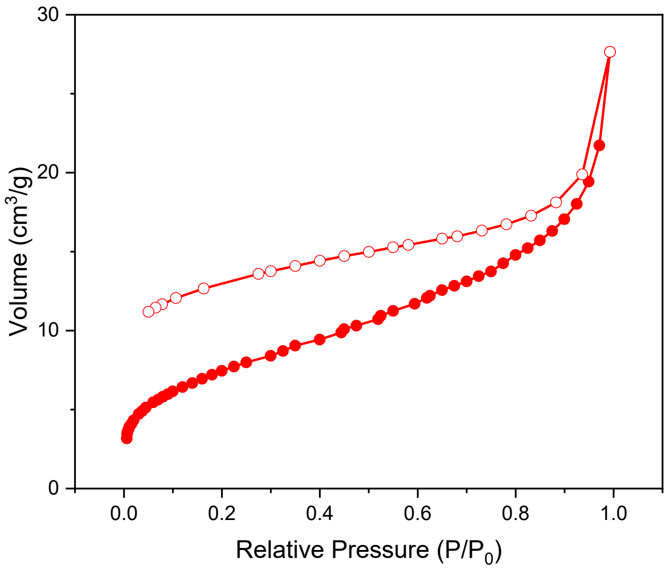

*Figure S 40: N_2 sorption isotherm of **m_{100}-Polymer**. Filled dots represent data points of the adsorption branch, hollow dots those of the desorption branch, respectively.*

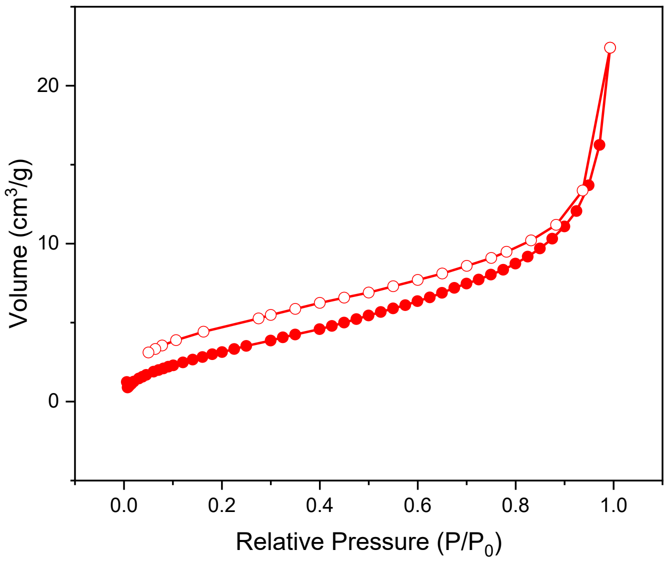

*Figure S 41: N_2 sorption isotherm of **m_{50}-Polymer**. Filled dots represent data points of the adsorption branch, hollow dots those of the desorption branch, respectively.*

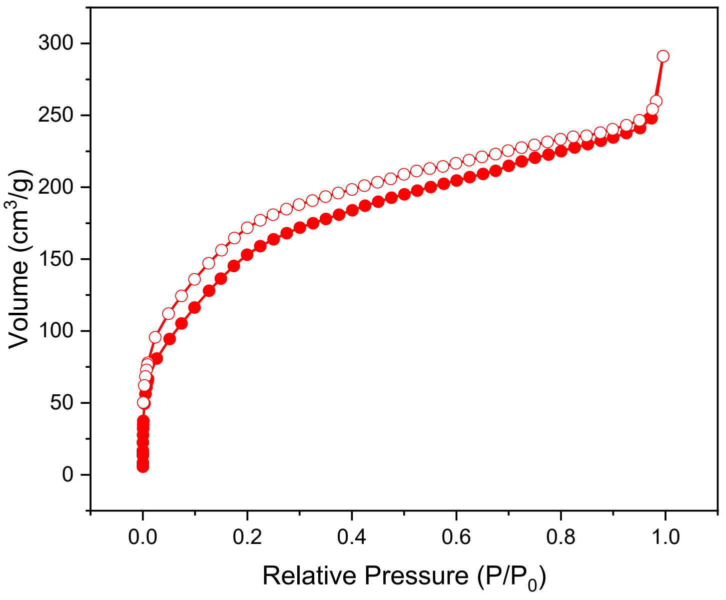

*Figure S 42: N_2 sorption isotherm of **m_{20}-COF**. Filled dots represent data points of the adsorption branch, hollow dots those of the desorption branch, respectively.*

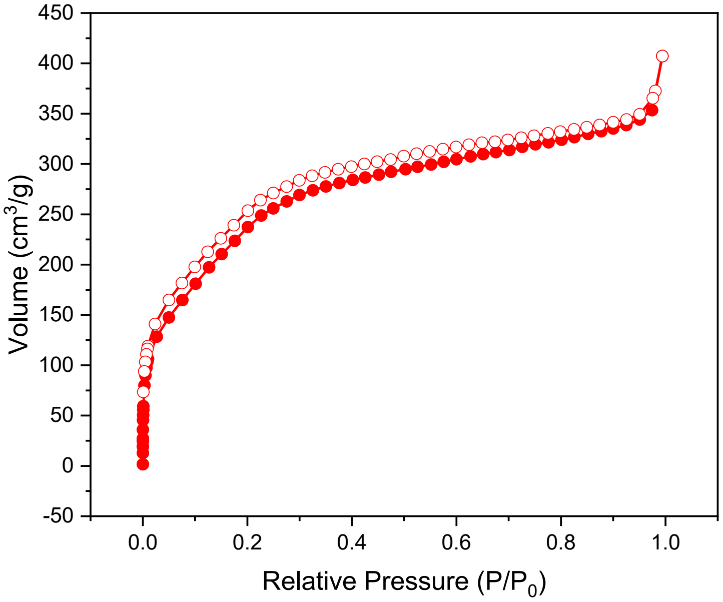

*Figure S 43: N_2 sorption isotherm of **m_0-COF**. Filled dots represent data points of the adsorption branch, hollow dots those of the desorption branch, respectively.*

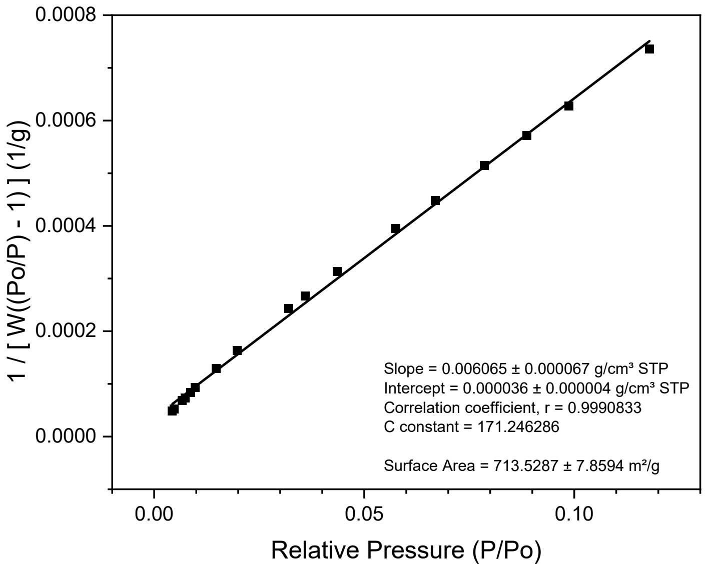

*Figure S 44: BET-Plot of **m_0-Polymer**.*

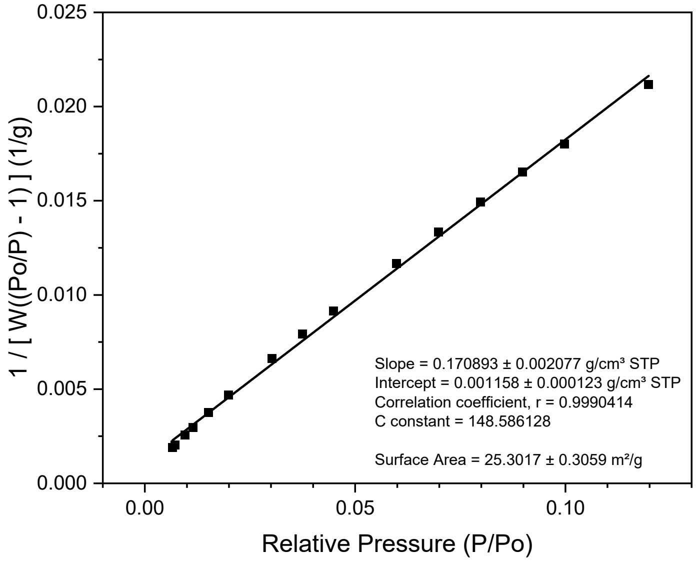

*Figure S 45: BET-Plot of **m_{100}-Polymer**.*

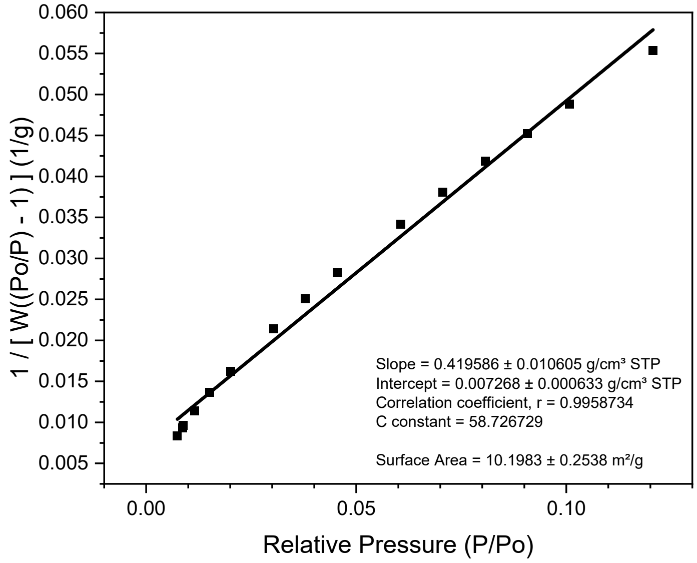

*Figure S 46: BET-Plot of **m_{50}-Polymer**.*

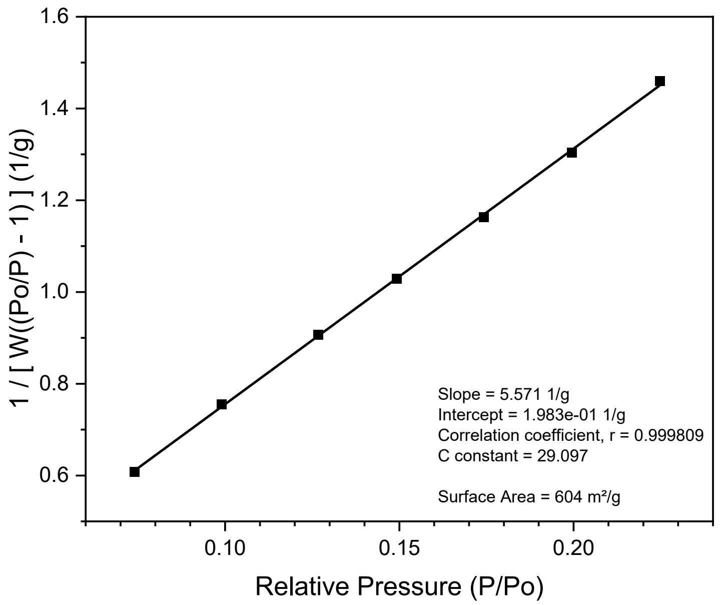

Figure S 47: BET-Plot of ***m_{20}-COF***.

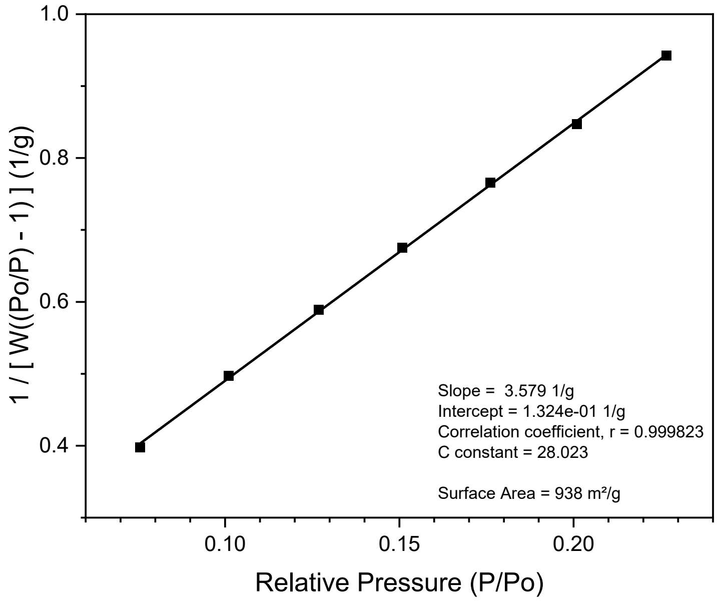

Figure S 48: BET-Plot of ***m_0-COF***.

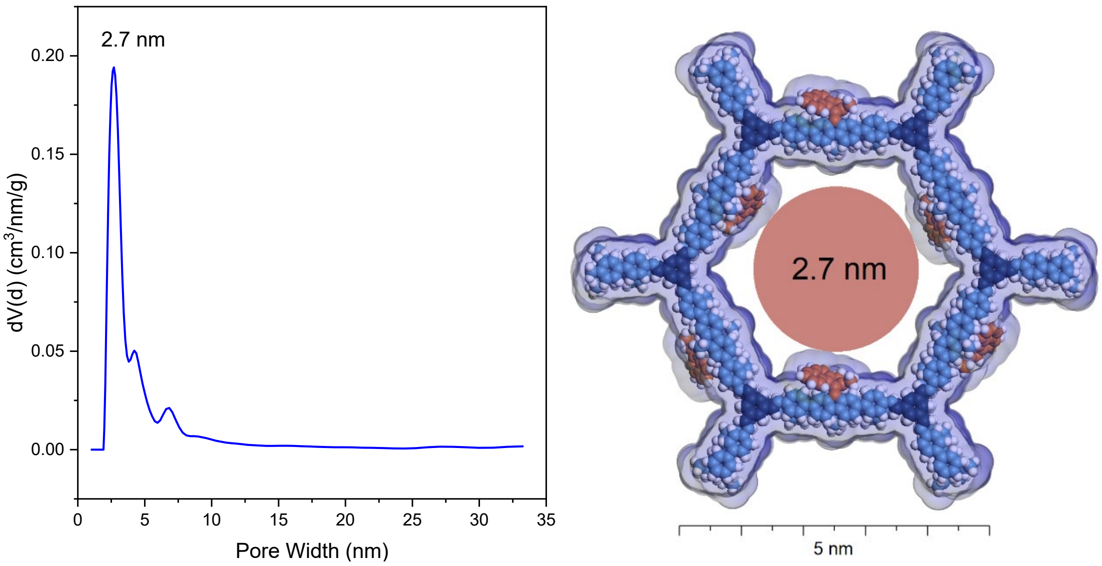

Figure S 49: Experimental pore size distribution in ***m₂₀-COF*** *(left) and estimated minimal pore diameter (N_2 accessible surface) based on an idealized eclipsed stacked structure model (AA-1_EE; see Fig. S10) with equally distributed motor units (represented by a theoretical* ***m₁₀₀-COF****) in Material Studio (right). With smaller motor content, e.g. as found in* ***m₂₀-COF****, the diameter should approach a value closer to* ***m₀-COF*** *(see Fig. S 50).*

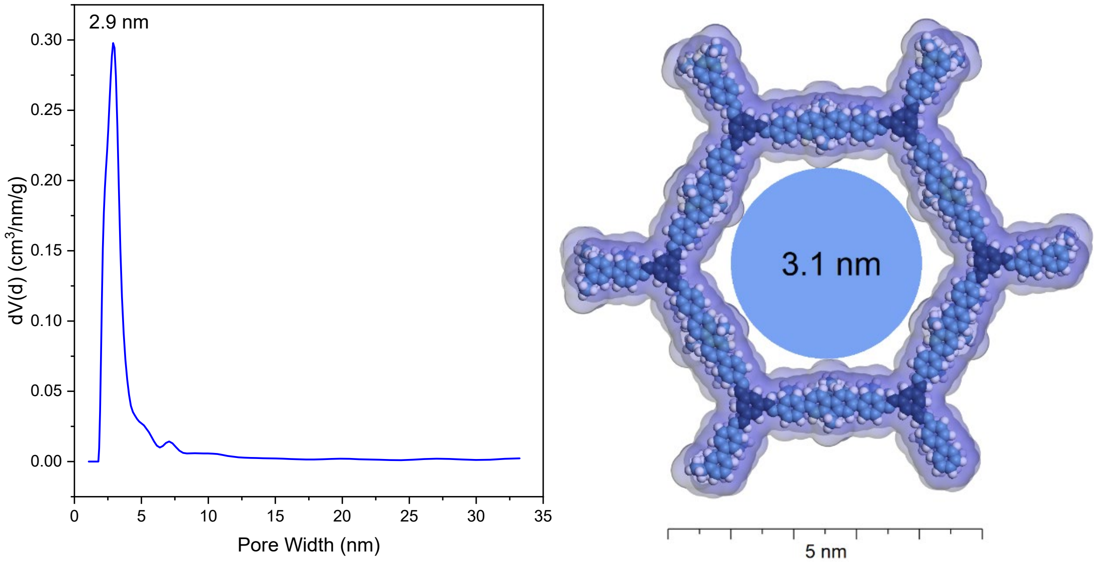

Figure S 50: Experimental pore size distribution in ***m₀-COF*** *(left) and estimated minimal pore diameter (N_2 accessible surface) based on an idealized eclipsed stacked structure model (AA-1_EE; see Fig. S10) in Material Studio (right).*

SEM/TEM Analysis

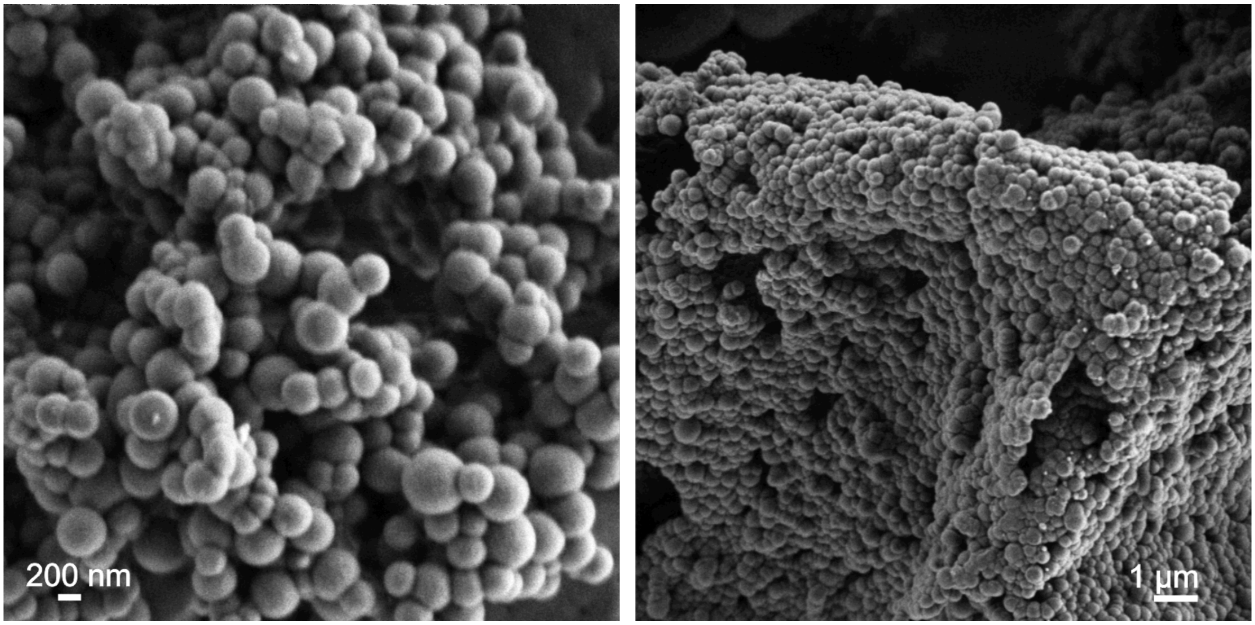

Figure S 51: SEM images of ***m$_{20}$-COF*** *showing intergrown spherical particles.*

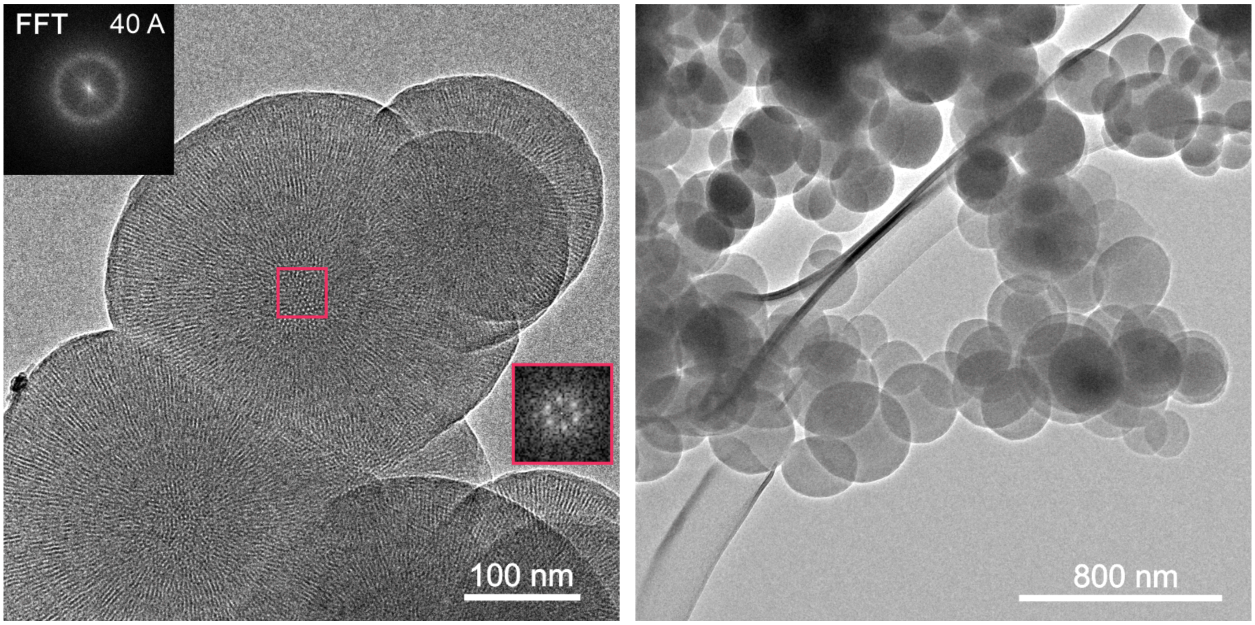

Figure S 52: TEM images of ***m$_{20}$-COF*** *showing crystalline, intergrown spherical particles with ~150-350 nm diameter. The FFT (red) shows a hexagonal pattern, proving the structure model.*

DFT Calculations

General methods

All the structures subject to the analysis were pre-optimized using the semiempirical GFN2-xTB level, as implemented in the xTB software.[9] Compounds **1** and **2** were optimized considering the states involved in the thermal step of a molecular motor (stable, **S**; metastable, **MS**; THI transition state, **TS**) and the different orientations of the aryl rings attached to the stator (see Figure S 55 and Figure S 56). For each molecule, four conformers were considered, from which the rotational cycle could start:

-1: left aryl backwards/right aryl front

-2: both aryls front

-3: left aryl front/right aryl backwards

-4: both aryls backwards

The conformers of each compound were enumerated accordingly (e.g. **1-S2** for the second conformer of the stable state of molecule **1**) and their structures reoptimized with DFT at ωB97X-D/def2-SVP level, with the Gaussian 16, Rev B.01 software package. All stationary points were confirmed to be such due to the number of imaginary frequencies obtained after the Hessian calculation (0 for minima, 1 for transition states). The Gibbs free energy correction was applied to all the electronic energies. The energy distribution of the metastable states was used to calculate a Boltzmann distribution to be applied to the simulated THI reaction. The results gave a simulated $\Delta G^{\ddagger}$ of 97.7 kJ/mol for **1** and 99.7 kJ/mol for **2**. The choice of functional and basis set affords an error of ca. 10 kJ/mol compared to the experimental barrier, correctly retrieving the relative order of reactivity (**1** slightly more reactive than **2**). The UV-Vis spectra of stable and unstable states were calculated at the TD-ωB97X-D/def2-TZVP level over the first 15 singlet transitions on the geometries calculated in the gas phase. The SMD implicit solvent method (for MeCN) was applied. The broad absorption band centered around 350 nm of the stable state and the appearance of a more prominent transition around 450 nm were correctly modelled. All xyz coordinates are reported as separate files, while the simulated UV-Vis for stable and unstable compounds are reported below (left, gas phase, right MeCN).

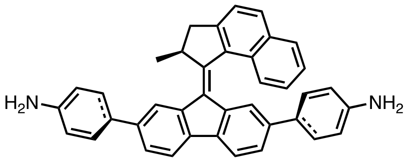

1-1

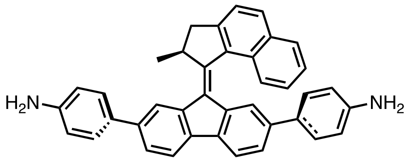

1-2

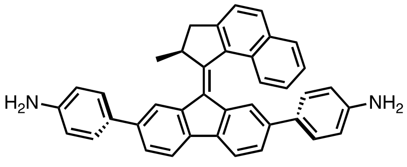

1-3

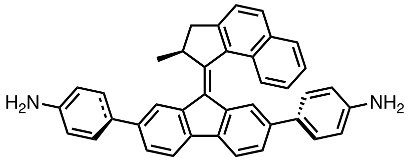

1-4

*Figure S 55: Conformers of motor **1** considered in the DFT studies.*

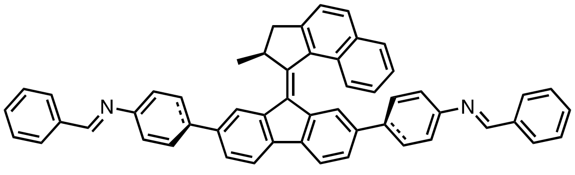

2-1

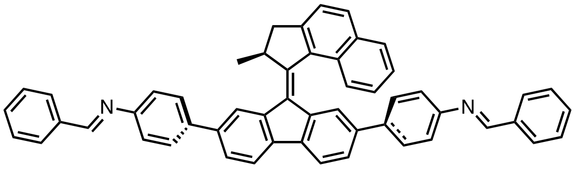

2-2

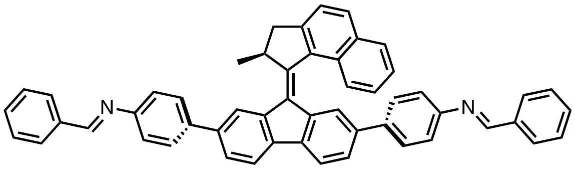

2-3

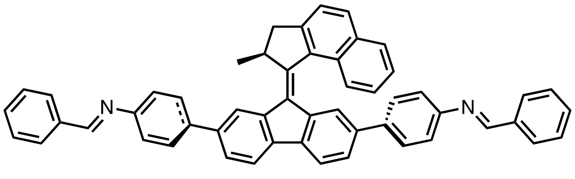

2-4

*Figure S 56: Conformers of motor **2** considered in the DFT studies.*

*Table S 2: Energy Barriers and half-lives of conformers of motor **1**.*

Conformers	Energy Barrier [kJ/mol]	Energy Barrier [kcal/mol]	k [s-1]	$t_{1/2}$ [s]	$t_{1/2}$ [min]
**MS1-TS1**	88.21	21.08	2.19E-03	317	5
**MS3-TS3**	99.04	23.67	2.77E-05	25029	417
**MS2-TS2**	98.21	23.47	3.87E-05	17910	298
**MS4-TS4**	91.14	21.78	6.71E-04	1034	17

*Table S 3: Energy Barriers and half-lives of conformers of motor **2**.*

Conformers	Energy Barrier [kJ/mol]	Energy Barrier [kcal/mol]	k [s-1]	$t_{1/2}$ [s]	$t_{1/2}$ [min]
**MS1-TS1**	90.25	21.57	9.60E-04	722	12
**MS3-TS3**	101.38	24.23	1.08E-05	64310	1072
**MS2-TS2**	99.54	23.79	2.27E-05	30576	510
**MS4-TS4**	90.47	21.62	8.79E-04	788	13

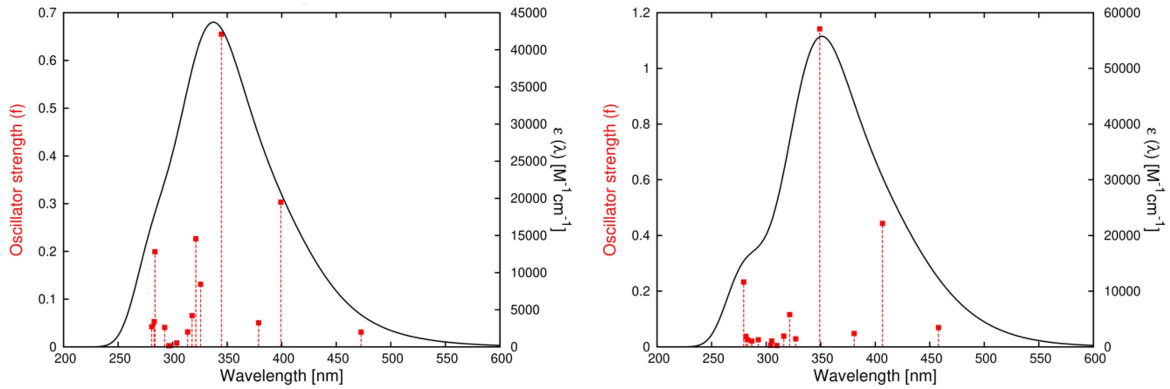

*Figure S 57: Simulated UV-Vis absorption spectra for compound **1-S1** in gas phase (left) and acetonitrile (right).*

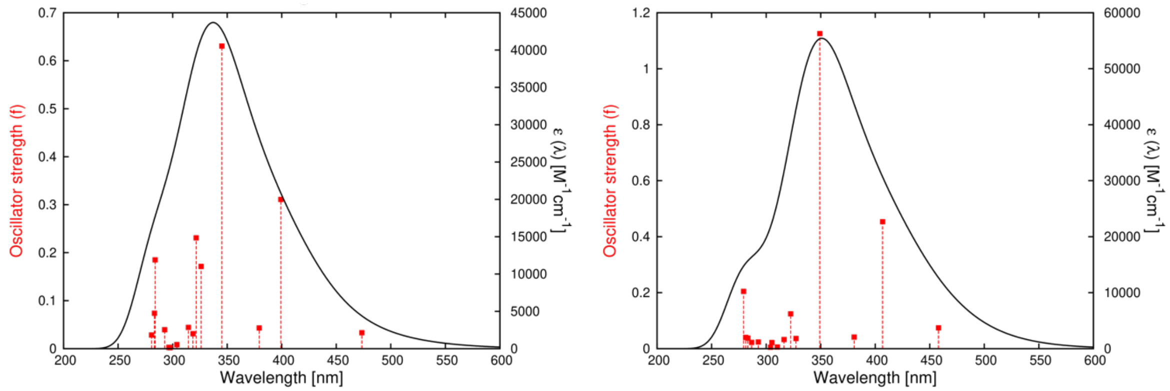

*Figure S 58: Simulated UV-Vis absorption spectra for compound **1-S2** in gas phase (left) and acetonitrile (right).*

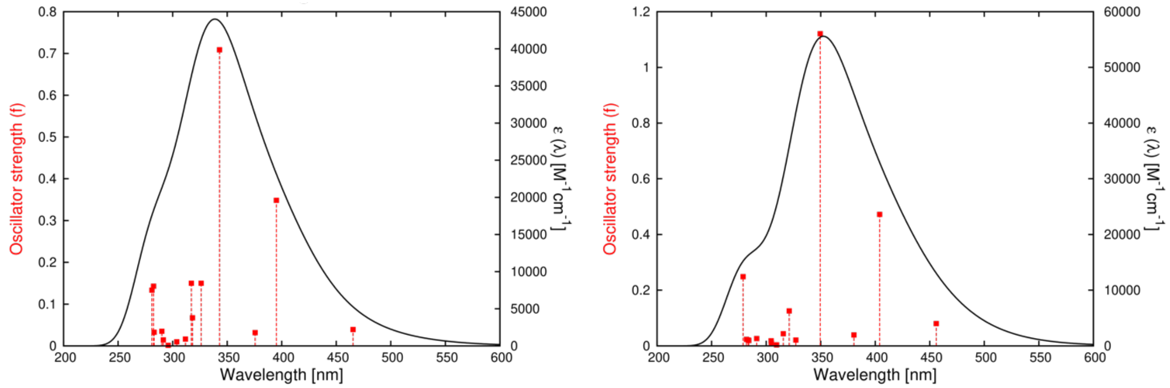

*Figure S 59: Simulated UV-Vis absorption spectra for compound **1-S3** in gas phase (left) and acetonitrile (right).*

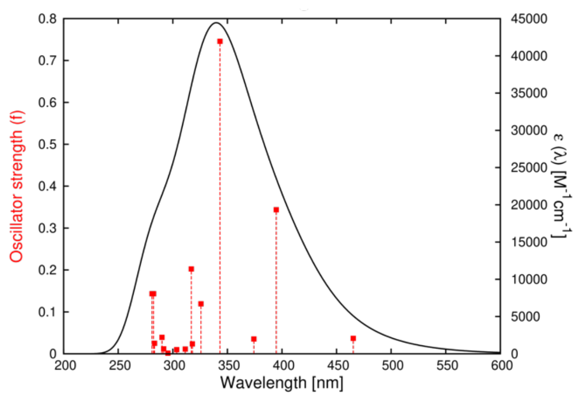

*Figure S 60: Simulated UV-Vis absorption spectra for compound **1-S4** in gas phase (left) and acetonitrile (right).*

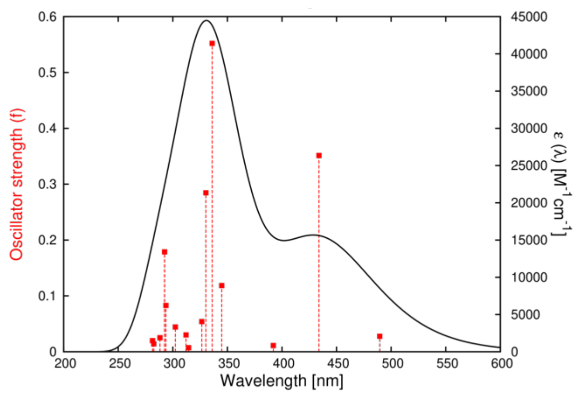

*Figure S 61: Simulated UV-Vis absorption spectra for compound **1-MS1** in gas phase (left) and acetonitrile (right).*

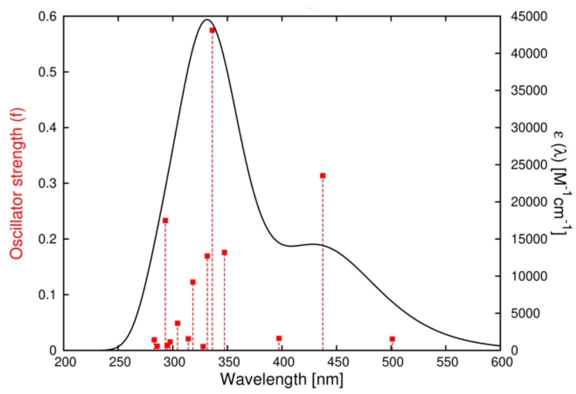

*Figure S 62: Simulated UV-Vis absorption spectra for compound **1-MS2** in gas phase (left) and acetonitrile (right).*

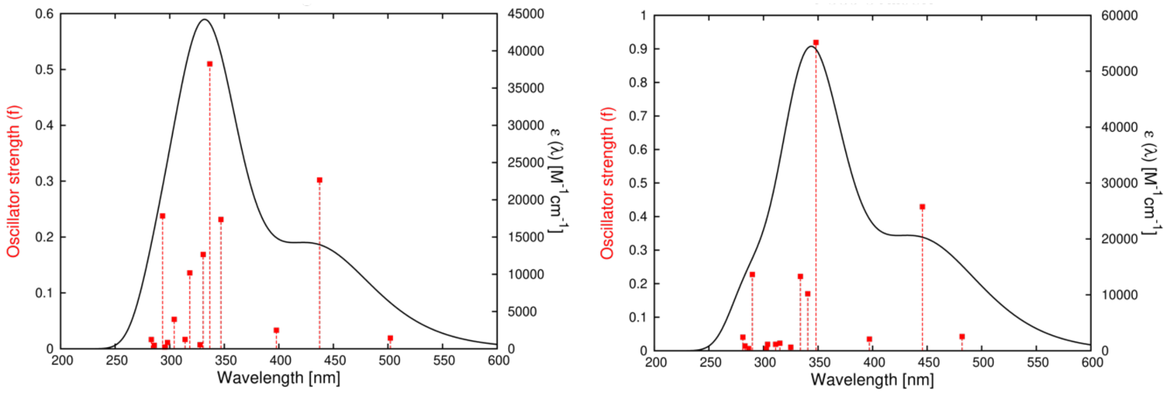

*Figure S 63: Simulated UV-Vis absorption spectra for compound **1-MS3** in gas phase (left) and acetonitrile (right).*

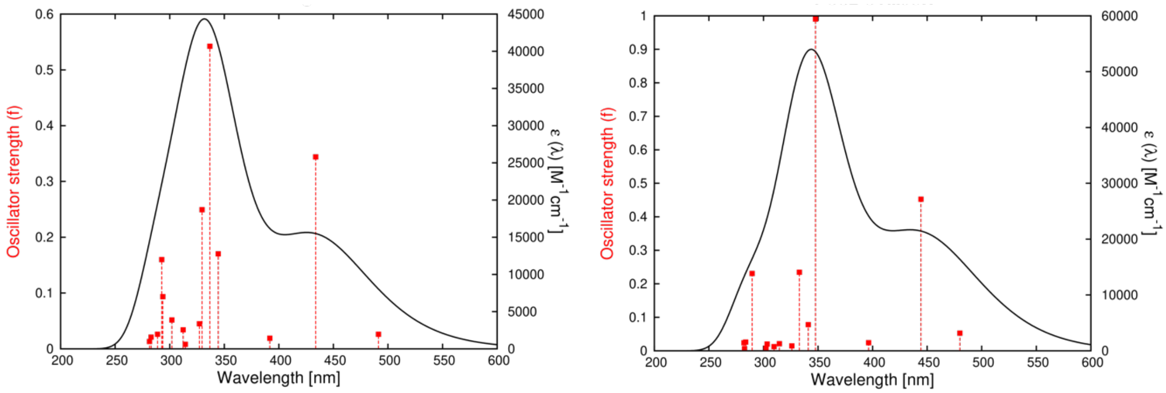

*Figure S 64: Simulated UV-Vis absorption spectra for compound **1-MS4** in gas phase (left) and acetonitrile (right).*

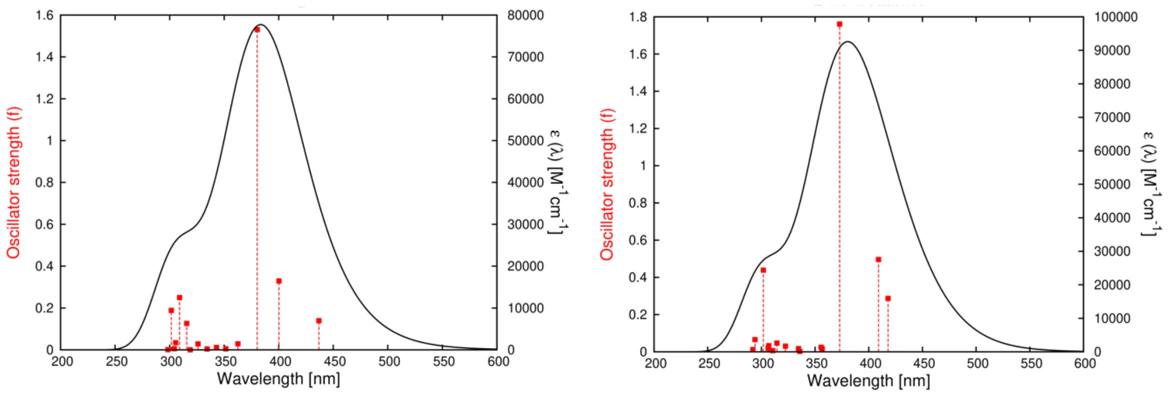

*Figure S 65: Simulated UV-Vis absorption spectra for compound **2-S1** in gas phase (left) and acetonitrile (right).*

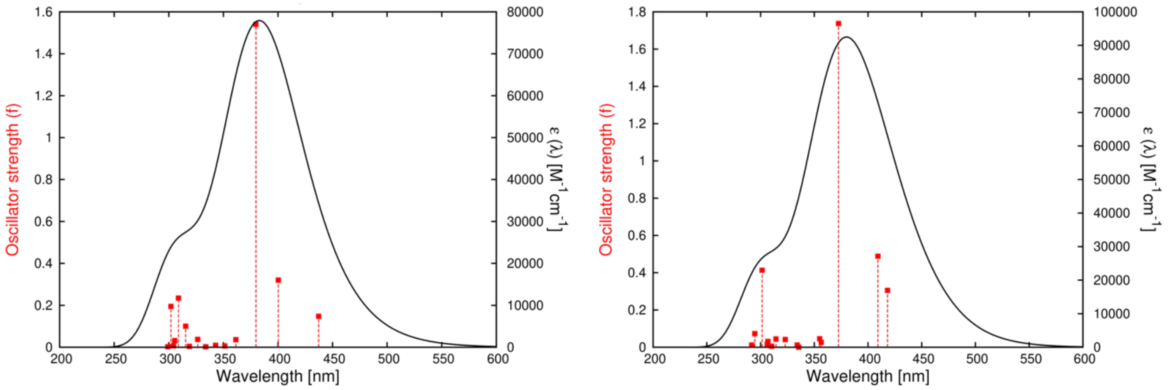

*Figure S 66: Simulated UV-Vis absorption spectra for compound **2-S2** in gas phase (left) and acetonitrile (right).*

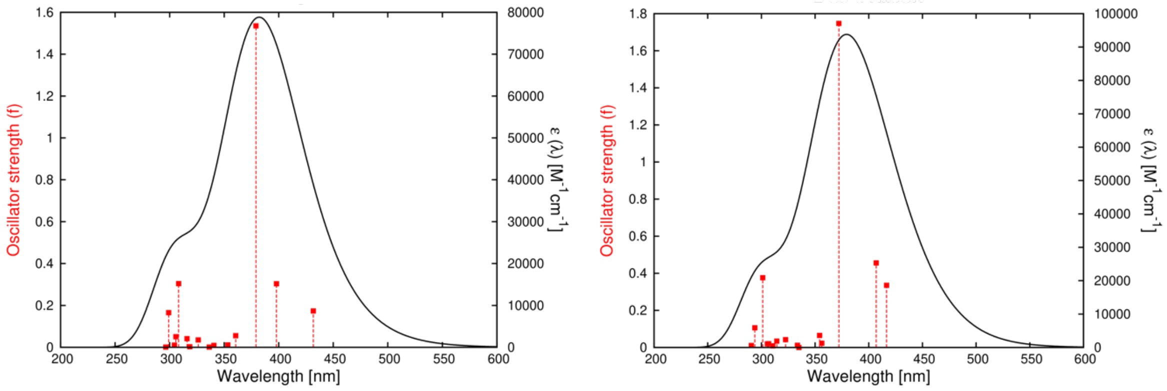

*Figure S 67: Simulated UV-Vis absorption spectra for compound **2-S3** in gas phase (left) and acetonitrile (right).*

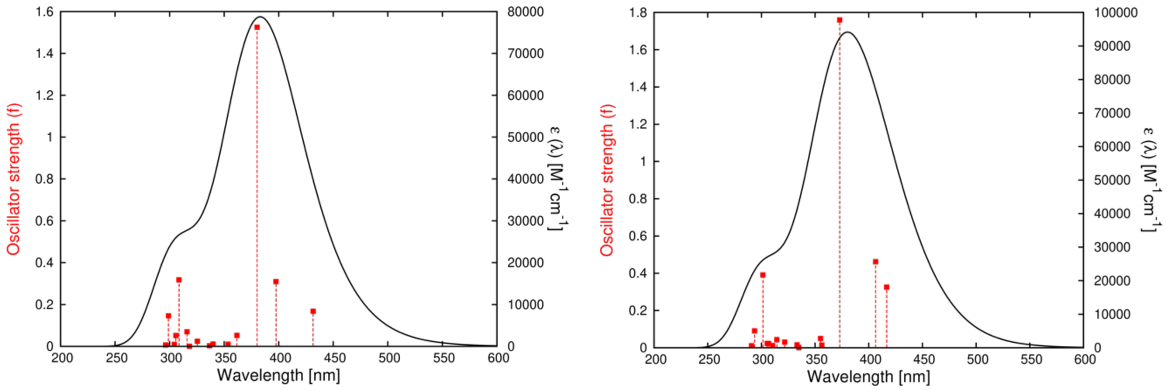

*Figure S 68: Simulated UV-Vis absorption spectra for compound **2-S4** in gas phase (left) and acetonitrile (right).*

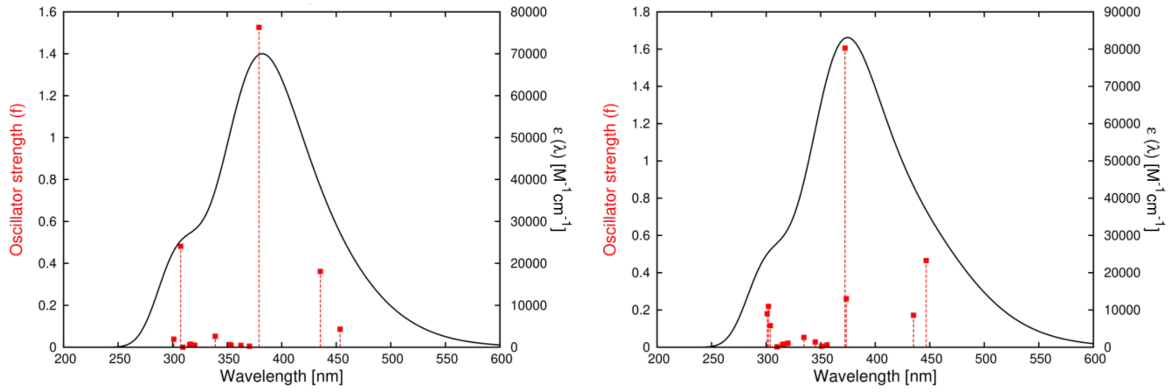

*Figure S 69: Simulated UV-Vis absorption spectra for compound **2-MS1** in gas phase (left) and acetonitrile (right).*

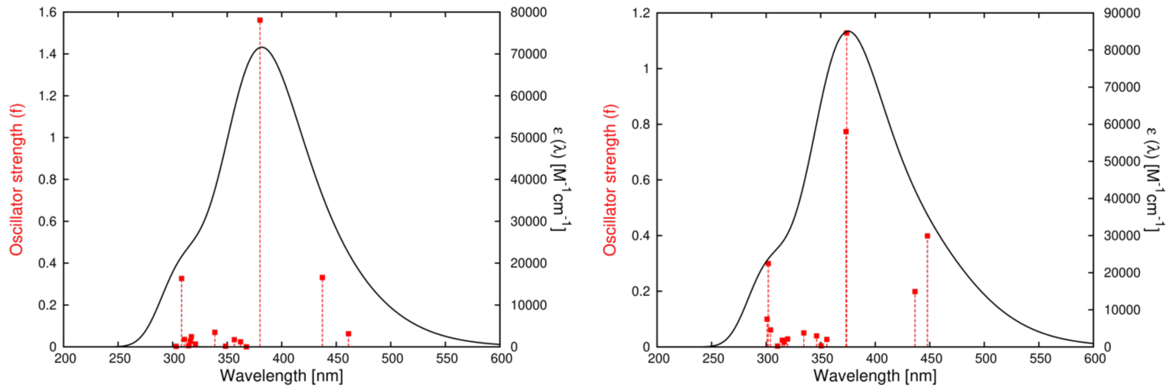

*Figure S 70: Simulated UV-Vis absorption spectra for compound **2-MS2** in gas phase (left) and acetonitrile (right).*

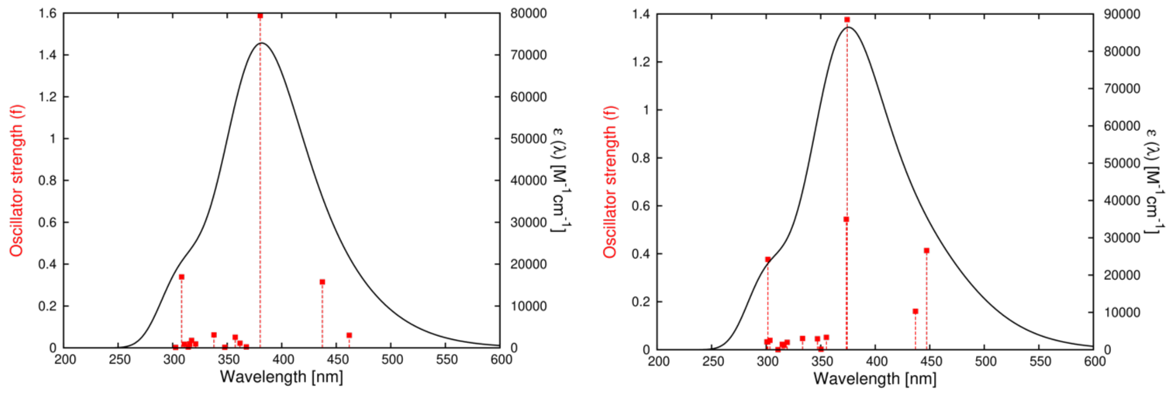

*Figure S 71: Simulated UV-Vis absorption spectra for compound **2-MS3** in gas phase (left) and acetonitrile (right).*

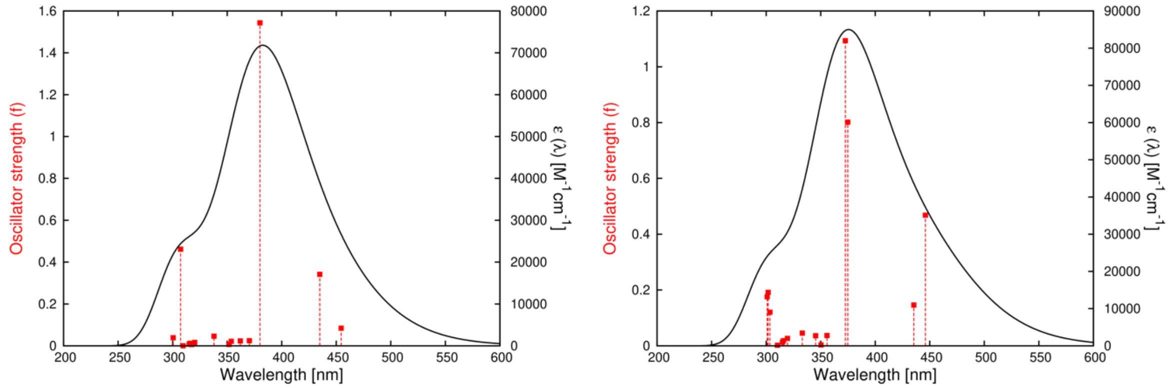

*Figure S 72: Simulated UV-Vis absorption spectra for compound **2-MS4** in gas phase (left) and acetonitrile (right).*

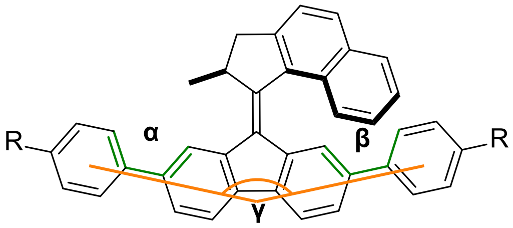

1: R = NH_2
2: R = N=CH-Ph

Figure S 73: Measured angles from optimized structures in DFT of 1 and 2. The dihedral angles marked in green are summarized in Table S 4. The angle marked in orange indicates the angle in which the molecule is bend out of plane.

*Table S 4: Summary of dihedral angles (as indicated in green in Figure S 73) for stable, metastable and transition states of motor **1** and **2** and bending angles (marked in orange in Figure S 73).*

Conformer	dihedral α [°]	dihedral β [°]	bending γ [°]	Conformer	dihedral α [°]	dihedral β [°]	bending γ [°]
**1-S1**	39	35	164	**2-S1**	39	35	161
**1-MS1**	38	40	162	**2-MS1**	38	40	162
**1-TS1**	40	38	152	**2-TS1**	41	39	152
**1-S3**	-39	-36	164	**2-S3**	-40	-38	159
**1-MS3**	-36	-39	161	**2-MS3**	-37	-40	162
**1-TS3**	-25	-40	150	**2-TS3**	-27	-41	151
**1-S2**	-39	35	165	**2-S2**	-40	35	160
**1-MS2**	-36	40	162	**2-MS2**	-37	40	162
**1-TS2**	-26	38	151	**2-TS2**	-27	39	149
**1-S4**	39	-36	167	**2-S4**	39	-37	164
**1-MS4**	38	-39	161	**2-MS4**	38	-40	161
**1-TS4**	-40	40	149	**2-TS4**	41	-41	152

DIFFaX Simulations

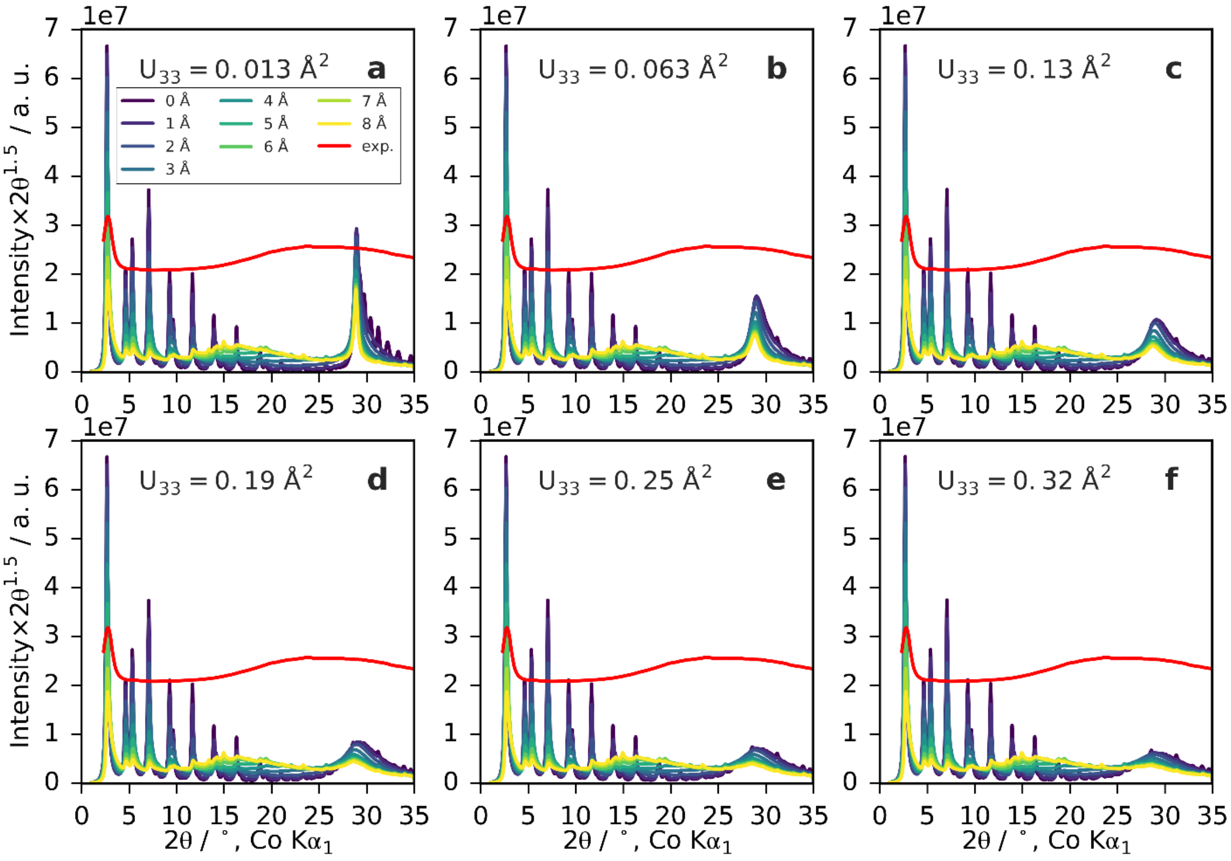

*Figure S 53: Diffraction patterns of turbostratically disordered **m₂₀-COF** were simulated using the program DIFFax[10]. Layers were allowed to shift with equal probability in six directions: [100], [110], [010], [-100], [-1-10], [0-10]. Then, the magnitude of the shifts were increased incrementally, and the simulations were repeated with different values for the interlayer atomic displacement parameter U_{33} to simulate decreasing coherence between the layers. To keep the complexity of the simulations manageable, only a single layer orientation was considered (e.g., to properly account for antiparallel stacking would require two alternating orientations), and the motor units were also not considered. We found that a combination of both interlayer offsets and increased U_{33} are required to satisfactorily destroy the interlayer diffraction effects as seen in the experimental data. Increased offsets up to about 8 Å and very high U_{33} values led to substantial broadening and coalescence of diffuse intensities around 15-35° 2θ. Shift magnitudes larger than 8 Å led to the re-emergence of other diffraction peaks that are not observed. It is difficult to quantify the limits of these effects, as other factors such as distributions of stacking magnitude, motor units, conformational disorder, and different relative orientations (i.e., antiparallel versus parallel stacking) can be expected to have further complicating effects, and possibly are also necessary to obtain the "x-ray amorphous"-looking patterns seen experimentally.*

References

1. Chupas P. J et. al. Rapid acquisition pair distribution function analysis (RA-PDF). *J. Appl. Crystallogr.* **36**, 1342–1347 (2003).

2. Ashiotis G. et. al. The fast azimuthal integration Python library: pyFAI. *J. Appl. Crystallogr.* **48**, 510–519 (2015).

3. https://xpdacq.github.io/xpdtools/index.html

4. Juhás P., Davis T., Farrow C. L., Billinge S. J. L. PDFgetX3: A rapid and highly automatable program for processing powder diffraction data into total scattering pair distribution functions. *J. Appl. Crystallogr.* **46**, 560–566 (2013).

5. Yang X., Juhás P., Farrow C. L., Billinge S. J. L. xPDFsuite: an end-to-end software solution for high throughput pair distribution function transformation, visualization and analysis. *arXiv* 1402.3163 (2015).

6. Farrow C. L. et. al. PDFfit2 and PDFgui: Computer programs for studying nanostructure in crystals. *J. Phys: Condens. Mat.* **19**, 335219 (2007).

7. Danowski W. et. al. Unidirectional rotary motion in a metal-organic framework. *Nat. Nanotechnol.* **14**, 488-494 (2019).

8. Gobel D., Clamor N., Lork E., Nachtsheim B. J. Aerobic C(sp(2))-H Hydroxylations of 2-Aryloxazolines: Fast Access to Excited-State Intramolecular Proton Transfer (ESIPT)-Based Luminophores. *Org. Lett.* **21**, 5373-5377 (2019).

9. Pracht P., Bohle F., Grimme S. Automated exploration of the low-energy chemical space with fast quantum chemical methods. *Phys. Chem. Chem. Phys.* **22**, 7169-7192 (2020).

10. Treacy M. M., Newsam J. M., Deem M. W. A general recursion method for calculating diffracted intensities from crystals containing planar faults. *Proc. R. Soc. Lond. A* **433**, 499–520 (1991).
